# Coadapted genomes and selection on hybrids: Fisher's geometric model explains a variety of empirical patterns

**DOI:** 10.1002/evl3.66

**Published:** 2018-08-14

**Authors:** Alexis Simon, Nicolas Bierne, John J. Welch

**Affiliations:** ^1^ Institut des Sciences de l'Évolution UMR5554, Université de Montpellier CNRS‐IRD‐EPHE‐UM France; ^2^ Department of Genetics University of Cambridge Downing St. Cambridge CB23EH United Kingdom

**Keywords:** Dobzhansky–Muller incompatibilities, Haldane's Rule, heterozygosity, inbreeding, speciation genetics, sterility

## Abstract

Natural selection plays a variety of roles in hybridization, speciation, and admixture. Most research has focused on two extreme cases: crosses between closely related inbred lines, where hybrids are fitter than their parents, or crosses between effectively isolated species, where hybrids suffer severe breakdown. But many natural populations must fall into intermediate regimes, with multiple types of gene interaction, and these are more difficult to study. Here, we develop a simple fitness landscape model, and show that it naturally interpolates between previous modeling approaches, which were designed for the extreme cases, and invoke either mildly deleterious recessives, or discrete hybrid incompatibilities. Our model yields several new predictions, which we test with genomic data from *Mytilus* mussels, and published data from plants (*Zea*, *Populus*, and *Senecio*) and animals (*Mus*, *Teleogryllus*, and *Drosophila*). The predictions are generally supported, and the model explains a number of surprising empirical patterns. Our approach enables novel and complementary uses of genome‐wide datasets, which do not depend on identifying outlier loci, or “speciation genes” with anomalous effects. Given its simplicity and flexibility, and its predictive successes with a wide range of data, the approach should be readily extendable to other outstanding questions in the study of hybridization.

**Impact summary**

When individuals of different populations mate, the offspring will carry new combinations of alleles. Sometimes the new combinations bring fitness benefits (heterosis). This is often true, for example, when the parental lines are closely related and highly inbred: a fact that can be exploited in artificial breeding programs. Sometimes, the hybrids are much less fit than their parents (hybrid breakdown), suggesting that the populations may be distinct species. These different outcomes depend on the ways in which the alleles interact, and so comparing the outcomes of different types of hybridization can tell us a lot about gene interactions. We developed a general mathematical multigenic model that makes simple predictions for the fitness of hybrids of any type. We show that our model can account for a large number of empirical patterns, including some that were not well explained by alternative theories, developed for specific cases. We tested our predictions with new data from mussels, and published data from plants and animals, and obtained a good fit. Our framework suggests a new and complementary approach to analyzing genomic data from hybrids, which does not rely on identifying small regions of the genome with anomalous effects.

Hybridization and admixture lead to natural selection acting on alleles in different genetic backgrounds. Most classical studies of hybridization can be placed into one of two classes. The first, involves crosses between closely related inbred lines, where there is no coadaptation between the deleterious alleles that differentiate the lines, such that most hybrids are fitter than their parents. Wright's single‐locus theory of inbreeding was developed to interpret data of this kind (Wright 1922, 1977; Crow 1952; Hallauer et al. 2010). The second, involves crosses between effectively isolated species, where viable and fertile hybrids are very rare. Data of this kind are often analyzed by focusing on a small number of “speciation genes,” and interpreted using models of genetic incompatibilities (Dobzhansky 1937; Coyne and Orr 1989; Orr 1995; Gavrilets 2004; Welch 2004; Kalirad and Azevedo 2017).

The differences between these types of hybridization are clear, but it is equally clear that they are extremes of a continuum. Furthermore, the intermediate stages of this continuum are of particular interest, because they include phenomena such as incipient speciation, and occasional introgression between partially isolated populations (Waser 1993; Rosas et al. 2010; Mendez et al. 2012; Fraïsse et al. 2016a; Duranton et al. 2017). However, it can be difficult to model natural selection in this intermediate regime, not least because models require a large number of parameters when they include epistatic effects between many loci. The empirical study of hybrid genotypes in this regime is also difficult. The analysis of lab crosses often focuses on segregation distortions of large effect, and pairwise incompatibilities (Coyne and Orr 2004; Abbott et al. 2013). This QTL‐mapping framework can miss small effect mutations (Noor et al. 2001; Rockman 2012), which are difficult to identify individually, but whose cumulative effect can be substantial (Boyle et al. 2017).

One promising approach is to use Fisher's geometric model, which assigns fitness values to genotypes using a model of optimizing selection on quantitative traits (Fisher 1930; Orr 1998; Welch and Waxman 2003; Martin and Lenormand 2006). The tools of quantitative genetics have often been used to study hybridization (e.g., Melchinger 1987; Lynch 1991; Demuth and Wade 2005; Fitzpatrick 2008), but Fisher's model is fully additive at the level of phenotype, and the “traits” need not correspond in any simple way to standard quantitative traits (Rosas et al. 2010; Martin 2014; Schiffman and Ralph 2017). Instead, the goal is to generate a rugged fitness landscape, which includes a wide variety of mutational effect sizes and epistatic interactions on fitness, with a minimum of free parameters (Orr 1998; Blanquart et al. 2014; Barton 2017; Hwang et al. 2017).

Here, we build on previous studies (Mani and Clarke 1990; Barton 2001; Rosas et al. 2010; Chevin et al. 2014; Fraïsse et al. 2016b; Schiffman and Ralph 2017), and use Fisher's geometric model to study hybridization. We develop a simple Brownian bridge approximation, and show that it can naturally interpolate between previous modeling approaches (Wright 1922, 1977; Orr 1995; Gavrilets 2004), which are appropriate for the two extreme types of hybridization. We then show how the model can account for surprising empirical patterns that have been observed in both regimes (Wright 1977; Moehring 2011; Moran et al. 2017). Finally, we show that the model yields novel predictions for selection on heterozygosity, and test these predictions with a wide range of new and existing datasets (Table 1).

**Table 1:** Datasets

Hybridization		*N*	Sex	#Markers	Cross	$g_{X}$	Fitness measure	Reference
*Zea mays*	inbred lines	–	⚥	–	Various	–	Excess yield	See Fig. 3
*Mytilus*	*edulis/galloprovincialis*	132	♀/♂	43	F2	–	–	This study
		144	♀/♂	43	BC1	–	–	
*Drosophila*	*pseudoobscura/persimilis*	1141/1036	♀	2 X; 11 A	BC1	0.37	Motile sperm: present/absent	Noor et al. (2001)
*Drosophila*	*sechellia/simulans*	200/200	♀	8 X; 31 A	BC1	0.17	Sperm quantity: 3‐pt. scale	Macdonald and Goldstein (1999)
*Drosophila*	*santomea/yakuba*	550/549	♀	10 X; 22 A	BC1	0.17	Motile sperm: 9‐pt. scale	Moehring et al. (2006a, 2006b)
*Teleogryllus*	*oceanicus/commodus*	248	♂	–	Various	0.30	Egg and offspring number	Moran et al. (2017)
*Populus*	*alba/tremula*	137	♀♂	∼12,000	WH	–	Survival after 4 years	Christe et al. (2016)
*Senecio*	*aethnensis/chrysanthemifolius*	64	⚥	966	F2	–	Necrotic/Healthy	Chapman et al. (2016)
*Mus musculus*	*musculus/domesticus*	185	♀	14,220	WH	0.039	Testes weight	Turner and Harr (2014)
		305	♀	202 (16 X; 182 A)	F2	0.039	Prop. abnormal sperm	White et al. (2011)

*N*, The number of individual hybrids, divided by backcross direction where appropriate; #Markers, The number of genetic markers used to estimate *p*_12_ and *h*, sometimes divided into X‐linked and Autosomal. BC1, First backcross; WH, Wild hybrids. $g_{X}$, weight given to X‐linked markers in the calculation of overall genome composition, calculated from the length of annotated coding sequence.

## Models and Results

### THE MODELS

#### Notation and basics

We will consider hybrids between two diploid populations, labeled P1 and P2, each of which is genetically uniform, but which differ from each other by *d* substitutions. Considering all possible combinations of the two homozygotes and the heterozygote, the populations could generate 3^*d*^ distinct hybrid genotypes, and each might differ in their overall fitness, or in some component of fitness, such as fertility or viability. Below, we will focus on rank order differences between different classes of hybrid (e.g., high vs. low heterozygosity, males vs. females, F1 vs. F2 etc.). As such, following Turelli and Orr (2000), we describe hybrids using a “breakdown score,” *S*, which is larger for hybrids that have lower values of the fitness component of interest. Breakdown might relate to fitness via,

(1) $$\ln w\propto -S^{\alpha /2}$$

in which case, the parameter α adjusts the rate at which fitness declines with breakdown. This is related to the overall levels of fitness dominance and epistasis (Hinze and Lamkey 2003; Tenaillon et al. 2007; Fraïsse et al. 2016b), and so these can vary independently of other results. We now define the key quantity *f*, as the expected value of *S* for a given class of hybrid, scaled by the expected value for an unfit reference class.

(2) $$f\equiv \frac{E(S)}{E(S_{†})}$$

Here, $E(S_{†})$, is the expected breakdown score for the class of hybrid with the lowest fitness, on the condition that the parental genotypes are optimally fit. In this case, *f* can vary between zero, for the best possible class of hybrid, and one, for the worst possible class. We will also consider the case where the parental types are themselves suboptimal, with their own level of “breakdown,” denoted *f*_P1_ and *f*_P2_. In this case, when $f_{P1},f_{P2}>0$, then the value of *f* for the hybrids can be greater than one (see below).

To define classes of hybrid, we also follow Turelli and Orr (2000). We pay particular attention to interpopulation heterozygosity, and define *p*_12_ as the proportion of the divergent sites that carry one allele from each of the parental types. We also define *p*_1_ and *p*_2_ as the proportion of divergent sites that carry only alleles originating from P1 or P2, respectively. Since $p_{1}+p_{2}+p_{12}=1$, it is convenient to introduce the hybrid index, *h*, which we define as the total proportion of divergent sites originating from P2 (Barton and Gale 1993).

(3) $$h\equiv p_{2}+\frac{1}{2}p_{12}$$

**Figure 1:**
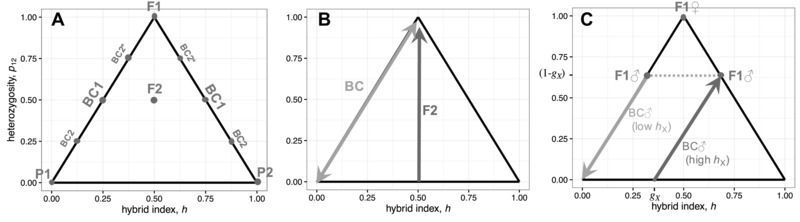
Diploid hybrid genomes can be represented as points in a triangular space, indicating their hybrid index, *h* (the total proportion of divergent alleles that originate from parental line P2), and their interpopulation heterozygosity, *p*_12_ (the proportion of divergent sites that carry one allele from each parental line). Panel (**A**) shows how the standard crosses are placed in this space, with biparental inheritance. The two parental lines P1 and P2 are at the lower corners. The initial F1 cross (P1 × P2) will be heterozygous at all divergent sites, and so be found at the top corner. Individuals from later crosses will vary due to segregation and recombination, but the F2 (F1 × F1) will tend to be found toward the center, while backcrosses (such as  BC 1=F1×P1 or F1 × P2) will be found on the upper edges (see Fig. 3 for more details of these backcrosses). Panel (**B**) illustrates the selection on heterozygosity predicted by Fisher's geometric model, when the parental lines are well adapted (eq. 9). In backcrosses, heterozygosity will be under diversifying selection, favoring both extreme values. By contrast, in the F2, we predict directional selection toward higher heterozygosity. Panel (**C**) illustrates some complications introduced by heteromorphic sex chromosomes (see eq. 16 and Appendix 4). With XO sex determination, male F1 carry no heterozygosity on the X, which will tend to reduce their fitness, consistent with Haldane's Rule. For male backcrosses (F1 × P1), the selection acting on (autosomal) heterozygosity, will depend on the alleles carried on the X. When the X carries mostly P2 alleles, fitter individuals will be more heterozygous (darker gray arrow). When the X carries mostly P1 alleles, the fittest individuals will carry no heterozygosity (lighter gray arrow). $g_{X}$ is the proportion of the divergent sites found on the X, and is set at 37%, as we have estimated for *Drosophila pseudoobscura*.

This quantity is closely related to measures of ancestry (e.g., Falush et al. 2003; Fitzpatrick 2012; Christe et al. 2016), although it considers only divergent sites. We can now describe each individual genotype via its heterozygosity, *p*_12_, and hybrid index, *h*. This means that all hybrids can be represented as points in a triangular space, as shown in Figure 1 A (Gompert and Buerkle 2010; Fitzpatrick 2012). Our goal in this article is to represent this space as a kind of fitness landscape, and show how *f* can vary with *p*_12_ and *h*.

#### Fisher's geometric model

**Figure 2:**
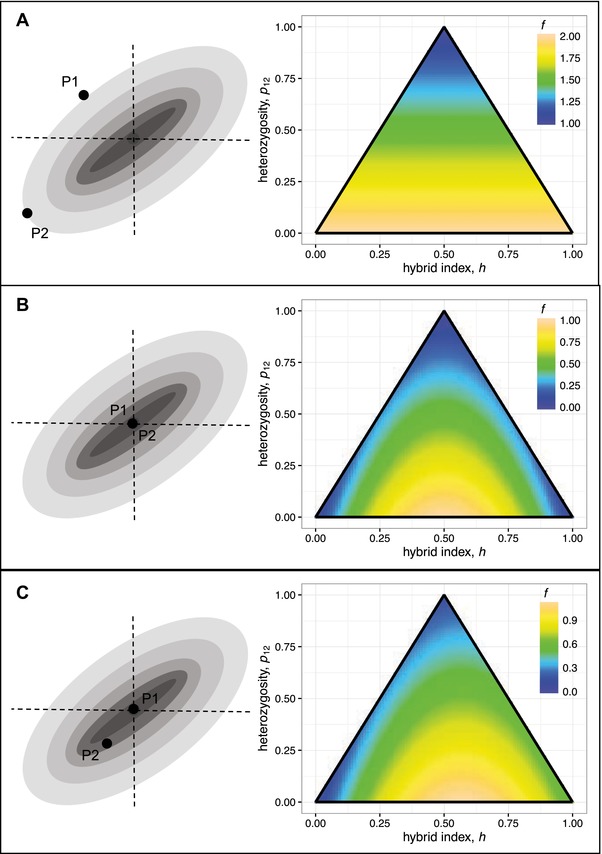
Fisher's geometric model generates a flexible series of fitness landscapes for hybrid genotypes. The left‐hand panels show a schematic representation of the model, with n=2 traits, each under optimizing selection of differing strengths. (The orientation of the axes shows that the model allows for correlated selection, although this is ignored in the text, by rotating the axes). The right‐hand panels show how the expected breakdown score (eqs. 2 and 6), varies for hybrids of different types. Panels (**A**)–(C) show different assumptions about levels of parental maladaptation. Panel (A) shows a scenario where all of the parental divergence is maladaptive, with no tendency for their alleles to be coadapted. In this case, hybrid fitness increases with their heterozygosity, as predicted by Wright's single‐locus theory of inbreeding (eq. 8; Wright 1922, 1977). Panel (**B**) shows a scenario where the parental lines are optimal (or, at least, very well adapted compared to the worst class of hybrid that can be formed between them). In this case, the hybrid index is under symmetrical diversifying selection, and the form of selection on heterozygosity will vary for different cross types (eqs. 9, 13, and 15). This landscape can also be generated by a general model of genetic incompatibilities (see Appendix 2). Panel (C) shows a situation where only one of the parental lines is maladapted (eq. 10 with $f_{P2}=0.5$).

Fisher's model is defined by *n* quantitative traits under selection toward an intermediate optimum (Fisher 1930). We will assume that fitness is always measured in a fixed environment, but we make no assumptions about how this optimum might have moved during the parental divergence (see Appendix 1). If the selection function is multivariate normal, including correlated selection, then we can rotate the axes and scale the trait values, to specify *n* new traits that are under independent selection of different strengths (Waxman and Welch 2005; Martin and Lenormand 2006; Martin 2014). Examples with n=2 traits are shown in Figure 2. We now define the breakdown score of a phenotype as

(4) $$S\equiv \sum_{i=1}^{n}\lambda_{i}z_{i}^{2}$$

where, for trait *i*, $z_{i}$ is its deviation from the optimum and $\lambda_{i}$ is the strength of selection. By assumption, all mutational changes act additively on each trait, but their effects on fitness can vary with the phenotype of the individual in which they appear, and this yields fitness dominance and epistasis (Appendix 1).

The *d* substitutions that differentiate P1 and P2 can be considered as a chain of vectors, which connect the two parental phenotypes (Fig. S1). While the sizes and directions of these vectors will generally be unknown, in Appendix 1, we show that the chain can be approximated as a constrained random walk, or Brownian bridge (Revuz and Yor 1999, Ch. 1). This approximation relies on the fact that hybrid genomes contain the fixed alleles in effectively random combinations, and it gives accurate results for a wide range of assumptions about the divergence process (Figs. S2–S3), including adaptation of the parental lines to different environments (Appendix 1; Fig. S4).

Under the Brownian bridge approximation, the quantity $E(S_{†})$, that appears in equation (2), is given by

(5) $$E\left(S_{†}\right)=d\sum_{i=1}^{n}\lambda_{i}v_{i}$$

where $v_{i}$ is the expected variance contributed to trait *i* by a fixed mutation in heterozygous state and we have assumed that any maladaptation has accrued independently in the two parental lines (see Appendix 1 and Table S1). The key quantity *f* is given by

(6) $$f=f_{P1}+\beta_{1}h\left(1-\beta_{2}h\right)-p_{12}$$

where

(7) $$\begin{array}{ccc} \beta_{1} & \equiv & 4-2f_{P1}\\ \beta_{2} & \equiv & \frac{4-f_{P1}-f_{P2}}{4-2f_{P1}} \end{array}$$

(see Appendix 1 for full details). Two features of equation (6) are immediately notable. First, it does not depend on any of the model parameters. For example, the number of traits, *n*, could affect the accuracy of our approximation (since *S* will tend to approach normality as *n* increases); but it does not appear in equation (6), which depends solely on *p*_12_, *h* and the levels of parental maladaptation. Second, for a given value of *h*, the fitness of hybrids will tend to increase with their heterozygosity, *p*_12_. This prediction agrees with the widespread finding of heterosis (Crow 1952; Table S1 of Fraïsse et al. 2016b). Indeed, by rearranging equation (6) it is clear that hybrids will tend to be fitter than parental line P1 (such that $f<f_{P1}$) on the condition that $p_{12}>\beta_{1}h\left(1-\beta_{2}h\right)$.

It is also useful to consider equation (6) in a few special cases. First, let us assume that all of the divergence between the parents is maladaptive, without any tendency for coadaptation between their alleles. In this case, the parental phenotypes can be treated as unconstrained random walks away from the optimum. Each fixed mutation, in homozygous state, will contribute an expected $4v_{i}$ to the variance on each trait, and so, from equations (4) and (5), we have $E\left(f_{P1}\right)=E\left(f_{P2}\right)=2$ (see also Appendix 1). With this high level of parental maladaptation, the expected breakdown score of the hybrids is

(8) $$f=2-p_{12},\;f_{P1,}f_{P2}=2$$

The fitness landscape implied by equation (8) is illustrated in Figure 2 A. With these extreme levels of parental maladaptation, hybrid breakdown depends only on the heterozygosity. Indeed, this result is closely related to Wright's (1922) single‐locus theory of inbreeding, which was developed to analyze crosses between closely related inbred lines, where all divergence between the parental lines comprises deleterious alleles. Wright's theory therefore appears as a special case of Fisher's geometric model, when parental alleles show no coadaptation (see below).

Now let us consider the other extreme case, where the parental alleles are completely coadapted, such that P1 and P2 both have optimal fitness, but realized by different combinations of alleles. In this case, we find

(9) $$f=4h\left(1-h\right)-p_{12},\;f_{P1,}f_{P2}=0$$

The fitness landscape implied by equation (9) is illustrated in Figure 2 B. With well‐adapted parents, the hybrid index is under symmetrical diversifying selection.

As with equation (8), equation (9) can also be derived via an alternative route, using a model of speciation genetics. In particular, we show in Appendix 2 that equation (9) can be obtained from a general model of “Dobzhansky–Muller incompatibilities,” each involving a small number of loci (Orr 1995; Turelli and Orr 2000; Gavrilets 2004; Welch 2004; Fraïsse et al. 2016b). The agreement between the two models depends on a particular set of assumptions about the dominance of incompatibilities, namely (i) partial recessivity on average, and (ii) greater reduction in fitness when they contain homozygous alleles from both parental lines. However, we show in Appendix 2 that these assumptions are biologically realistic, and implied by a number of well‐established empirical patterns (Turelli and Orr 2000). The result is that equation (9) can be interpreted as a model of genetic incompatibilities, but without the large number of free parameters that these models can require.

Equation (9) is expected to apply to many cases of wild hybridization, because it should provide a good approximation even if the parental populations are not truly optimal. The only requirement is that they be much better adapted than the worst possible class of hybrid, such that $f_{P1},f_{P2}\ll 1$.

Nevertheless, the general form of equation (6) also allows us to explore intermediate cases. For example, if only P2 is maladapted, then we find

(10) $$f=4h\left(1-\left(1-\frac{1}{4}f_{P2}\right)h\right)-p_{12},\;f_{P1}=0$$

In this case, as illustrated in Figure 2 C, selection on hybrid index is skewed toward the fitter parent. Below, we will show how all three of these special cases (Fig. 2 A–C) can be used in data analysis.

### TESTING THE PREDICTIONS WITH BIPARENTAL INHERITANCE

#### Fitness differences between crosses

The simplest predictions from equation (6) assume standard biparental inheritance at all loci. In this case, the standard cross types can be easily located on our fitness landscapes. This is shown in Figure 1 A.

With well‐adapted parental lines (Fig. 2 B) hybrids of high fitness are expected only for the initial F1 cross (P1 × P2) and breakdown is predicted for later crosses, such as the first backcross (BC1: F1 × P1 or F1 × P2) or the F2 (F1 × F1). This pattern of F1 hybrid vigor (high F1 fitness) followed by breakdown in later crosses, has widespread empirical support (see references in Table S1 of Fraïsse et al. 2016b and Rosas et al. 2010).

With highly maladapted parents, by contrast, hybrids of all types can be fitter than their parents (see Fig. 2 A). Plentiful data of this kind come from highly inbred lines of *Zea mays* (Neal 1935; Wright 1977; Melchinger 1987; Hinze and Lamkey 2003; Hallauer et al. 2010). To analyze these data, a widely used proxy for fitness is the excess yield of a cross (i.e., its increase in yield relative to the parental lines), scaled by the excess yield of the F1. From equations (1)–(2), and using a Taylor expansion, we find

(11) $$\frac{w-w_{P}}{w_{F1}-w_{P}}\approx \frac{f_{P}^{\alpha /2}-f^{\alpha /2}}{f_{P}^{\alpha /2}-f_{F1}^{\alpha /2}}$$

(12) $$=p_{12},\;f_{P}=2,\alpha =2$$

where the subscript P refers to both parental lines, which are assumed to have similar yields. For later crosses, these values will vary between individuals within a cross, due to segregation and recombination, but in this section we ignore this variation, and assume that *p*_12_ and *h* take their expected values for a given cross type (Fig. 1 A). A fuller treatment is outlined in Appendix 3.

**Figure 3:**
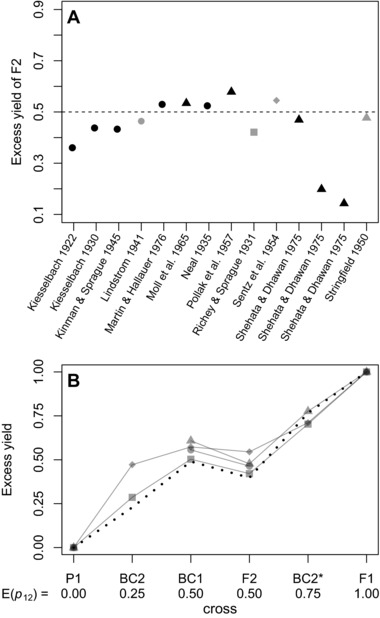
Data on hybrid vigor (i.e., increased yield), from crosses of inbred *Zea mays*. The original data were collated by Wright (1977; see his Table 2.23), and Hallauer et al. (2010; see their Table 9.13), including only data from single crosses, where there was hybrid vigor in the F2, and yield measured in quintals per hectare. Panel (**A**) plots the excess yield of the F2 (eq. 11). Results are shown for variety crosses (black triangles), as well as crosses of inbred lines in the strict sense (all other points). The dashed line shows the prediction of 0.5 from single‐locus theory (eq. 12). Panel (B) shows the four datasets collated by Wright (1977), which allow us to compare the F2 and various backcrosses. These crosses, chosen to yield different levels of heterozygosity, are the parental type (P1), the second backcross ( BC 2=(F1×P1)×P1); the first backcross ( BC 1=F1×P1), the F2 (F1 × F1), second backcross to the other parent ( BC 2∗=(F1×P1)×P2), and the F1 (P1 × P2) (The data of Stringfield (1950), shown as gray triangles, replace BC2* with an F2 between two distinct F1, involving three distinct strains, but the predictions are unchanged). The gray symbols for the four datasets correspond to those used in panel (**A**). The dotted line in panel (**B**) shows predictions from Fisher's model, assuming that the between‐strain divergence contains limited coadaptation. The prediction uses equations (13), (14), and (11), with $f_{P}=1.5$, and α=3. The model predicts both the roughly linear increase in vigor with heterozygosity, and the systematic difference between BC1 and F2.

Equation (12) confirms that Fisher's model can produce Wright's (1922) single‐locus predictions for inbreeding, but only when all divergence between the parental lines is deleterious ($f_{P}=2$), and when increases in breakdown score act independently on log fitness (α=2). These single‐locus predictions have strong support in *Zea mays* (Neal 1935; Wright 1977; Melchinger 1987; Hinze and Lamkey 2003; Hallauer et al. 2010). For example, as shown in Figure 3 A, the excess yield of the F2 is often around 50%, which is equal to its expected heterozygosity (Wright 1977; Hallauer et al. 2010). It is also notable that the two outlying points (from Shehata and Dhawan 1975), are variety crosses, and not inbred lines in the strict sense.

Despite this predictive success, Wright (1977) noted a pattern that single‐locus theory could not explain. In Wright's words: “the most consistent deviation from expectation [...] is the low yield of F2 in comparison with the first backcrosses” (Wright 1977, p. 39). Because $E\left(p_{12}\right)=\frac{1}{2}$ for both crosses, this cannot be explained without epistasis, or coadaptation between the alleles in the parental lines. In fact, the pattern is predicted by Fisher's model, when there is a small amount of coadaptation, such that $1<f_{P}<2$. This yields a fitness surface with a small amount of curvature, which is intermediate between those shown in Figure 2 A and 2 B.

Figure 3 B plots the four relevant datasets collated by Wright, and compares the results to predictions from equations (6) and (11), with $f_{P}=1.5$ and α=3. The model predicts the roughly linear increase in yield with mean heterozygosity, as with single locus theory, but also predicts the consistent difference between BC1 and the F2.

#### Selection on heterozygosity within crosses

In the previous section, we ignored between‐individual variation in heterozygosity within a given cross type. In this section, we show how natural selection is predicted to act on this heterozygosity.

First, let us consider the F2. In this case, we have 4h(1−h)≈1 with relatively little variation between individuals (see Appendix 3 for details). Therefore, if both parents have similar levels of maladaptation, equation (6) is well approximated by

(13) $$f_{F2}\approx 1+\frac{1}{2}f_{P}-p_{12}$$

The prediction is that *p*_12_ will be under directional selection in the F2, favoring individuals with higher heterozygosity. This is illustrated in Figure 1 B.

Now let us consider a backcross: F1 × P1. In this case, all of the homozygous alleles must come from P1, such that $4h\left(1-h\right)=p_{12}\left(2-p_{12}\right)$, and so equation (6) becomes

(14) f BC =fP+(1−fP)p12−1−12fPp122

(15) $$=p_{12}\left(1-p_{12}\right),\;f_{P}=0$$

So backcrosses will tend to be least fit when they have intermediate levels of heterozygosity. When parents are well adapted, heterozygosity is under symmetrical disruptive selection, favoring heterozygosities that are either higher or lower than $p_{12}=0.5$ (eq. 15). This is illustrated in Figure 1 B .

To test these predictions, we used a new set of genetic data from hybrids of the mussel species: *Mytilus edulis* and *Mytilus galloprovincialis* (Bierne et al. 2002, 2006). These species fall at the high end of the continuum of divergence during which introgression persists among incipient species (Roux et al. 2016). We used experimentally bred F2 and BC1, with selection imposed implicitly, by the method of fertilization, and by our genotyping only individuals who survived to reproductive age (Bierne et al. 2002, 2006; see Methods and Fig. S6 for full details).

To estimate heterozygosity in each hybrid individual, we used the 43 markers that were heterozygous in all of the F1 hybrids used to make the subsequent crosses (see Fig. S6). We then asked whether the distribution of *p*_12_ values in recombinant hybrids was symmetrically distributed around its Mendelian expectation of $p_{12}=0.5$, or whether it was upwardly biased, as would be expected if directional selection were acting on heterozygosity. As shown in the first column of Table 2, Wilcoxon tests found that heterozygosities in surviving hybrids were significantly higher than expected, in both the F2 and backcross. These results may have been biased by the inclusion of individuals with missing data, because they showed higher heterozygosity (see Table S2). We therefore repeated the test with these individuals excluded. As shown in the second column of Table 2, results were little changed, although the bias toward high heterozygosities was now weaker in the backcross.

**Table 2:** Tests for selection on heterozygosity in F2 and Backcrosses of *Mytilus* mussels

Markers:	43	43	33	33
Dataset:	All	No missing data	No missing data	Subsampled
Cross	${\hat{p}}_{12}$ (*N*) *P*‐value	${\hat{p}}_{12}$ (*N*) *P*‐value	${\hat{p}}_{12}$ (*N*) *P*‐value	${\hat{p}}_{12}$ (*N*) *P*‐value
F2	0.57 (132) $1.5\times 10^{-6}$ ***	0.56 (88) $6.4\times 10^{-4}$ ***	0.55 (91) 0.0033**	0.56 (56) 0.0020**
BC	0.57 (144) $1.3\times 10^{-5}$ ***	0.53 (94) 0.0282*	0.53 (105) 0.0569	0.52 (56) 0.5815

${\hat{p}}_{12}$, the estimated median heterozyosity; *N*, the number of hybrid individuals sampled; *P*‐value, result of a Wilcoxon test of the null hypothesis median $p_{12}=0.5$ (* P<0.05; ** P<0.01; *** P<0.001). F2, random mating of F1 between *M. galloprovincialis* and *M. edulis*; BC, Backcross of the F1 to *M. galloprovincialis*. No missing data, all individuals with missing data for any of the markers were excluded; Subsampled, for the BC, any individual carrying a marker that was homozygous for the major allele carried by wild *M. edulis* populations was excluded; for the F2, we downsampled by sequencing order to equalize sample sizes.

Interpreting these results is complicated by the ongoing gene flow between *M. edulis* and *M. galloprovincialis* in nature (Fraïsse et al. 2016a). To test for this, we genotyped 129 pure‐species individuals, and repeated our analyses with a subset of 33 markers that were strongly differentiated between the pure species (see Methods, Fig. S6 and Table S3 for details). With these markers, there was evidence of elevated heterozygosity in the F2, but not the backcross (Table 2 third column). We also noticed that many of our backcross hybrids, though backcrossed to *M. galloprovincialis*, carried homozygous alleles that were typical of *M. edulis*. We therefore repeated our analysis after excluding these “F2‐like” backcrosses. Results, shown in the fourth column of Table 2, showed that the reduced BC dataset showed no tendency for elevated heterozygosity. However, the bias toward higher heterozygosities remained in the F2, even when we subsampled to equalize the sample sizes.

Despite the problems of interpretation due to introgression and shared variants, the results support the prediction of equations (13)–(15): that directional selection on heterozygosity should act in the F2, but weakly or not at all in the backcross.

### PREDICTIONS OF FISHER'S GEOMETRIC MODEL WITH SEX‐SPECIFIC INHERITANCE

Results above assumed exclusively biparental inheritance. But the predictions of Fisher's model are easily extended to include heteromorphic sex chromosomes, or loci with strictly uniparental inheritance, such as organelles (Coyne and Orr 1989; Turelli and Orr 2000; Turelli and Moyle 2007; Fraïsse et al. 2016b). In this section, we introduce the approach, and demonstrate its flexibility, while the full derivations are collected in Appendix 4. The basic method of introducing sex‐specific inheritance is to write *p*_12_ and *h* as weighted sums of contributions from different types of locus. For example, to focus on the contribution of the X chromosome versus the autosomes, we can write

(16) $$\begin{array}{ccc} p_{12} & = & g_{X}\;p_{12,X}+g_{A},p_{12,A}\\ h & = & g_{X}\;h_{X}+g_{A}\;h_{A} \end{array}$$

where the subscripts denote the chromosome type (so that $p_{12,A}$ is the proportion of divergent sites on the autosomes that are heterozygous), and $g_{X}$ and $g_{A}$ are weightings, which sum to one (Turelli and Orr 2000).

We can now predict differences in hybrids of different sexes. For example, previous authors have shown that Fisher's model predicts Haldane's Rule: the tendency of sex‐specific breakdown to appear in the heterogametic sex (Haldane 1922; Turelli and Orr 2000; Barton 2001; Fraïsse et al. 2016b; Schiffman and Ralph 2017; see Fig. 1 C). Using equations (16) and (6), this basic insight can easily be extended to give formal conditions for Haldane's Rule, with different levels of parental maladaptation, and uniparental inheritance (see Appendix 4).

Sex chromosomes also complicate predictions about selection on heterozygosity within crosses. Consider, for example, male backcrosses in an XY species, where the X chromosome is large, the Y chromosome is small or degenerate, and the parental lines are reasonably fit. These backcross males will vary in their autosomal heterozygosity ($p_{12,A}$), but the selection on this heterozygosity will vary with the alleles carried on the X. This is illustrated in Figure 1 C. If the backcross is made to P1, but $h_{X}$ is large (i.e. divergent X‐linked alleles come mainly from P2), then heterozygosity will tend to be under positive selection (see darker arrow in Fig. 1 C); but if $h_{X}$ is small, then heterozygosity will tend to be under negative selection (see lighter arrow in Fig. 1 C, Appendix 4 and Fig. S8 for details).

To test this prediction, we used data from Noor et al. (2001) (Fig. S7). These authors generated male backcrosses between *Drosophila pseudoobscura* and *Drosophila persimilis*. These species are ideal for our test, because the X carries ∼37% of the coding sequence, the Y is degenerate, and the hybrids can be fully sterile (see Table 1, Methods, and Noor et al. 2001). Each backcross male was scored for sterility, and multiple genetic markers, including two markers on the X (see Table 1). We characterized the X as low‐$h_{X}$ if both markers carried the P1 allele, and as high‐$h_{X}$ if both markers carried the P2 allele; we excluded intermediate individuals, for which we have no clear prediction. We then regressed sterility on heterozygosity, $p_{12,A}$, but allowed the intercept and the slope to vary with $h_{X}$. Results, shown in Table 3, show that both $h_{X}$ and $p_{12,A}$ are important predictors of sterility in these backcrosses, and that their effects interact. With low $h_{X}$, sterility is generally rarer, but positively correlated with $p_{12,A}$. With high $h_{X}$, by contrast, sterility is more common, and negatively correlated with $p_{12,A}$. The predictions of Fisher's model are therefore supported, in both cross directions.

**Table 3:** Regressions of male sterility on autosomal heterospecificity in *Drosophila* backcrosses

Backcross to	*N*	Model	intercept (low $h_{X}$)	intercept (high $h_{X}$)	Best‐fit coefficients for $p_{12,A}$	AIC
*D. persimilis*	577	two intercepts	−1.071	1.797	–	596.79
		two intercepts + one slope	−2.165	0.764	2.147	580.41
		two intercepts + two slopes	−3.033	2.871	3.746 (low‐$h_{X}$); −1.973 (high‐$h_{X}$)	**558.91**
*D. pseudoobscura*	610	two intercepts	−0.197	2.962	–	603.53
		two intercepts + one slope	−2.031	1.219	3.620	558.18
		two intercepts + two slopes	−2.485	4.034	4.505 (low‐$h_{X}$); −1.876 (high‐$h_{X}$)	**545.87**

AIC, Akaike Information Criterion; preferred model shown in bold.

The interaction between $h_{X}$ and $p_{12,A}$ may also help to explain a broader set of patterns observed by Moehring (2011) in multiple datasets of *Drosophila* backcrosses (Macdonald and Goldstein 1999; Noor et al. 2001; Moehring et al. 2006a, 2006b and see Table 1). Moehring found that male fertility correlated strongly and negatively with $h_{X}$, while correlations with $p_{12,A}$ were weak and inconsistent. This pattern was not consistent with predictions of a simple model of X‐autosome incompatibilities (Moehring 2011), but it is consistent with Fisher's geometric model (see Fig. 1 C, Fig. 2 B, and Appendix 4).

#### Female backcrosses in *Teleogryllus*

Fisher's model can help to explain more complex patterns. To illustrate this, we will consider the data of Moran et al. (2017) from the field crickets *Teleogryllus oceanicus* and *T. commodus*. These species have XO sex determination, and a large X chromosome ($g_{X}\approx 0.3$, from Moran et al. 2017). They are also a rare exception to Haldane's Rule, with F1 sterility appearing solely in XX females (Hogan and Fontana 1973). Moran et al. (2017) hypothesized that female sterility might be caused by negative interactions between heterospecific alleles on the X, which appear together in F1♀, but not in F1♂.

**Figure 4:**
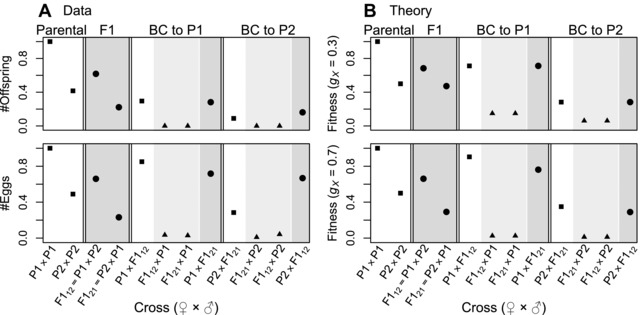
Data on female fertility, from crosses of the field crickets *Teleogryllus oceanicus* (P1), and *T. commodus* (P2), from Moran et al. (2017), compared to theoretical predictions from Fisher's geometric model. Plotting styles denote the level of X‐linked heterozygosity: high (circles and darker shading); intermediate (triangles and lighter shading) or low (squares and no shading). To plot the data (column **A**), we used the mean number of offspring per pair (upper panel), or mean number of eggs per pair (lower panel), each normalized by the value for P1 (see the supplementary information of Moran et al. 2017 for full details). The theoretical predictions (column **B**) are listed in Table S4. In the upper panel, we assume $g_{X}=0.3$, as estimated from the chromosome sizes, and complete silencing of the paternal X (such that π=1 in Table S4). In the lower panel, we assume $g_{X}=0.7$, and incomplete silencing of the paternal X (π=0.8), to improve fit to the egg data. While predictions apply to the rank order of fitnesses, to aid visualization, we plot $w=e^{-6f^{2}}$ (see eq. 1), and set the parameter *f*_P2_ via $0.5=e^{-6f_{P2}}$, to reflect the lower fertility of this species under lab conditions.

To test this hypothesis, they generated backcross females with identical recombinant autosomes, but different levels of heterozygosity on the X, $p_{12,X}$. The hypothesis of X–X incompatibilities predicts that fertility will decrease with $p_{12,X}$, but this was not observed. This is shown in Figure 4 A.

To try and explain the patterns that were observed, let us note two clear asymmetries in the data. First, there are strong differences between the fitness levels of the two parental species in lab conditions, with *T. commodus* (labeled P2 in Fig. 4) producing around half the eggs and offspring of *T. oceanicus* (P1 in Fig. 4). This suggests that equation 10 might apply to these data. Figure 4 shows a second asymmetry in the data: the reciprocal F1 (female–male vs. male–female) have very different fitness (Turelli and Moyle 2007; Fraïsse et al. 2016b). Because the X and autosomes will be identical for both cross directions, this implies some sort of parent‐of‐origin effect on the phenotype. This could be either uniparental inheritance, or selective silencing (Turelli and Moyle 2007; Fraïsse et al. 2016b). One possibility is the speculation of Hoy and collaborators (see e.g., Hoy et al. 1977; Butlin and Ritchie 1989; Dr. Peter Moran pers. comm.), that dosage compensation in *Teleogryllus* involves silencing of the paternal X.

We can now use the foregoing assumptions to predict the levels of breakdown for each of the crosses produced by Moran et al. (2017). The predictions for each cross are listed in Table S4, and plotted in the upper panel of Figure 4 B. This simple model explains several striking aspects of the *Teleogryllus* data.

Adjusting the parameter values can further improve the fit. For example, if we increase $g_{X}$ (as would be case if divergent sites affecting female fecundity were clustered on the X), and assume that paternal X silencing is incomplete (affecting 80% of the divergent sites), then the results, shown in the lower panel of Figure 4 B, agree well with the data on *Teleogryllus* egg number, as shown in the lower‐left‐hand panel (only the high fitness of P2 × F1_12_ is poorly predicted).

Further adjustments are possible, but these soon become *ad hoc*, at least without further knowledge of the true nature of parent‐of‐origin effects in *Teleogryllus*. The important point here is that Fisher's geometric model explains several key features of these hybrid data, while using only a single parameter derived from the data themselves; and even this parameter (*f*_P2_), was estimated from the parental control lines.

### ESTIMATING THE FITNESS SURFACE

Across a diverse collection of hybrids, equation (6) predicts that the hybrid index will be under disruptive selection, and heterozygosity under directional selection. This prediction can be tested with datasets containing estimates of fitness, *h* and *p*_12_ for many hybrid individuals. Exactly such an analysis was presented by Christe et al. (2016), for families of wild hybrids from the forest trees, *Populus alba* and *P. tremula* (Lindtke et al. 2012, 2014; Christe et al. 2016). These authors scored survival over four years in a common‐garden environment, and fit a generalized linear model to their binary data (binary logistic regression, with “family” as a random effect), and predictors including linear and quadratic terms in *p*_12_ and *h*. Model selection favored a four‐term model, with terms in *p*_12_, *h*, and *h* ^2^ (see Table S6, and Supplementary information of Christe et al. 2016 for full details). For comparison with our theoretical predictions, we can write their best fit model in the following form:

(17) $$y=const+\beta_{0}\left(\beta_{1}h\left(1-\beta_{2}h\right)-p_{12}\right)$$

where *y* is the fitted value for hybrid breakdown. In its general form, equation (6) should hold and Fisher's model predicts that $\beta_{0}>0$, $0\leq \beta_{1}\leq 4$, and $\frac{1}{2}\leq \beta_{2}\leq 2$. Additionally if parental maladaptation is not highly asymmetrical then it predicts $\beta_{2}\approx 1$.

The best‐fit model of Christe et al. (2016) corresponds to ${\hat{\beta }}_{0}=2.963$, ${\hat{\beta }}_{1}=2.777$ and ${\hat{\beta }}_{2}=0.934$, which supports the predictions of directional selection toward higher heterozygosity, and near‐symmetrical diversifying selection on the hybrid index.

To obtain confidence intervals on these parameters, we fit equation (17) to the raw data of Christe et al. (2016). We also searched for other data sets, from which we could estimate the hybrid fitness surface. After applying some quality controls (see Methods and Table S2), we identified one other dataset of wild hybrids, from the mouse subspecies *Mus musculus* *musculus/domesticus*, where male testes size was the proxy for fertility (Turner and Harr 2014). We also found four datasets of controlled crosses: F2 from the same mouse subspecies (White et al. 2011), and the ragworts *Senecio aethnensis* and *S. chrysanthemifolius* (Chapman et al. 2016); and the *Drosophila* backcrosses discussed above (Macdonald and Goldstein 1999; Moehring et al. 2006a, 2006b). Unlike the data from wild hybrids, these single‐cross datasets leave large regions of the fitness surface unsampled (see Fig. 1); nevertheless, they each contain enough variation in *h* and *p*_12_ for a meaningful estimation. Details of all six datasets are shown in Table 1, and they are plotted in Figures S9–S11.

**Figure 5:**
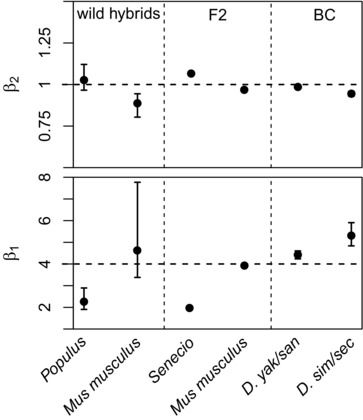
Best fit parameter estimates for the GLM of equation (17), with fitness and genomic data from six sets of hybrids (see Table 1 for details). The upper panel shows estimates of the coefficient β_2_ that determines the form of selection acting on the hybrid index, *h*. Estimates of $\beta_{2}=1$ are consistent with symmetrical diversifying selection. The lower panel show estimates of the coefficient β_1_ that determine the relative strength of selection acting on the hybrid index. Estimates of $\beta_{1}=4$ are predicted when the parental types are well adapted (eq. 9), while estimates $0<\beta_{1}<4$ are predicted when the parental types are maladapted (eq. 6). Confidence intervals are defined as values that reduce the AIC by 2 units. These measures of uncertainty were not obtained for the F2 data, where variation in the hybrid index contributed little to the model fit, as predicted by equation (13). Full details of the model fitting are found in the Methods and Tables S5 and S6.

Figure 5 shows a summary of the estimated parameters, and full results are reported in Tables S5 and S6, and Figures S9–S11. Taken together, the results show good support for the predictions of equation (6). For all six datasets there was evidence of significant positive selection on heterozygosity (${\hat{\beta }}_{0}>0$ was preferred in all cases). Furthermore, for all six datasets, we inferred diversifying selection acting on the hybrid index. Estimates of β_2_, shown in the upper panel of Figure 5, show that this selection was near‐symmetrical in all cases, such that ${\hat{\beta }}_{2}\approx 1$. The poorest fit to the predictions was found for the *Drosophila* backcrosses, where estimates of β_1_ were significantly greater than the predicted upper bound of $\beta_{1}=4$ (Fig. 5 lower panel). But these datasets were least suited to our purpose, because estimates of *h* and *p*_12_ depend strongly on our rough estimate of the relative contributions of the X and autosomes (see Methods), and because they lack F2‐like genotypes, from the center of the fitness surface (Fig. 1 A; Fig. S11). By contrast, results for the *Mus musculus* F2 (White et al. 2011), are remarkably close to the predictions of equation (9) (Fig. 5; Fig. S10).

Two other features of the results deserve comment. First, for the two F2 datasets, it was not possible to provide meaningful confidence intervals for β_1_ and β_2_. This is because, for these two datasets, the terms in *h* and *h* ^2^ did not make a significant contribution to model fit, and so the preferred model contained only selection on *p*_12_ (see Table S6). This is consistent with our earlier prediction of equation (13), and stems from the low variation in 4h(1−h) among F2 hybrids (see Appendix 3 and Figs. S9 and   S10).

Second, for two of the datasets, *Populus* and *Senecio*, the estimates of β_1_ are substantially lower than 4 (Fig. 5; Fig. S9). This is suggestive of parental maladaptation, creating true heterosis in the hybrids (see eq. 6). Consistent with this inference, there is independent evidence of parental load and F1 hybrid vigor in both species pairs (*Populus*: Caseys et al. 2015; Christe et al. 2017; *Senecio*: Abbott and Brennan 2014).

## Discussion

In this article, we have used Fisher's geometric model to develop predictions for the relative fitness of any class of hybrid. The modeling approach is simple, with few free parameters, and it generates a wide range of testable predictions. We have tested some of these predictions with new and published datasets (Table 1), and the major predictions of the model are well supported.

We emphasize that our approach is designed for coarse‐grained patterns in the data, and typical outcomes of hybridization, without considering the particular set of substitutions that differentiate the parental lines, or the particular combination of alleles in an individual hybrid. The limitations of such an approach are seen in the low *r* ^2^ values associated with our model fitting (Table S5). Nevertheless, our approach should enable novel and complementary uses of genomic datasets, which do not depend on identifying individual loci with anomalous effects. In this approach, all alleles, and not just a handful of “speciation genes” might contribute to hybrid fitness.

A second goal of the present work was to show how Fisher's model can interpolate between previous modeling approaches, namely the classical theory of inbreeding (Wright 1922; Crow 1952), and models of genetic incompatibilities, each involving a small number of loci (Dobzhansky 1937; Orr 1995; Gavrilets 2004; Welch 2004). We have also shown that Fisher's model can account for empirical patterns that each approach has struggled to explain (Wright 1977; Moehring 2011; Moran et al. 2017).

Though we have stressed their similarities, we should also stress that Fisher's model and the model of incompatibilities remain different in two ways. First, as shown in Appendix 2, the two models agree only when the dominance relations of incompatibilities take a particular set of values (eq. A36)—albeit a biologically realistic set (Appendix 2; Turelli and Orr 2000). Second, even when predictions are identical for the quantity *f* (eq. 2), the two approaches still make different predictions for other kinds of data, which were not considered in the present work. The most important difference is the dependency of log fitness on *d*, the genomic divergence between the species. Under Fisher's geometric model, the log fitness of hybrids declines with $-d^{\alpha /2}$ (eqs. 1–2 and 5–6). By contrast, with the simplest models of incompatibilities, log fitness declines with $-d^{\ell \alpha /2}$ where ℓ is the number of loci involved in each incompatibility (Orr 1995; Welch 2004; Appendix 2, eq. A22). This is due to a snowball effect, where the number of incompatibilities grows explosively with $d^{\ell }$. This is a genuine difference between the modeling approaches, although truly discriminatory tests may be difficult. For example, it may not always be possible to distinguish between a snowballing model with a low value of α (equivalent to strong positive epistasis between incompatibilities), or a model where α is higher, but where the number of “incompatibilities” does not snowball, because they appear and disappear as the genetic background changes (Welch 2004; Fraïsse et al. 2016b; Guerrero et al. 2017; Kalirad and Azevedo 2017). Furthermore, Fisher's model can generate an “apparent snowball effect,” when introgressed segments contain multiple divergent sites (Fraïsse et al. 2016b), and this is true of the strongest empirical demonstrations of the effect (Matute et al. 2010; Moyle and Nakazato 2010; Wang et al. 2015).

Finally, given the simplicity and flexibility of the modeling approach explored here, and its predictive successes with a range of data, it should be readily extendable to address other outstanding questions in the study of hybridization. These include the putative role of hybridization in adaptive evolution (e.g., Mendez et al. 2012; Fraïsse et al. 2016b, 2016a; Duranton et al. 2017), the effects of recombination in shaping patterns of divergence (Aeschbacher et al. 2017; Schumer et al. 2018), the analysis of clines (Barton and Gale 1993; Macholán et al. 2011; Baird et al. 2012), and the roles of intrinsic versus extrinsic isolation (Chevin et al. 2014, Fig. S4). Given its ability to interpolate between models of different and extreme kinds, it should also be particularly useful for understanding hybridization in intermediate regimes, where parental genomes are characterized by both maladaptation and allelic coadaptation, or where the architecture of isolation involves many genes of small or moderate effect. Data—including those analyzed here—suggest that such architectures might be quite common (Davis and Wu 1996; Maside and Naveira 1996; Rosas et al. 2010; Morán and Fontdevila 2014; Baird 2017; Buerkle 2017; Boyle et al. 2017).

## Methods

### *Mytilus* DATA

Conserved tissues from the mussel species, *Mytilus edulis* and *Mytilus galloprovincialis*, and their hybrids, were retained from the work of Bierne et al. (2002, 2006). As reported in those studies, *M. edulis* from the North of France were crossed with *M. galloprovincialis* from the French Mediterranean coast to produce F1 hybrids (five males and one female; Bierne et al. 2002). The F1 were then used to produce an F2, and sex‐reciprocal backcrosses to *M. galloprovincialis* (which we denote here as BC_12_ and BC_21_; Bierne et al. 2006). In particular, oocytes from the F1 female were fertilized by the pooled sperm of the five F1 males producing F2 individuals, from which 132 individuals were sampled; oocytes from the F1 female were fertilized by pooled sperm of five *M. galloprovincialis* males to produce BC_12_, from which 72 individuals were sampled; and five *M. galloprovincialis* females were fertilized by pooled sperm from the five F1 males, producing BC_21_, from which 72 individuals were sampled. In addition to these hybrids, we also genotyped 129 individuals from “reference” populations of the two species, found in regions with relatively little contemporary introgression. In particular, we sampled *M. galloprovincialis* from Thau in the Mediterranean Sea; and sampled *M. edulis* from four locations in the North Sea and English Channel (The Netherlands, Saint‐Jouin, Villerville and Réville). Full details of these reference populations are found in Table S3.

In each case, gill tissues were conserved in ethanol at −20°C. DNA was extracted using a NucleaMag 96 Tissue kit (Macherey‐Nagel) and a KingFisher™ Flex (ThermoFisher Scientific). We then genotyped all samples for 98 *Mytilus* markers that were designed from the data of Fraïsse et al. (2016a). The flanking sequences of the 98 SNPs are provided in Table S7. Genotyping was subcontracted to LGC‐genomics and performed with the KASP™ chemistry (Kompetitive Allele Specific PCR, Semagn et al. 2014). Results are shown in Figure S6. Many of the 98 markers are not diagnostic for *M. edulis* and *M. galloprovincialis*, and so we retained only the 43 that were scored as heterozygous in all six of the F1 hybrids. To obtain a reduced set of strongly diagnostic markers, we measured sample allele frequencies in our pure species *M. edulis* and *M. galloprovincialis* samples (pooling *M. edulis* individuals across the four sampling locations; Table S3), and retained only markers for which the absolute difference in allele frequencies between species was > 90%. This yielded the set of 33 markers used for the right‐hand columns in Table 2. The “subsampled” data shown in the fourth column of Table 2, excluded any BC hybrid who carried the major allele typical of *M. edulis* in homozygous form. This yielded 56 BC hybrids. Because sequencing order was haphazard, to equalize the sample sizes, we retained the first 56 F2 to be sequenced.

### COLLATION OF PUBLISHED DATA

We searched the literature for published datasets combining measures of individual hybrid fitness, with genomic data that could be used to estimate *p*_12_ and *h*. We rejected any datasets where the measure of fecundity or fertility took an extreme low value for one of the pure species, suggesting that it is not a good proxy for fitness (e.g., Orgogozo et al. 2006), data where the fitness proxy correlated strongly with a measure of genetic abnormality such as aneuploidy (Xu and He 2011), or data where the states of many markers could not be unambiguously assigned, for example, due to shared variation. Before estimating the fitness surface, we also excluded any dataset where there was a highly significant rank correlation between the proportion of missing data in an individual, and either their heterozygosity, or fitness. For this reason, we did not proceed with reanalyses of the excellent datasets of Li et al. (2011), or Routtu et al. (2014) (see Table S2 for full details). For our reanalysis of the *Teleogryllus* data of Moran et al. (2017), we did not consider data from the second backcross, to avoid complications due to selection on heterozygosity during the first backcross, and because of an anomalous hatching rate in the *T. oceanicus* controls. For our reanalysis of the *Mus musculus* F2 (White et al. 2011), we used a conservative subset of these data; we excluded any individual where any X‐linked marker was scored as heterozygous (indicative of sequencing errors in heterogametic males; White et al. 2011), and controlled for variation in the uniparentally inherited markers, by retaining only individuals carrying *M. m. domesticus* mitochondria, and the *M. m. musculus* Y. However, results were little changed when we used all 304 individuals with fertility data (Table S6). Results were also unaffected when we used alternative proxies for fitness (Table S6). For the wild *Mus musculus* hybrids data (Turner and Harr 2014), fixed markers and their orientation between species were determined using previously published data of Staubach et al. (2012) and Yang et al. (2009). See details in the Dryad data package.

### ESTIMATION OF $g_{X}$ FROM ANNOTATED GENOMES

To fit the generalized linear models (GLM) to data from species with XY sex determination (Table 1), we needed to account for the different marker densities on the X and autosomes. Accordingly, we estimated the overall values of *p*_12_ and *h* from equation (16). We estimated the weightings, $g_{X}$ and $g_{A}$, from the total length of coding sequences associated with each chromosome type, ignoring the small contributions from the Y and mitochondria. In each case, we obtained the longest protein product for each unique gene, and then summed their lengths, using a custom R script. The $g_{X}$ values, shown in Table 1, were calculated as the total length of X‐linked CDS divided by the total CDS length. For *Mus musculus*, we used the reference genome assembly “GRCm38.p5.” For *Drosophila simulans*, we used the assembly “GCA_000754195.3 ASM75419v2,” and for *Drosophila yakuba* “GCA_000005975.1 dyak_caf1.” For *Drosophila pseudoobscura*, the current annotation was downloaded from FlyBase release 3.04 (Gramates et al. 2017). The .gtf file was then sorted and merged (combining overlapping coding sequences on each chromosome) using BEDTools (Quinlan and Hall 2010). Coding sequence lengths were calculated and summed over each chromosome, using custom awk commands.

### GLM METHODS

The linear model results shown in Table 3, Figure 5, Tables S5 and S6, and Figures S9–S11, were all fit in R v. 3.3.2 (R Core Team 2016). For datasets with quantitative fitness measures (White et al. 2011; Turner and Harr 2014; Fig. S10) we used the standard general linear models, with Gaussian errors, and chose data transformations to reduce heteroscedasticity. For binary fitness data (Noor et al. 2001; Christe et al. 2016; Chapman et al. 2016; Table 3; Fig. S9), we used binomial regression with a logit link implemented in the glm function; and with ordinal fitness data (Macdonald and Goldstein 1999; Moehring et al. 2006b; Fig. S11) we used proportional odds logistic regression (Agresti 2003), implemented in the *polr* function. In these cases, the *P*‐values shown in Table S6 were calculated by comparing the *t*‐value to the upper tail of normal distribution, as in a Wald test. For the non‐Gaussian models, we also report McFadden's pseudo‐*r* ^2^, defined as one minus the ratio of log likelihoods for the fitted and null models (McFadden 1974).

## DATA ACCESSIBILITY

The newly generated data on *Mytilus* and collated data are available as a Dryad package (doi:xxx).

Associate Editor: Z. Gompert

## Supporting information


**Figure S1**: A schematic representation of a phenotype evolving over time in two populations, labeled P1 and P2, starting from their most recent common ancestor (MRCA).
**Figure S2**: The breakdown associated with haploid hybrid genotypes, under simple models of phenotypic divergence.
**Figure S3**: The breakdown associated with haploid hybrid genotypes, under explicit population genetic simulations of phenotypic divergence.
**Figure S4**: The breakdown associated with haploid hybrid genotypes, under explicit population genetic simulations of phenotypic divergence, in scenarios involving discrete jumps in the optimal value for one of n = 2 traits.
**Figure S5**: The effects of an incompatibility on hybrid breakdown score, $s_{ijk}$ (eq. 41), when the incompatibility appears in a genotype comprising *i* loci that are homozygous for alleles from one parental species, *j* loci that are homozygous for alleles from the other parental species, and *k* loci that are heterozygous.
**Figure S6**: Genotype plot for the raw *Mytilus* data.
**Figure S7**: Plots of the *Drosophila* male backcross data reanalyzed here (see Table 1 and Moehring 2011).
**Figure S8**: Predictions of Fisher's geometric model for heterogametic male hybrids.
**Figure S9**: Estimation of the fitness surface for interspecific hybrids from plants (Table 1), namely wild hybrids of the genus Populus (row (a); Christe et al. 2016), and an F2 cross of the genus Senecio (row (b); Chapman et al. 2016).
**Figure S10**: Estimation of the fitness surface for subspecific hybrids from *Mus musculus* (Table 1, White et al. 2011; Turner and Harr 2014).
**Figure S11**: Estimation of the fitness surface for backcross male hybrids from *Drosophila* species pairs (Table 1, Macdonald and Goldstein 1999; Moehring et al. 2006a, b).
**Table S1**: The contribution of parental maladaptation to hybrid breakdown.
**Table S2**: Checks for appropriateness for genomic data sets.
**Table S3**: The reference populations for Mytilus crosses.
**Table S4**: Expected breakdown scores for homogametic female hybrids with paternal X silencing.
**Table S5**: Inferring the hybrid fitness surface from genomic data sets.
**Table S6**: The significance of individual regression coefficients.
**Table S7**: Information on the 98 markers used for the *Mytilus* genotyping.

## Appendix 1 The Brownian Bridge Approximation

In this Appendix, we derive the Brownian bridge approximation for the breakdown score of hybrid genotypes, under Fisher's geometric model. The Appendix is in three parts. First, we state our approximation without much justification. We will also assume haploid genomics, which makes the approximation simpler to explain, and also helps to draw connections with a previous model of epistasis introduced by Barton and Gale (1993). In the second section, we justify the approximation, by showing how it relates to explicit models of genetic and phenotypic divergence. We also show that the approximation is quite robust to varying assumptions about the divergence process. Third, and finally, we introduce the full, diploid version of the approximation, which is used for the results in the main text.

### Results with haploid genetics

In this section, and the following section, we will assume that the parental species P1 and P2 are haploid. In this case, of course, there is no heterozygosity ($p_{12}=0$) and the hybrid index is $h=p_{2}=1-p_{1}$.

We can now consider the *d* substitutions that differentiate P1 and P2 as a chain of vectors, in the *n*‐dimensional phenotypic space, which connect one of the parental phenotypes to the other. Our approximation is to treat this path, on each of the *n* traits, as describing a Brownian bridge, that is, a random walk constrained at its start and end points (Revuz and Yor 1999, Ch. 1 for details). This approximation will be justified by appealing to the random combinations of alleles that appear together in hybrid genotypes (see below).

To derive our approximation, let B(t) denote a Brownian bridge, taking place over a single unit of time, and with a rate $\sigma_{B}$. B(t) is normally distributed, with the following mean:

(A1) $$\begin{array}{cc} E(B(t)) & =(1-t)B(0)+tB(1),\;0\leq t\leq 1 \end{array}$$

where *B*(0) and *B*(1) are the fixed points at the beginning and end of the walk. The covariance at two time points is given by

(A2) $$\begin{array}{cc} \mathrm{Cov}(B\left(t_{1}\right),B\left(t_{2}\right)) & =\sigma_{B}^{2}\left(1-t_{2}\right)t_{1},\;0\leq t_{1}\leq t_{2}\leq 1 \end{array}$$

(Revuz and Yor 1999, Ch. 1). For our purposes, the start and end points of the walk are given by the trait values of the parental lines, so that, for trait *i*, $B\left(0\right)=z_{P1,i}$ and $B\left(1\right)=z_{P2,i}$. The total length of the walk represents the complete path of *d* substitutions, each with typical effect $v_{i}$, so that $\sigma_{B}^{2}=dv_{i}$. Finally, to determine the trait value for a hybrid carrying dh alleles that come from P2, we just “stop” the random walk at the intermediate “time” *h*. As such, using equations (A1) and (A2), the trait value $z_{i}$ will be a normal random variable with the following properties:

(A3) $$\begin{array}{ccc} z_{i} & ∼ & N(\mu_{i},\sigma_{i}^{2})\\ \mu_{i} & \equiv & E\left(z_{i}\right)=\left(1-h\right)z_{P1,i}+hz_{P2,i} \end{array}$$

(A4) $$\sigma_{i}^{2}\equiv \mathrm{Var}\left(z_{i}\right)=dv_{i}h\left(1-h\right)$$

The expectations and variances in these expressions involve two sorts of averaging. First, there is averaging over different realizations of evolutionary process, that is, the different sets of substitutions that might differentiate P1 and P2. Second, there is averaging over the subset of substitutions that might appear in a hybrid with a given value of *h*. Only this second averaging process will apply to real datasets, and it helps to explain the connection between our approximation and explicit models of divergence (see below).

To complete the results for haploidy, we note that the breakdown score, *S*, depends on the squared trait value (see eq. 4 of the main text). From normal theory, we then have:

(A5) $$E\left(z_{i}^{2}\right)=\sigma_{i}^{2}+\mu_{i}^{2}$$

(A6) Var zi2=2σi2σi2+2μi2

and so *S* will be approximately gamma distributed, with a mean and variance given by the weighted sum of these quantities (see also Rosas et al. 2010). The key quantity *f*, as defined in equation (2), is then found to be

(A7) $$\begin{array}{ccc} f\equiv \frac{E(S)}{E\left(S†\right)} & = & \frac{d\sum_{i}^{n}\lambda_{i}v_{i}h\left(1-h\right)+\sum_{i}^{n}\lambda_{i}{\left(\left(1-h\right)z_{P1,i}+hz_{P2,i}\right)}^{2}}{d\sum_{i}^{n}\lambda_{i}v_{i}}\\ & = & h\left(1-h\right)+f_{mal} \end{array}$$

where

(A8) $$\begin{array}{cc} f_{mal} & =\frac{\sum_{i}\lambda_{i}{\left(\left(1-h\right)z_{P1,i}+hz_{P2,i}\right)}^{2}}{d\sum_{i}\lambda_{i}v_{i}} \end{array}$$

The term $f_{mal}$ comes from the $\mu_{i}^{2}$ in equation (A5), and describes the contribution to hybrid breakdown from parental maladaptation. The value of $f_{mal}$ depends on the relative positions in phenotypic space of the two parental types. It is useful to consider a few simple cases, which are summarized in Table S1, and which we also use in the main text. First, let us consider the case where maladaptation accrues independently in the two parental lines. In this case, shown in Table S1b, we expect no covariance between their phenotypic deviations, and so $f_{mal}$ is just a weighted sum of the relative breakdown scores for the two parental lines

(A9) $$f_{mal}={\left(1-h\right)}^{2}f_{P1}+h^{2}f_{P2},\;\bar{z_{P1,i}z_{P2,i}}=0$$

where $f_{P1}=\sum_{i}\lambda_{i}z_{P1}^{2}/\sum_{i}\lambda_{i}dv_{i}$. The assumption of independent maladaptation should apply to many real datasets, and so equation (A9) is used in the main text, as the basis of equations (6)–(9) (see below for details). Independent maladaptation need not apply in every case, however, and it might be the case that P1 and P2 are identically maladapted, perhaps because of a shared unsuitability to a lab environment. This case, however, turns out to be comparable to the case with no parental adaptation. As shown in Table S1c, when both parental phenotypes are suboptimal, but identical, all genotypes suffer an additional constant contribution to their maladaptation. Finally, we can imagine a case where the two populations tend to become maladapted in opposite directions, such that the midparental phenotype is optimal. Results for this case are shown in Table S1d, but it is probably the least realistic of the three scenarios.

When parental lines are well adapted, such that $f_{mal}\approx 0$, then our haploid model is closely connected to a simple model of epistasis introduced by Barton and Gale (1993). They provide results for cline shape and linkage disequilibrium under this model (Barton and Gale 1993; p. 18).

### Comparison to simple models of divergence

The approximation introduced above includes only two fixed points—the parental phenotypes—and ignores their common ancestor, and all of the intermediate steps in their divergence. Nevertheless, we can be fairly confident that all of these intermediates had reasonably high fitness. The reason it is possible to ignore these intermediate steps is that mutations fix in a particular order during divergence, but in hybrids, they appear in random combinations, due to recombination and segregation. This makes the Brownian bridge approximation robust to a wide range of different assumptions about population divergence.

In this section of the Appendix, we illustrate this point, and explore the robustness of the approximation. For simplicity, we continue to assume haploid genetics, and consider only one phenotypic trait (hence, we write $z_{i}=z$ throughout). We will also limit ourselves to models with a low mutation rate, such that fixations occur one at a time. Given this last assumption, we can order the *d* fixations that differentiate P1 and P2 in a single chain, starting at P1, and then going back in time to the most recent common ancestor, and then forwards in time again toward P2. This is illustrated in Figure S1. We will denote as z(j), the trait value in the *j*th step of this chain, such that *z*(0) is the trait value for P1, and z(d) is the trait value for P2. Each genotype will contain a different number of alleles from P2, and the hybrid index of phenotype z(j) is simply h=j/d. Finally, the phenotypic effect of the *j*th substitution in the chain is defined by

(A10) $$m_{j}\equiv z\left(j\right)-z\left(j-1\right)$$

Figure S1 illustrates these quantities.

Now, let us first consider the case where both populations remain well adapted, or close to the optimum, throughout the divergence process. We can then model the phenotype at step *j* as a normal random variable.

(A11) z(j)∼N(0,v/2)

From equations (A10) and (A11), it follows that the effects of neighboring substitutions in the chain will negatively covary, implying that maladapted populations will tend to fix mutations that return them closer to the optimum:

(A12) $$\mathrm{Cov}\left(m_{i},m_{j}\right)=\left\{\begin{array}{cc} v, & i=j\\ -v/2, & i=j\pm 1\\ 0, & \mathrm{otherwise} \end{array}\right.$$

Now let us consider the phenotype that contains dh alleles from P2, namely z(dh). This phenotype can be written as the sum of substitutional changes, starting from the initial P1 phenotype:

(A13) $$z\left(dh\right)=z\left(0\right)+\sum_{i=1}^{dh}m_{i}$$

We define the relative breakdown of this genotype as $f_{h}\equiv z^{2}\left(dh\right)/\left(dv\right)$, and the expected value of this quantity is

(A14) E(fh)≡E(z2(dh))dv=1dv Var (z(0))+2 Cov z(0),∑i=1dhmi+ Var ∑i=1dhmi=1dv Var (z(0))+2 Cov z(0),∑i=1dhmi+dh Var mi+2∑i<j≤dh Cov mi,mj=12d+h(2ρz(0),mi+1+(dh−1)ρmimj)

where $\rho_{z\left(0\right),m_{i}}$ is the average correlation between the initial P1 phenotype, and the effect of each substitution; and $\rho_{m_{i}m_{j}}$ is the average correlation between each distinct pair of substitutions. Let us first evaluate equation (A14) when the dh mutations are added to the hybrid in the same fixed order that they appear in the chain of fit intermediates (Fig. S1). In this case, from equation (A12), only the first of the dh substitutions will covary with the initial phenotype, and so we have $\rho_{z\left(0\right),m_{i}}=-1/\left(\sqrt{2}dh\right)$. Similarly, each substitution will only covary with its preceding and subsequent substitutions, such that $\rho_{m_{i}m_{j}}=-\frac{1}{2}\frac{2(dh-1)}{dh(dh-1)}=-1/\left(dh\right)$. As such, when mutations are added in this fixed order, equation (A14) yields:

(A15) $$E\left(f_{h}\right)=\frac{1}{2d}=E\left(f_{P}\right)$$

So the expected breakdown of all genotypes, regardless of their hybrid index, is equal to that of the parental lines. Equation (A15) reflects the fact that each of these “hybrid” genotypes is one of the fit intermediates, and all will be very fit compared to the worst class of hybrid, unless the divergence, *d*, is very small. This result can be contrasted with the result for real hybrids, where segregation and recombination will bring together collections of alleles that fixed at different times. If the dh substitutions in equation (A13) are chosen at random, without replacement, from the complete set of *d* substitutions, then the correlation terms in equation (A14) are both reduced by a factor *h*, reflecting the fact that consecutive substitutions in the chain will only appear together with probability *h*. With $\rho_{z\left(0\right),m_{i}}=-1/\left(\sqrt{2}d\right)$, and $\rho_{m_{i},m_{j}}=-1/d$, equation (A14) becomes

(A16) $$\begin{array}{cc} E(f_{h}) & =h\left(1-h\right)+E\left(f_{P}\right) \end{array}$$

Even without conditioning on the parental phenotypes, equation (A16) is now very close to the Brownian bridge approximation of equation (A7). This is illustrated in Figure S2a. In this simulation run, the diverging populations remained close to the optimum throughout (as indicated by the jagged black line). By contrast, hybrids, which contain a randomized collection of alleles, can be very unfit on average (as shown by the smoother black curves). Their expected deviation from the optimum is well described by the Brownian bridge approximation (see colored dotted lines, which show eq. A7). Because the maladaptation of the parental lines is small, the analytical predictions are little affected if we explicitly account for the observed maladaptation, by calculating $f_{mal}$ from equation (A8) (see red dotted lines in Fig. S2a), or if we ignore parental maladaptation altogether, by setting $f_{mal}$ to zero (see blue dotted lines in Fig. S2a).

The model discussed above assumed that the effects of fixed mutations were normally distributed (see eqs. A10 and A11 and Fig. S2a), but the effect sizes of real substitutions might show substantial deviations from normality. To investigate the effects of nonnormal effect sizes, Figure S2b shows results when divergence included a few substitutions of very large effect (simulated by drawing the trait values, z(i), from a Cauchy distribution). The results show that the Brownian bridge continues to give a very good fit. This is because the approximation follows from robust, central‐limit‐type behavior, and so it does not depend strongly on the normality of the allelic effects.

The approximation is also robust if we assume more complicated patterns of divergence. For example, Figure S2c shows results where we have assumed that both populations adapted independently to a shifting optimum. This was modeled by generating the z(i) from an Ornstein–Uhlenbeck process, where the ancestral population (characterized by an intermediate value of *h*), was strongly maladapted.

All of the examples above assume well‐adapted parental lines. By contrast, under strong inbreeding, or mutation accumulation, divergence might be entirely maladaptive, with no tendency for populations to revert to the optimum. To model this, we can generate the allelic effects, $m_{i}$, as uncorrelated normal variables, starting from an optimal ancestral state. In this case, if we assume that the parental lines each contain d/2 substitutions, then their phenotypes are distributed as z(0),z(d)∼N(0,vd/2), such that $E(f_{P})=1/2$. As shown in Figure S2d, the Brownian bridge approximation remains accurate in this case. For a single trait, the accuracy does depend strongly on knowing the true parental trait values (dotted red lines), and the approximation that uses their expected squared values (dotted blue lines) can be quite inaccurate. Nevertheless, over multiple traits, we can expect the errors of this approximation to cancel out. A clear feature of this approximation, is that the expected levels of breakdown are constant for all haploid hybrids (the blue line in Fig. S2d is flat). This reflects the fact that, without natural selection, both the parental lines, and all possible hybrid genotypes are simply random assemblies of alleles, without any coadaptation. This echoes the result for diploids with maximal parental maladaptation, where breakdown levels depend solely on heterozygosity, and not on the hybrid index (see eq. 8 and Fig. 2A).

Examples illustrated in Figure S2 use simple heuristic simulations, where the phenotypes z(j) were drawn from a known distribution. In Figures S3 and S4 we show results when these phenotypes were generated by explicit population genetic simulations, including multiple phenotypic traits, and the appearance, fixation, or loss of new mutations. The full simulation procedure is described in Fraïsse et al. (2016b), but all simulations assumed allopatric divergence between P1 and P2, haploid genomics, and a low mutation rate, such that fixation or loss events could be tracked one mutation at a time. In the simulations shown in Figure S3, we began with a core set of parameters (Fig. S3a), and then varied these one at a time. Specifically, we varied the curvature of the fitness function, α, with a core set of parameters (eq. 1; Fig. S3b), the size of the parental populations (Fig. S3c), the total number of traits, *n* (eq. 4; Fig. S3d), and the shape of the distribution of mutant effects (Fig. S3e). These different parameter regimes led to very different levels of genetic divergence between the parents (i.e., different values of *d*), and different distributions of fixed phenotypic effects. Nevertheless, the Brownian bridge approximation remained very accurate in all cases. Finally, we moved the phenotypic optima during the simulation, so that much of the divergence took place by positive selection (see Fraïsse et al. 2016b; Fig. S3f). This sometimes led to an increase in the variance in breakdown score (Fig. S3f), but otherwise, the Brownian bridge approximation remained accurate.

Figure S4 shows further simulations, where the optima can move in discrete jumps (as in Orr 1998; Barton 2001). For n=2 traits, we compare divergence with a stable, fixed optimum (Fig. S4a), optima that vary identically for the two diverging populations (Fig. S4b), and   optima that move in different directions in the environments experienced by P1 and P2 (Fig. S4c). These scenarios correspond, roughly, to mutation‐order speciation via drift (Fig. S4a) or selection (Fig. S4b), and to ecological speciation (Fig. S4c), where reproductive isolation evolves as a byproduct of adaptation to different environments (Mani and Clarke 1990; Coyne and Orr 2004; Gavrilets 2004; Chevin et al. 2014).

When the optima can vary, we can also distinguish between intrinsic reproductive isolation, which involves maladaptation in traits that are under identical selection in all environments, and extrinsic isolation, which involves maladaptation in traits whose optima vary (Chevin et al. 2014). To illustrate this, the right‐hand columns in Figure S4 compare the breakdown caused by selection on both traits, measured in the environment to which P1 has adapted, and the breakdown due to one trait only, corresponding to the vertical axis on the fitness landscapes, for which the optimum never varies. This shows how the approach can be used to model breakdown that is caused by the correlated effects of adaptation to distinct environments (due to pleiotropy or linkage), even when hybrid fitness is measured in a benign environment, where these divergent selection pressures do not apply.

### Extension to diploidy

In this final section of the Appendix, we extend the results above to diploid genetics, as described in the main text. In this case, we must explicitly consider heterozygosity, *p*_12_, and the hybrid index must include contributions from both homozygous and heterozygous alleles: $h=p_{2}+p_{12}/2$.

To apply the Brownian bridge approximation to diploidy, we will assume that the chain of random vectors applies to alleles in their heterozygous state. Because all mutations are assumed to be additive on the phenotype, this means that the random walk, which begins at the phenotype of P1, will now end at the midparental phenotype, instead of the phenotype of P2. It also means that, to deal with alleles that appear in homozygous state, some sections of the walk must be counted twice. In particular, we need to study the following quantity:

(A17) $$B_{dip}\left(t_{1},t_{2}\right)\equiv B\left(t_{1}+t_{2}\right)+B\left(t_{1}\right)-B\left(0\right)$$

where B(·) is a Brownian bridge, as described in equations (A1) and (A2) above. From these equations, we find:

(A18) $$E\left(B_{dip}\left(t_{1},t_{2}\right)\right)=\left(1-2t_{1}-t_{2}\right)B\left(0\right)+\left(2t_{1}-t_{2}\right)B\left(1\right)$$

(A19) Var (Bdip(t1,t2))= Var (B(t1+t2))+ Var (B(t1))+2 Cov (B(t1),B(t1+t2))=σB2{(t1+t2)(1−t1−t2)+t1(1−t1)+2(1−t1−t2)t1}=σB2{t2(1−t2)+4t1(1−t1−t2)}

For our diploid problem, we start the walk at the P1 phenotype: $B\left(0\right)=z_{P\text{1},i}$, but end it at the midparent $B\left(1\right)=\left(z_{P\text{1},i}+z_{P2,i}\right)/2$. The total walk length is $\sigma_{B}^{2}=d\;v_{i}$, just as with haploidy, but now $v_{i}$ is understood as the typical effect of an allele in heterozygous state. Finally, we take the intermediate “times” as $t_{1}=p_{2}$ (this section of the walk is counted twice, to account for homozygous alleles), and $t_{2}=p_{12}$ (this section is counted once, to account for heterozygous alleles). This results in

(A20) $$\begin{array}{ccc} z_{i} & ∼ & N(\mu_{i},\sigma_{i}^{2})\\ \mu_{i} & \equiv & E\left(z_{i}\right)=\left(1-2p_{2}-p_{12}\right)z_{P1,i}+\left(2p_{2}+p_{12}\right)\frac{z_{P\text{1},i}+z_{P2,i}}{2}\\ & = & \left(1-h\right)z_{P1,i}+hz_{P2,i} \end{array}$$

(A21) σi2≡ Var zi=dvip12(1−p12)+4p1p2=dvi4h(1−h)−p12

We note that equation (A20) is identical to the haploid result of equation (A3). This shows that, once the hybrid index is correctly defined, all of the results concerning parental maladaptation (as shown in eqs. A8 and A9 and Table S1) apply equally to diploids and haploids. Combining equations (A20)–(A21) with equations (A5)–(A6), and equation (A9), leads directly to the diploid results shown in the main text.

The only other difference to note is that maladaptive diploid substitutions now contribute a possible $2^{2}v_{i}=4v_{i}$ to the trait variance. This implies that highly maladapted parents, who have fixed ∼d/2 such mutations, will have a breakdown score of $E\left(S_{P}\right)∼\frac{d}{2}4\sum_{i}^{n}\lambda_{i}v_{i}$, such that $E(f_{P})=2$ under diploidy (as opposed to $E(f_{P})=1/2$ under haploidy). The diploid result is used in the main text, to explore data from highly inbred lines of *Zea mays*.

## Appendix 2 Fisher's Model and Dobzhansky‐Muller Incompatibilities

In this Appendix, we show that a major prediction of Fisher's geometric model, assuming well‐adapted parents (eq. 9), can also be derived from a model of genetic incompatibilities, each involving a small number of loci. Such models stem from the classic work of Dobzhansky 1937 and have been important in the study of speciation genetics (Fraïsse et al. 2014; Orr 1995; Turelli and Orr 2000; Gavrilets 2004; Welch 2004; Demuth and Wade 2005; Fraïsse et al. 2016b). We begin by introducing the model in general form, and then explore different ways of assigning the parameters, which determine the contribution of individual incompatibilities to the overall level of breakdown.

### A general model of incompatibilities

Following Orr (1995), let us assume that certain combinations of alleles, at ℓ≤d of the divergent loci, can be intrinsically incompatible, while all other combinations confer high fitness. By assumption, the pure species genotypes, and their ancestral states, must be fit, but all other combinations have a fixed probability $\varepsilon_{\ell }$ of being incompatible.

The worst class of hybrid will contain all of the possible incompatibilities, and so its expected breakdown score will be proportional to the expected number of incompatibilities. This was calculated by Welch (2004, eqs. 1–2):

(A22) $$E\left(S_{†,I}\right)\propto \varepsilon_{\ell }\left(\begin{array}{c} d\\ \ell \end{array}\right)\left(2^{\ell }-\ell -1\right)$$

Here, and below, we use the subscript *I* to indicate a model of incompatibilities. To derive $f_{I}$ (eq. 2), we note that hybrids will have higher fitness when some of the incompatibilities are absent from their genomes (Turelli and Orr 2000). The probability that an incompatibility is present depends on how many of the ℓ loci are heterozygous. For a genotype comprising *i* loci that are homozygous for the P1 allele, *j* loci homozygous for the P2 allele, and *k* loci that are heterozygous, the probability required is:

(A23) πijk=2k−u(i)−u(j)2ℓ−2, with u(x)=0 if x>01ifx=0

which is the proportion of the possible combinations of heterospecific alleles that are present in an “ijk” genotype.

Incompatibilities may also have reduced effects due to recessivity, when their negative effects are masked by the presence of alternative, compatible alleles (Turelli and Orr 2000). To model this, we introduce the free parameter $s_{ijk}$, which is the expected increase in breakdown when an incompatibility appears in an ijk genotype. Finally, in a hybrid genome characterized by *p*_1_, *p*_2,_ and *p*_12_, the trinomial expansion of ${\left(p_{1}+p_{2}+p_{12}\right)}^{\ell }$, tells us how many ℓ‐locus genotypes of each type it is expected to contain. Putting these together, we have

(A24) $$f_{I}=\sum_{i+j+k=\ell }\left(\begin{array}{c} \ell \\ i,j,k \end{array}\right)p_{1}^{i}p_{2}^{j}p_{12}^{k}\pi_{ijk}s_{ijk}$$

where

$$\left(\begin{array}{c} \ell \\ i,j,k \end{array}\right)\equiv \frac{\ell !}{i!j!k!}$$

Equations (A23) and (A24) extend results with ℓ=2 and ℓ=3 from Turelli and Orr (2000), and represent a general model of breakdown caused by incompatibilities. A notable feature of these equations is their large number of free parameters. Even with symmetry between P1 and P2 (such that $s_{ijk}=s_{jik}$), we will still require a total of ⌊ℓ(1+ℓ/4)⌋ different $s_{ijk}$ values to specify the model (i.e., three extra parameters for two‐locus incompatibilities, five parameters for three‐locus incompatibilities etc.). There is good empirical evidence for, at least, two‐, three‐, and four‐locus incompatibilities (Fraïsse et al. 2014), and so with the $s_{ijk}$ and the $\varepsilon_{\ell }$, the full model would depend on at least 17 free parameters. By contrast, equation (9), from Fisher's geometric model, has no free parameters. The incompatibility‐based model is therefore much more flexible, but also much more difficult to explore.

In the following section, we examine different ways of assigning the $s_{ijk}$. We will show that a particular set of values make the model identical to Fisher's geometric model (eq. 9). We will also follow Turelli and Orr (2000), and show that these values are biologically plausible, given well‐established empirical patterns, and especially Haldane's Rule (see Haldane 1922 and Appendix 4).

### The dominance relations of incompatibilities

Let us begin by assigning the following functional form for the $s_{ijk}$:

(A25) $$s_{ijk}\propto \;{\left(\frac{1}{2}\right)}^{\delta k}$$

where below, we will use a constant of proportionality such that $s_{ijk}={\left(1/2\right)}^{\delta k}\cdot 2\left(2^{\ell }-2\right)$, to simplify the final results. In equation (A25), the parameter δ allows us to tune the dominance of incompatibilities, measured in terms of breakdown score, rather than fitness.

When δ=1, then each heterozygous locus halves the effects of incompatibility. This is equivalent to assuming that incompatibilities act multiplicatively, since each heterozygous locus halves the number of times that the incompatible combination of alleles is present in the genome. The $s_{ijk}$ under multiplicative selection (δ=1) are illustrated by the green points in Figure S5.

To determine the predictions of this model, let us substitute equation (A25) into equation (A24), and set δ=1. After some algebra, we find:

(A26) $$\begin{array}{cc} f_{I} & =2\left[1-{\left(p_{2}+\frac{1}{2}p_{12}\right)}^{\ell }-{\left(p_{1}+\frac{1}{2}p_{12}\right)}^{\ell }\right],\;\delta =1 \end{array}$$

(A27) $$\begin{array}{cc} & \equiv 2\left[1-h^{\ell }-{\left(1-h\right)}^{\ell }\right],\;\delta =1 \end{array}$$

(A28) $$\begin{array}{cc} & =4h(1-h),\;\ell =2,\;\delta =1 \end{array}$$

where *h* is the hybrid index (eq. 3). As such, when incompatibilities act multiplicatively, breakdown will depend solely on the hybrid index, and not on the heterozygosity. When pairwise incompatibilities are considered (eq. A28), the model becomes equivalent to the haploid version of Fisher's geometric model (see eq. A7), and also to the model of Barton and Gale (1993).

More importantly, equation (A28) predicts that breakdown should not change between the F1 and F2 crosses (both of which have $h=\frac{1}{2}$), and that homogametic F1 will have the highest possible breakdown score. As such, this multiplicative model cannot predict hybrid breakdown between the F1 and F2, nor Haldane's Rule, and both patterns have widespread empirical support (see Table A1 of Fraïsse et al. 2016b).

Now let us consider another extreme assumption. We assume that incompatibilities are fully recessive, such that no breakdown appears unless all incompatible alleles appear in homozygous or hemizygous form. We model this by making δ very large, such that $s_{ijk}=0$ unless k=0. These values are illustrated by the red points in Figure S5. With the assumption of complete recessivity, we find:

(A29) $$f_{I}=2\left[{\left(p_{1}+p_{2}\right)}^{\ell }-p_{1}^{\ell }-p_{2}^{\ell }\right],\;\;\delta \to \infty$$

(A30) $$=4h\left(1-h\right)-2p_{12}+p_{12}^{2},\;\;\ell =2,\;\delta \to \infty$$

Equation (A29) also fails to predict Haldane's Rule, unless there is substantial uniparental inheritance from both the male and female parents (see Appendix 4). This is because $f_{I}=0$ if $p_{1}p_{2}=0$, and so both male and female F1 will have identical and optimal fitness. For similar reasons, equation (A29) predicts that the fitness of heterogametic backcrosses will decrease with $p_{12,A}$, and this prediction is not supported by the relevant data (Moehring 2011).

We have shown that both extreme regimes (no recessivity, and complete recessivity) yield unsupported predictions. But what values of δ are biologically plausible? To answer this question, let us consider Haldane's Rule under an incompatibility‐based model, and ignoring uniparental inheritance. Assuming that males are heterogametic, Haldane's Rule will hold when

(A31) $$f_{I,F1♂}>f_{I,F1♀}$$

(see Appendix 4). If we use $g_{X}$ and $1-g_{X}$ to denote the proportions of the parental divergence that is found on the X and autosomes (as in eq. 16 of the main text), then using equations (A24) and (A25), equation (A31) is found to be equivalent to:

(A32) $${\left(2\left(1-g_{X}\right)+2^{\delta }g_{X}\right)}^{\ell }-{\left(1-g_{X}\right)}^{\ell }-{\left(1-g_{X}+2^{\delta }g_{X}\right)}^{\ell }>2^{\ell }-2$$

This condition is most difficult to satisfy when incompatibilities involve two loci (ℓ=2), and in this case, we find the solution:

(A33) $$\delta >\ln\left(\frac{2-g_{X}}{1-g_{X}}\right)/\ln\left(2\right).$$

The value of δ that is required to yield Haldane's Rule will therefore increase with $g_{X}$. Toward the limit of the biologically plausible range, when two‐thirds of the between‐species divergence is X‐linked ($g_{X}=2/3$) Haldane's Rule will hold only if δ>2. As such, setting δ=2, such that each heterozygous locus reduces the breakdown score by a factor of four, will yield Haldane's Rule in most cases. The $s_{ijk}$ values from equation (A25) with δ=2 are shown as yellow points in Figure S5. Another feature of the model with δ=2 is that it produces parameter dependencies that are very close to those predicted by Fisher's geometrical model (see also Manna et al. 2011). The similarity is clearest with two‐locus incompatibilities, where we find

(A34) $$f_{I}={\left(\frac{1}{2}\right)}^{\delta -2}p_{12}\left(1-p_{12}\left[1-{\left(\frac{1}{2}\right)}^{\delta }\right]\right)+4p_{1}p_{2},\;\;\ell =2$$

(A35) $$=4h\left(1-h\right)-p_{12}+\frac{1}{4}p_{12}^{2},\;\;\ell =2,\delta =2$$

Comparing equation (A35) to equation (9), shows that $f_{I}\approx f$ when we use equation (A25) with δ=2.

We can then go further, and find a set of $s_{ijk}$ values that yield exactly the same dependencies as Fisher's model. To do this, we set $f_{I}=f$, using equations (9) and (A24), and then solve for the $s_{ijk}$. After some algebra, we find

(A36) $$s_{ijk}=\frac{\left(\ell -k\right)\ell -{\left(i-j\right)}^{2}}{\ell \left(\ell -1\right)\pi_{ijk}}$$

These values are shown as blue points in Figure S5. Equation (A36) looks unwieldy, and it was derived solely to make the models agree. Nevertheless, it embodies biologically plausible assumptions about incompatibilities. First, the similarities between the blue and yellow points in Figure S5 show that Fisher's model corresponds to the assumption of partial recessivity, at levels sufficient to produce Haldane's Rule. Second, equation (A36) states that incompatibilities will have stronger effects when alleles from both parental species appear in homozygous state. For example, if the three alleles ABc form an incompatibility (with upper and lower case letters distinguishing alleles from P1 and P2), then equation (A36) predicts that the genotype Aa/BB/cc (with ijk=111) will tend to have lower fitness than the genotype AA/BB/Cc (with ijk=201) even though both genotypes contain the incompatibility, and both comprise two homozygous loci and one heterozygous locus.

This section has shown that Fisher's geometric model implies assumptions about “incompatibilities,” that yield well supported predictions. However, it remains true that the full incompatibility‐based model (eqs. A22–A24) is much more flexible. While that model is very parameter rich, the results above also suggest a family of models that might combine the flexibility and simplicity. When we assume only pairwise incompatibilities (ℓ=2), then equations (A28), (A30), (A35), and the result from Fisher's model (eq. 9), are all special cases of

(A37) f=4h(1−h)+(2f het −r−2)p12+(1+r−f het )p122

where *f*_het_ is the breakdown experienced by the global heterozygote (the genotype with $p_{12}=1$), and *r* is the rate of change in breakdown with *p*_12_.

## Appendix 3 Segregation and Recombination

In this Appendix, we consider the effects of segregation and recombination on the expected levels of breakdown. For a recombinant cross, such as the F2 or backcross, *p*_12_ and *h* might vary between individuals, and so we must treat *f* as a random variable. To see this, let us write equation (9) as

(A38) $$\begin{array}{ccc} f & = & 4h\left(1-h\right)-p_{12}\\ & = & p_{12}\left(1-p_{12}\right)+4p_{1}p_{2}\\ & = & p_{12}-p_{12}^{2}+4p_{1}-4p_{1}^{2}-4p_{1}p_{12} \end{array}$$

and so its expected value is:

(A39) $$\begin{array}{ccc} E(f) & = & {\bar{p}}_{12}\left(1-{\bar{p}}_{12}\right)+4{\bar{p}}_{1}{\bar{p}}_{2}-\mathrm{Var}\left(p_{12}\right)-4(\mathrm{Var}\left(p_{1}\right)\\ & & +\;\mathrm{Cov}(p_{12},p_{1})) \end{array}$$

where overbars represent expected values. The variances and covariances in equation (A39) will depend on the distribution of the divergence across the genome, and on patterns of segregation and recombination. However, we can derive simple and useful predictions if we assume that the divergence is equally distributed among *m* freely recombining regions. In this case, we can use results from a multinomial distribution.

(A40) $$\mathrm{Var}\left(p_{12}\right)=\frac{{\bar{p}}_{12}\left(1-{\bar{p}}_{12}\right)}{m}$$

(A41) $$\mathrm{Var}\left(p_{1}\right)=\frac{{\bar{p}}_{1}\left(1-{\bar{p}}_{1}\right)}{m}$$

(A42) $$\mathrm{Cov}\left(p_{12},p_{1}\right)=-\frac{{\bar{p}}_{1}{\bar{p}}_{12}}{m}$$

These results also apply to estimators from *m* independently segregating markers. As an example, let us compare the first backcross and the F2, in a population with strictly biparental inheritance. For the first backcross, we have ${\bar{p}}_{12}=\frac{1}{2}$ and $p_{1}p_{2}=0$, and so

(A43) E(f BC 1)=141−1m

For the F2, we have ${\bar{p}}_{12}=\frac{1}{2}$ and ${\bar{p}}_{1}={\bar{p}}_{2}=\frac{1}{4}$, and so

(A44) E(fF2)=121−1m=2E(f BC 1)

and so the predicted breakdown in the F2 is always double that of the first backcross. By the same method, we can also calculate the variance of *f*, but this requires higher order moments of the multinomial distribution (Newcomer et al. 2008), and this can yield lengthy expressions. However, the following results for the F2 are required to justify the approximation of equation (13) from the main text.

(A45) $$\begin{array}{ccc} E(4h(1-h)) & = & 1-\frac{1}{2m}\approx 1,\;\;{\bar{p}}_{12}=\frac{1}{2},\;{\bar{p}}_{1}=\frac{1}{4} \end{array}$$

(A46) $$\begin{array}{ccc} \mathrm{Var}(4h(1-h)) & = & \frac{2m-1}{4m^{3}}\approx \frac{1}{2m^{2}},\;\;{\bar{p}}_{12}=\frac{1}{2},\;{\bar{p}}_{1}=\frac{1}{4} \end{array}$$

Comparing equation (A46) to equation (A40) shows that most of the variance in F2 breakdown will be due to variation in heterozygosity.

## Appendix 4 Predictions of Fisher's Geometric Model with Sex‐Specific Inheritance

In this Appendix, we will develop some predictions of Fisher's geometric model, after relaxing the assumption of strictly biparental inheritance. These results justify claims in the main text, and also provide analytical support for some assertions in Fraïsse et al. (2016b).

### Additional notation and basics

As described in the main text, the basic strategy for incorporating sex‐specific inheritance is to write the genome‐wide measures as weighted sums of contributions from loci of different types. For example, if we have a system with an X chromosome and autosomes, and a subset of loci that are strictly maternally or paternally inherited, then we could write:

(A47) $$\begin{array}{ccc} p_{12} & = & g_{X}\;p_{12,X}+g_{A}\;p_{12,A}+g_{♀}\;p_{12,♀}+g_{♂}\;p_{12,♂}\\ p_{1} & = & g_{X}\;p_{1,X}+g_{A}\;p_{1,A}+g_{♀}\;p_{1,♀}+g_{♂}\;p_{1,♂}\\ p_{2} & = & g_{X}\;p_{2,X}+g_{A}\;p_{2,A}+g_{♀}\;p_{2,♀}+g_{♂}\;p_{2,♂}\\ h & = & g_{X}\;h_{X}+g_{A}\;h_{A}+g_{♀}\;h_{♀}+g_{♂}\;h_{♂} \end{array}$$

where $g_{X}+g_{A}+g_{♀}+g_{♂}=1$. The weightings will vary with the proportion of the divergence found at loci of each type, and with the typical effect sizes of the fixed differences. Following equation (3), the hybrid index for the X chromosome, can also be written as

(A48) $$h_{X}=p_{2,X}+\frac{p_{12,X}}{2}$$

and similarly for the other quantities. Here, as in the main text, we have assumed an XO system, such that females are homogametic, and males heterogametic. However, the equations are flexible enough to model a range of biological situations. For example, haplodiploidy might be modeled by setting $g_{X}=1$, and the uniparentally inherited loci (included via $g_{♀}$ and $g_{♂}$) might include the Y (or W) chromosome, organelles, selectively silenced regions of the X (or Z), or other imprinted loci (Turelli and Moyle 2007).

With sex‐specific inheritance, we also require assumptions about sex‐specific selection, and this can be incorporated in several ways (Connallon and Clark 2014; Fraïsse et al. 2016b). For example, sexual conflict can be modeled by assuming that there are differences in the optimal trait values for each sex (Connallon and Clark 2014). Alternatively, we could assume that some subset of the traits is under selection in only one sex, for example traits involved in spermatogenesis or oogenesis (Wu and Davis 1993; Coyne and Orr 2004). Finally, we could assume that sexes are under identical selection, which will usually require assumptions about dosage compensation (see below and Fraïsse et al. 2016b).

### Patterns in the F1: Haldane's rule and asymmetry

This section presents results for the initial F1 cross (P1 × P2), and particularly the findings of Haldane's Rule, and parental sex asymmetry (Haldane 1922; Turelli and Orr 2000; Turelli and Moyle 2007; Fraïsse et al. 2016b).

Haldane's Rule applies to offspring of different sexes, in species with chromosomal sex determination. It states that, when F1 breakdown is stronger in offspring of one sex, it will tend to be the heterogametic sex (Haldane 1922; Turelli and Orr 2000). Previous authors have noted that Fisher's model predicts this pattern (Barton 2001; Fraïsse et al. 2016b; Schiffman and Ralph 2017), and here, we extend this insight to give formal conditions for Haldane's Rule. We will assume identical selection in both sexes, and that pure‐species individuals of both sexes have the same fitness. This implies a form of dosage compensation, such that X‐linked alleles have identical effects in homozygous or hemizygous state (Mank et al. 2011; Fraïsse et al. 2016b). Assuming an XO or XY system, we expect greater breakdown in the heterogametic (male) sex on the condition that:

(A49) $$f_{F1♀}<f_{F1♂}.$$

In homogametic females, all divergent sites on the X and autosomes will be heterozygous, such that $p_{12}=g_{A}+g_{X}$, while by definition, uniparentally inherited loci will come from one parent alone, such that $p_{1}p_{2}=g_{♀}g_{♂}$. In heterogametic males, by contrast, only autosomal sites will be heterozygous, such that $p_{12}=g_{A}$, and X‐linked sites will be hemizygous and maternally inherited, such that $p_{1}p_{2}=\left(g_{♀}+g_{X}\right)g_{♂}$. Putting these results together with equation (6), and using

(A50) $$\Delta \equiv g_{♀}-g_{♂}$$

to denote the difference in the contributions of exclusively maternally and paternally inherited sites, we find:

(A51) $$f_{F1♀}=g_{♀}+g_{♂}-\Delta^{2}+f_{P♀}\frac{{\left(1+\Delta \right)}^{2}}{4}+f_{P♂}\frac{{\left(1-\Delta \right)}^{2}}{4}$$

(A52) $$=\frac{f_{P♀}+f_{P♂}}{4},\;g_{♀},g_{♂}=0$$

(A53) $$\begin{array}{ccc} f_{F1♂} & = & g_{♀}+g_{♂}+g_{X}-{\left(\Delta +g_{X}\right)}^{2}+f_{P♀}\frac{{\left(1+\Delta +g_{X}\right)}^{2}}{4}\\ & & +\;f_{P♂}\frac{{\left(1-\Delta -g_{X}\right)}^{2}}{4} \end{array}$$

(A54) $$\begin{array}{ccc} & = & g_{X}\left(1-g_{X}\right)+\left(1+g_{X}^{2}\right)\frac{f_{P♀}+f_{P♂}}{4}\\ & & +\;g_{X}\frac{f_{P♀}-f_{P♂}}{2},\;g_{♀},g_{♂}=0 \end{array}$$

where we have used $f_{P♀}$ and $f_{P♂}$ to denote the initial maladaptation of the maternal and paternal lines used to make the F1. We can now combine equations (A49)–(A54) to give formal conditions for Haldane's Rule. We find that the heterogametic sex will tend to show more breakdown if

(A55) $$0<g_{X}<g_{X}^{*}$$

where

(A56) $$g_{X}^{*}\equiv 2\frac{2-\Delta \left(4-\left(f_{P♀}+f_{P♂}\right)\right)+f_{P♀}-f_{P♂}}{4-(f_{P♀}+f_{P♂})}$$

(A57) $$=1-2\Delta ,\;f_{P♀},f_{P♂}=0$$

(A58) $$=2\frac{1-\Delta (2-f_{P})}{2-f_{P}},\;f_{P♀}=f_{P♂}\equiv f_{P}$$

(A59) $$=\frac{4-2f_{P♂}}{4-f_{P♂}},\;\Delta ,f_{P♀}=0$$

Several observations follow from the results above. First, the conditions for Haldane's Rule are always harder to satisfy as Δ increases, and so, from equation (A50), the presence of loci with exclusively maternal inheritance makes Haldane's Rule less likely, while paternal inheritance makes it more likely. Second, Haldane's Rule is usually more likely when the parental lines are maladapted. This is an effect of heterosis, that is an intrinsic advantage to heterozygosity, because the homogametic sex will tend to be more heterozygous. Third, the sole exception to this pattern is where the paternal line is much less fit than the maternal (i.e., if $f_{P♂}\gg f_{P♀}$ as in eq. A59). In this case, Haldane's Rule is less likely, because male F1 carry less of the unfit paternal genome.

The patterns above also clarify the broader role of uniparental effects in determining F1 fitness (Fraïsse et al. 2016b). For example, when there is no strictly uniparental inheritance, such that $g_{♀},g_{♂}=0$, then the homogametic F1 will always be fitter than the average of the parental lines (eq. A52). By contrast, when uniparental inheritance is present (eq. A51), then the relative breakdown remains roughly constant. This implies that the absolute breakdown score will decline steadily with *d*, the genetic divergence between the parental lines (see eqs. 2 and 5). As such, Fisher's model requires uniparental inheritance to explain the observation of an “F1 speciation clock” (Edmands 2002; Fraïsse et al. 2016b). This also helps to determine to the conditions for rare exceptions to Haldane's Rule (as observed, e.g., in *Teleogryllus* Moran et al. 2017). We predict such exceptions only when uniparentally inherited loci act on traits that are subject to selection only in the homogametic sex (Fraïsse et al. 2016b).

Uniparental inheritance is also required, by definition, to explain another widespread phenomenon: the strong asymmetry in fitness between the reciprocal F1, that is male–female versus female–male crosses of the same species pair (Turelli and Moyle 2007; Fraïsse et al. 2016b). Such asymmetry is found even in species without sex chromosomes, or even separate sexes, and so it cannot be connected to Haldane's Rule in any rigid sense (Bouchemousse et al. 2016; Fraïsse et al. 2016b). The results above (eqs. A51–A54) predict such asymmetry only in special circumstances: when there are uniparental effects of different sizes (Δ≠0), and parental lines with different levels of maladaptation ($f_{P♂}\neq f_{P♀}$). These are the assumptions that we used to analyze the *Teleogryllus* data of Moran et al. (2017) (see below). However, the observations of F1 asymmetry are very widespread, and apply even to species pairs that are both well adapted (Turelli and Moyle 2007; Fraïsse et al. 2016b). The reason for this apparent discrepancy is that the present work considers the expected breakdown, conditioned only on the levels of parental maladaptation ($f_{P♀}$ and $f_{P♂}$), and so expectations for the reciprocal F1 must be identical when $f_{P♂}=f_{P♀}$. If, by contrast, we condition on the phenotypic effects of uniparentally inherited alleles, then Fisher's geometric model can account for the asymmetries observed; this was demonstrated by Fraïsse et al. (2016b).

### Patterns in backcrosses

In this section, we provide further details of the analyses of backcross data. These include the male backcrosses from *Drosophila* (Noor et al. 2001; Macdonald and Goldstein 1999; Moehring et al. 2006a, 2006b), that were reanalyzed by Moehring (2011), and the female backcrosses from *Teleogryllus* presented by Moran et al. (2017). In both cases, the data come from hybrids of a single sex, and the fitness traits are components of fertility that are plausibly sex‐specific (i.e., spermatogenesis and oogenesis; see Table 1). As such, we treat the predictions of Fisher's geometric model as applying to a single sex.

The *Drosophila* data (Macdonald and Goldstein 1999; Noor et al. 2001; Moehring et al. 2006a, 2006b) come from three pairs of reciprocal backcrosses. The species pairs are *D. simulans/sechellia* (Macdonald and Goldstein 1999); *D. santomea/yakuba* (Moehring et al. 2006a, 2006b); and *D. pseudoobscura/persimilis* (Noor et al. 2001). In all three cases, the crosses can produce sterile hybrids, and so it is safe to assume that the parental lines are well adapted, compared to the worst possible class of hybrid, so that we can derive predictions from equation (9). Our analyses will also neglect loci with exclusively uniparental inheritance, because they do not qualitatively alter the predictions in this case. With these assumptions, as noted by Moehring (2011), hybrids can be characterized by two measures of “heterospecificity,” namely, the autosomal heterozygosity, $p_{12,A}$, and the hybrid index of the X, $h_{X}$. All datasets scored fertility in each hybrid fly as a binary, or ordinal trait (see Table 1), and so Moehring (2011) asked whether fertility problems varied systematically with $p_{12,A}$ and $h_{X}$. She found, in all six crosses, that backcross fertility problems correlated strongly and positively with $h_{X}$, but correlations with $p_{12,A}$ were weak and inconsistent. This is shown in Figure S7, and Table 3 of Moehring (2011). To show how Fisher's geometric model might account for these patterns, we can write equation (9) as

(A60) f BC ♂=4h(1−h)−p12=p12(1−p12)+4p2p1=a(1−a)+4x(1−x−a)=a−a2+4x−4x2−4ax

where

(A61) $$\begin{array}{ccc} a & \equiv & \left(1-g_{X}\right)p_{12,A}\\ x & \equiv & g_{X}h_{X} \end{array}$$

Figure S8a depicts the fitness surface of equation (A60) as a function of *x* and *a*. Each dataset of hybrid males could occupy a rectangular region of this surface, as determined by the its value of $g_{X}$, and this is how the data are plotted in Figure S7. The regions also correspond to a region of Figure 1 C, comprising the parallelogram delimited by the dotted horizontal line and the two arrows. From annotated *Drosophila* genomes, we estimated that $g_{X}=0.17$ might characterize the *simulans/sechellia* and *yakuba/santomea* pairs, and that $g_{X}=0.37$ might characterize the *pseudoobscura/persimilis* pair (Table 1; see Methods for details). The regions of parameter space for these values of $g_{X}$ are marked on Figure S8a, while panels b‐e show slices through the fitness surface for these values. In both cases, breakdown increases steadily with $h_{X}$, except in the improbable case that the recombinant autosomes were completely heterozygous (Fig. S8b–c). This is consistent with the positive correlations observed by Moehring (2011). By contrast, the dependencies on $p_{12,A}$ (Fig. S8d–e) vary in sign. This is consistent with the lack of consistent correlations with $p_{12,A}$ observed by Moehring (2011) (Fig. S7). Figure S8e also shows how, when $g_{X}$ is large, the correlations with $p_{12,A}$ reverse in sign, for extreme values of $h_{X}$. Figure S7e–f shows how the data of Noor et al. (2001) divide naturally into individuals with high, medium and low values of $h_{X}$, and together, this explains the rationale of the test presented in Table 3.

To analyze the female backcross data of Moran et al. (2017) for *Teleogryllus*, we used equation (10), which assumes that P1 is well adapted, while P2 is maladapted. We also assume that a proportion π of the paternal X chromosome is silenced. In this case, the quantities *p*_12_ and *h* are calculated without considering silenced alleles, because these make no contribution to the phenotype. This rich dataset contains a wide variety of cross types, and so the full predictions for all of the relevant hybrids are listed in Table S4.

## References

[evl366-bib-0001] Abbott, R. J. , and A. C. Brennan . 2014 Altitudinal gradients, plant hybrid zones and evolutionary novelty. Philos. Trans. R. Soc. B Biol. Sci. 369: 20130346. 10.1098/rstb.2013.0346 PMC4071520 24958920

[evl366-bib-0002] Abbott, R. J. , D. Albach , S. Ansell , J. W. Arntzen , S. J. E. Baird , N. Bierne , J. Boughman , A. Brelsford , C. A. Buerkle , R. Buggs , et al. 2013 Hybridization and speciation. J. Evol. Biol. 26: 229–246. 23323997 10.1111/j.1420-9101.2012.02599.x

[evl366-bib-0003] Aeschbacher, S. , J. P. Selby , J. H. Willis , and G. Coop . 2017 Population‐genomic inference of the strength and timing of selection against gene flow. Proc. Natl. Acad. Sci. 114: 7061–7066. 28634295 10.1073/pnas.1616755114 PMC5502586

[evl366-bib-0004] Agresti, A. 2003 Categorical data analysis. 2nd ed. John Wiley & Sons, Inc, Hoboken, New Jersey.

[evl366-bib-0005] Baird, S. J. E . 2017 The impact of high‐throughput sequencing technology on speciation research: maintaining perspective. J. Evol. Biol. 30: 1482–1487. 28786190 10.1111/jeb.13099

[evl366-bib-0006] Baird, S. J. E. , A. Ribas , M. Macholán , T. Albrecht , J. Piálek , and J. Goü de Bellocq . 2012 Where are the wormy mice? A reexamination of hybrid parasitism in the European house mouse hybrid zone: helminth parasites in the house mouse hybrid zone. Evolution 66: 2757–2772. 22946801 10.1111/j.1558-5646.2012.01633.x

[evl366-bib-0007] Barton, N. H . 2001 The role of hybridization in evolution. Mol. Ecol. 10: 551–568. 11298968 10.1046/j.1365-294x.2001.01216.x

[evl366-bib-0008] Barton, N. H. 2017 How does epistasis influence the response to selection? Heredity 118:96–109. 27901509 10.1038/hdy.2016.109 PMC5176114

[evl366-bib-0009] Barton, N. H. , and K. S. Gale . 1993 Genetic analysis of hybrid zones Pp. 13–45 *in* Hybrid zones and the evolutionary process, Harrison R. G. ed. Oxford Univ. Press, Oxford.

[evl366-bib-0010] Bierne, N. , F. Bonhomme , P. Boudry , M. Szulkin , and P. David . 2006 Fitness landscapes support the dominance theory of post‐zygotic isolation in the mussels *Mytilus edulis* and *M*. *galloprovincialis* . Proc. R. Soc. B 273: 1253–1260. 10.1098/rspb.2005.3440 PMC1560276 16720399

[evl366-bib-0011] Bierne, N. , P. David , P. Boudry , and F. Bonhomme . 2002 Assortative fertilization and selection at larval stage in the mussels *Mytilus* *edulis* and *M*. *galloprovincialis* . Evolution 56: 292–298. 11926497 10.1111/j.0014-3820.2002.tb01339.x

[evl366-bib-0012] Blanquart, F. , G. Achaz , T. Bataillon , and O. Tenaillon . 2014 Properties of selected mutations and genotypic landscapes under Fisher's geometric model. Evolution 68: 3537–3554. 25311558 10.1111/evo.12545 PMC4326662

[evl366-bib-0122] Bouchemousse, S. , L. Lévêque , G. Dubois , and F. Viard . 2016 Co‐Occurrence and Reproductive Synchrony Do Not Ensure Hybridization between an Alien Tunicate and Its Interfertile Native Congener. Evol. Ecol. 30: 69–87.

[evl366-bib-0013] Boyle, E. A. , Y. I. Li , and J. K. Pritchard . 2017 An expanded view of complex traits: from polygenic to omnigenic. Cell 169: 1177–1186. 28622505 10.1016/j.cell.2017.05.038 PMC5536862

[evl366-bib-0014] Buerkle, C. A . 2017 Inconvenient truths in population and speciation genetics point towards a future beyond allele frequencies. J. Evol. Biol. 30: 1498–1500. 28786192 10.1111/jeb.13106

[evl366-bib-0015] Butlin, R. K. , and M. G. Ritchie . 1989 Genetic coupling in mate recognition systems: what is the evidence? Biol. J. Linn. Soc. 37: 237–246.

[evl366-bib-0016] Caseys, C. , C. Stritt , G. Glauser , T. Blanchard , and C. Lexer . 2015 Effects of hybridization and evolutionary constraints on secondary metabolites: the genetic architecture of phenylpropanoids in European populus species. PLoS ONE 10: e0128200. 26010156 10.1371/journal.pone.0128200 PMC4444209

[evl366-bib-0017] Chapman, M. A. , S. J. Hiscock , and D. A. Filatov . 2016 The genomic bases of morphological divergence and reproductive isolation driven by ecological speciation in *Senecio* (Asteraceae). J. Evol. Biol. 29: 98–113. 26414668 10.1111/jeb.12765

[evl366-bib-0018] Chevin, L.‐M. , G. Decorzent , and T. Lenormand . 2014 Niche dimensionality and the genetics of ecological speciation. Evolution 68: 1244–1256. 24410181 10.1111/evo.12346

[evl366-bib-0019] Christe, C. , K. N. Stölting , M. Paris , C. Frasse , N. Bierne , and C. Lexer . 2017 Adaptive evolution and segregating load contribute to the genomic landscape of divergence in two tree species connected by episodic gene flow. Mol. Ecol. 26: 59–76. 27447453 10.1111/mec.13765

[evl366-bib-0020] Christe, C. , K. N. Stölting , L. Bresadola , B. Fussi , B. Heinze , D. Wegmann , and C. Lexer . 2016 Selection against recombinant hybrids maintains reproductive isolation in hybridizing *Populus* species despite F_1_ fertility and recurrent gene flow. Mol. Ecol. 25: 2482–2498. 26880192 10.1111/mec.13587

[evl366-bib-0119] Connallon, T. and A. G. Clark . 2014 Balancing Selection in Species with Separate Sexes: Insights from Fisher.s Geometric Model. Genetics 197: 991–1006. 24812306 10.1534/genetics.114.165605 PMC4096376

[evl366-bib-0021] Coyne, J. A. , and H. A. Orr . 1989 Two rules of speciation Pp. 180–207 *in* Otte D. and Endler J. A., eds. Speciation and its consequences. Sinauer Associates, Sunderland, MA.

[evl366-bib-0022] Coyne, J. A. , and H. A. Orr 2004 Speciation. Vol. 37. Sinauer Associates, Sunderland, MA.

[evl366-bib-0023] Crow, J. F. 1952 Dominance and overdominance *In* Heterosis, Gowen J. W. ed. Iowa State College Press, Ames, Iowa.

[evl366-bib-0024] Davis, A. W. , and C.‐I. Wu . 1996 The broom of the Sorcerer's apprentice: the fine structure of a chromosomal region causing reproductive isolation between two sibling species of *Drosophila* . Genetics 143: 1287–1298. 8807300 10.1093/genetics/143.3.1287 PMC1207397

[evl366-bib-0025] Demuth, J. P. , and M. J. Wade . 2005 On the theoretical and empirical framework for studying genetic interactions within and among species. Am. Nat. 165: 524–536. 15795850 10.1086/429276

[evl366-bib-0026] Dobzhansky, T. G. 1937 Genetics and the origin of species Columbia Univ. Press, New York.

[evl366-bib-0107] Duranton, M. , F. Allal , C. Fraïsse , N. Bierne , F. Bonhomme , and P.‐A. Gagnaire . 2018 The origin and remolding of genomic islands of differentiation in the European Sea Bass Nature Communications 9, no. 1. 10.1038/s41467-018-04963-6. PMC6023918 29955054

[evl366-bib-0027] Duranton, M. , F. Allal , C. Fraïsse , N. Bierne , F. Bonhomme , and P.‐A. Gagnaire . 2017 The origin and remolding of genomic islands of differentiation in the European sea bass. *bioRxiv* 10.1101/223750. PMC6023918 29955054

[evl366-bib-0121] Edmands, S. 2002 Does Parental Divergence Predict Reproductive Compatibility? Trends Ecol. Evol. 17: 520–527.

[evl366-bib-0028] Falush, D. , M. Stephens , and J. K. Pritchard . 2003 Inference of population structure using multilocus genotype data: linked loci and correlated allele frequencies. Genetics 164:1567–1587. 12930761 10.1093/genetics/164.4.1567 PMC1462648

[evl366-bib-0029] Fisher, R. A. 1930 The genetical theory of natural selection. Clarendon Press, Oxford.

[evl366-bib-0030] Fitzpatrick, B. M . 2008 Hybrid dysfunction: population genetic and quantitative genetic perspectives. Am. Nat. 171: 491–498. 20374137 10.1086/528991

[evl366-bib-0031] Fitzpatrick, B. M 2012 Estimating ancestry and heterozygosity of hybrids using molecular markers. BMC Evol. Biol. 12: 131. 22849298 10.1186/1471-2148-12-131 PMC3572440

[evl366-bib-0132] Fraïsse, C. , J. A. D. Elderfield , and J. J. Welch . 2014 The Genetics of Speciation: Are Complex Incompatibilities Easier to Evolve? J. Evol. Biol. 27: 688–699. 24581268 10.1111/jeb.12339

[evl366-bib-0032] Fraïsse, C. , K. Belkhir , J. J. Welch , and N. Bierne . 2016a Local interspecies introgression is the main cause of extreme levels of intraspecific differentiation in mussels. Mol. Ecol. 25:269–286. 26137909 10.1111/mec.13299

[evl366-bib-0033] Fraïsse, C. , P. A. Gunnarsson , D. Roze , N. Bierne , and J. J. Welch . 2016b The genetics of speciation: insights from Fisher's geometric model. Evolution 70:1450–1464. 27252049 10.1111/evo.12968

[evl366-bib-0034] Gavrilets, S. 2004 Fitness landscapes and the origin of species. Princeton Univ. Press, Princeton.

[evl366-bib-0035] Gompert, Z. , and C. A. Buerkle . 2010 Introgress: a software package for mapping components of isolation in hybrids. Mol. Ecol. Resour. 10: 378–384. 21565033 10.1111/j.1755-0998.2009.02733.x

[evl366-bib-0036] Gramates, L. S. , S. J. Marygold , G. dos Santos , J.‐M. Urbano , G. Antonazzo , B. B. Matthews , A. J. Rey , C. J. Tabone , M. A. Crosby , D. B. Emmert , et al. 2017 FlyBase at 25: looking to the future. Nucleic Acids Res. 45: D663–D671. 27799470 10.1093/nar/gkw1016 PMC5210523

[evl366-bib-0037] Guerrero, R. F. , C. D. Muir , S. Josway , and L. C. Moyle . 2017 Pervasive antagonistic interactions among hybrid incompatibility loci. PLoS Genet. 13: e1006817. 28604770 10.1371/journal.pgen.1006817 PMC5484531

[evl366-bib-0038] Haldane, J. B. S . 1922 Sex ratio and unisexual sterility in hybrid animals. J. Genet. 12: 101–109.

[evl366-bib-0039] Hallauer, A. R. , M. J. Carena , and J. B. Mirandad Filho . 2010 Quantitative genetics in maize breeding, Handbook of plant breeding. Vol. 6. Springer, Berlin.

[evl366-bib-0040] Hinze, L. L. , and K. R. Lamkey . 2003 Absence of epistasis for grain yield in elite maize hybrids. Crop Sci. 43: 46–56.

[evl366-bib-0041] Hogan, T. W. , and P. G. Fontana . 1973 Restoration of meiotic stability following artificial hybridisation and selection in *Teleogryllus* (Orth., Gryllidae). Bull. Entomol. Res. 62: 557–563.

[evl366-bib-0042] Hoy, R. , J. Hahn , and R. Paul . 1977 Hybrid cricket auditory behavior: evidence for genetic coupling in animal communication. Science 195: 82. 831260 10.1126/science.831260

[evl366-bib-0043] Hwang, S. , S.‐C. Park , and J. Krug . 2017 Genotypic complexity of Fisher's geometric model. Genetics 206: 1049–1079. 28450460 10.1534/genetics.116.199497 PMC5499163

[evl366-bib-0044] Kalirad, A. , and R. B. R. Azevedo . 2017 Spiraling complexity: a test of the snowball effect in a computational model of RNA folding. Genetics 206: 377–388. 28007889 10.1534/genetics.116.196030 PMC5419482

[evl366-bib-0046] Kiesselbach, T. (1922). Corn investigations. Neb. Agric. Exp. Stn. Bull. 20:5–151.

[evl366-bib-0045] Kiesselbach, T . 1930 The use of advanced generation hybrids as parents of double‐cross seed corn. J. Am. Soc. Agron. 22: 614–625.

[evl366-bib-0047] Kinman, M. L , and G. F. Sprague . 1945 Relation between number of parental lines and theoretical performance of synthetic varieties of corn. J. Am. Soc. Agron. 37: 341–351.

[evl366-bib-0048] Li, X. , X. Wang , Y. Wei , and E. C. Brummer . 2011 Prevalence of segregation distortion in diploid alfalfa and its implications for genetics and breeding applications. Theor. Appl. Genet. 123: 667–679. 21625992 10.1007/s00122-011-1617-5

[evl366-bib-0049] Lindstrom, E. W. 1941 Analysis of modern maize breeding principles and methods Pp. 667–679 *in* Proceedings of the 7th International Genetics Congress, Edinburgh, Scotland .

[evl366-bib-0050] Lindtke, D. , Z. Gompert , C. Lexer , and C. A. Buerkle . 2014 Unexpected ancestry of *Populus* seedlings from a hybrid zone implies a large role for postzygotic selection in the maintenance of species. Mol. Ecol. 23: 4316–4330. 24750473 10.1111/mec.12759

[evl366-bib-0051] Lindtke, D. , C. A. Buerkle , T. Barbará , B. Heinze , S. Castiglione , D. Bartha , and C. Lexer . 2012 Recombinant hybrids retain heterozygosity at many loci: new insights into the genomics of reproductive isolation in *Populus* . Mol. Ecol. 21: 5042–5058. 22989336 10.1111/j.1365-294X.2012.05744.x

[evl366-bib-0052] Lynch, M . 1991 The genetic interpretation of inbreeding depression and outbreeding depression. Evolution 45: 622–629. 28568822 10.1111/j.1558-5646.1991.tb04333.x

[evl366-bib-0053] Macdonald, S. J. , and D. B. Goldstein . 1999 A quantitative genetic analysis of male sexual traits distinguishing the sibling species *Drosophila* *simulans* and *D*. *sechellia* . Genetics 153: 1683–1699. 10581276 10.1093/genetics/153.4.1683 PMC1460840

[evl366-bib-0054] Macholán, M. , S. J. E. Baird , P. Dufková , P. Munclinger , B. V. Bímová , and J. Piálek . 2011 Assessing multilocus introgression patterns: a case study on the mouse X chromosome in central Europe: heterogeneity of introgression on the mouse X chromosome. Evolution 65: 1428–1446. 21521193 10.1111/j.1558-5646.2011.01228.x

[evl366-bib-0154] Mank, J. E. , D. J. Hosken , and N. Wedell . 2011 Some Inconvenient Truths about Sex Chromosome Dosage Compensation and the Potential Role of Sexual Conflict. Evolution 65: 2133–2144. 21790564 10.1111/j.1558-5646.2011.01316.x

[evl366-bib-0155] Manna, F. , G. Martin , and T. Lenormand . 2011 Fitness Landscapes: An Alternative Theory for the Dominance of Mutation. Genetics 189: 923–937. 21890744 10.1534/genetics.111.132944 PMC3213354

[evl366-bib-0055] Mani, G. S. , and B. C. Clarke . 1990 Mutational order: a major stochastic process in evolution. Proc. R. Soc. Lond. B Biol. Sci. 240: 29–37. 1972992 10.1098/rspb.1990.0025

[evl366-bib-0056] Martin, G . 2014 Fisher's geometrical model emerges as a property of complex integrated phenotypic networks. Genetics 197: 237–255. 24583582 10.1534/genetics.113.160325 PMC4012483

[evl366-bib-0057] Martin, G. , and T. Lenormand . 2006 A general multivariate extension of Fisher's geometrical model and the distribution of mutation fitness effects across species. Evolution 60: 893–907. 16817531

[evl366-bib-0058] Martin, J. M. , and A. R. Hallauer . 1976 Relation between heterozygosis and yield for four types of maize inbred lines. Egypt. J. Genet. Cytol. 5: 119–135.

[evl366-bib-0059] Maside, X. R. , and H. F. Naveira . 1996 On the difficulties of discriminating between major and minor hybrid male sterility factors in *Drosophila* by examining the segregation ratio of sterile and fertile sons in backcrossing experiments. Heredity 77: 433–438. 8885383 10.1038/hdy.1996.163

[evl366-bib-0060] Matute, D. R. , I. A. Butler , D. A. Turissini , and J. A. Coyne . 2010 A test of the snowball theory for the rate of evolution of hybrid incompatibilities. Science 329: 1518–1521. 20847270 10.1126/science.1193440

[evl366-bib-0061] McFadden, D. 1974 Conditional logit analysis of qualitative choice behavior Pp. 105–142 *in* Zarembka P. ed. Frontiers in econometrics. Academic Press, Cambridge, Massachusetts.

[evl366-bib-0062] Melchinger, A. E . 1987 Expectation of means and variances of testcrosses produced from F2 and backcross individuals and their selfed progenies. Heredity 59: 105–115.

[evl366-bib-0063] Mendez, F. L. , J. C. Watkins , and M. F. Hammer . 2012 A haplotype at STAT2 introgressed from neanderthals and serves as a candidate of positive selection in papua New Guinea. Am. J. Hum. Genet. 91: 265–274. 22883142 10.1016/j.ajhg.2012.06.015 PMC3415544

[evl366-bib-0064] Moehring, A. J. , A. Llopart , S. Elwyn , J. A. Coyne , and T. F. C. Mackay . 2006a The genetic basis of postzygotic reproductive isolation between *Drosophila santomea* and *D*. *yakuba* due to hybrid male sterility. Genetics 173: 225–233. 16510788 10.1534/genetics.105.052985 PMC1461443

[evl366-bib-0065] Moehring, A. J. , A. Llopart , S. Elwyn , J. A. Coyne , and T. F. C. Mackay 2006b The genetic basis of prezygotic reproductive isolation between *Drosophila santomea* and *D*. *yakuba* due to mating preference. Genetics 173: 215–223. 16510787 10.1534/genetics.105.052993 PMC1461457

[evl366-bib-0066] Moehring, A. J . 2011 Heterozygosity and its unexpected correlations with hybrid sterility. Evolution 65: 2621–2630. 21884060 10.1111/j.1558-5646.2011.01325.x PMC3166512

[evl366-bib-0067] Moll, R. H. , J. H. Lonnquist , J. V. Fortuno , and E. C. Johnson . 1965 The relationship of heterosis and genetic divergence in maize. Genetics 52: 139–144. 17248265 10.1093/genetics/52.1.139 PMC1210832

[evl366-bib-0068] Moran, P. A. , M. G. Ritchie , and N. W. Bailey . 2017 A rare exception to Haldanes rule: are X chromosomes key to hybrid incompatibilities? Heredity 118:554–562. 28098850 10.1038/hdy.2016.127 PMC5436020

[evl366-bib-0069] Morán, T. , and A. Fontdevila . 2014 Genome‐wide dissection of hybrid sterility in drosophila confirms a polygenic threshold architecture. J. Hered. 105: 381–396. 24489077 10.1093/jhered/esu003

[evl366-bib-0070] Moyle, L. C. , and T. Nakazato . 2010 Hybrid incompatibility “Snowballs” between solanum species. Science 329: 1521–1523. 20847271 10.1126/science.1193063

[evl366-bib-0071] Neal, N. P . 1935 Decrease in yielding capacity in advanced generations of hybrid corn. J. Am. Soc. Agron. 51: 666–670.

[evl366-bib-0170] Newcomer, J. T. , N. K. Neerchal , and J. G. Morel . 2008 Computation of Higher Order Moments from Two Multinomial Overdispersion Likelihood Models. Dep. Math. Stat. Univ. Md. Baltim. USA.

[evl366-bib-0072] Noor, M. A. F. , K. L. Grams , L. A. Bertucci , Y. Almendarez , J. Reiland , and K. R. Smith . 2001 The genetics of reproductive isolation and the potential for gene exchange between *Drosophila pseudoobscura* and *D*. *persimilis* via backcross hybrid males. Evolution 55: 512–521. 11327159 10.1554/0014-3820(2001)055[0512:tgoria]2.0.co;2

[evl366-bib-0073] Orgogozo, V. , K. W. Broman , and D. L. Stern . 2006 High‐resolution quantitative trait locus mapping reveals sign epistasis controlling ovariole number between two *Drosophila* species. Genetics 173: 197–205. 16489225 10.1534/genetics.105.054098 PMC1461429

[evl366-bib-0074] Orr, H. A . 1995 The population genetics of speciation: the evolution of hybrid incompatibilities. Genetics 139: 1805–1813. 7789779 10.1093/genetics/139.4.1805 PMC1206504

[evl366-bib-0075] Orr, H. A 1998 The population genetics of adaptation: the distribution of factors fixed during adaptive evolution. Evolution 52: 935–949. 28565213 10.1111/j.1558-5646.1998.tb01823.x

[evl366-bib-0076] Pollak, E. , H. F. Robinson , and R. E. Comstock . 1957 Inter‐population hybrids in open‐pollinated varieties of maize. Am. Nat. 91: 387–391.

[evl366-bib-0077] Quinlan, A. R. , and I. M. Hall . 2010 BEDTools: a flexible suite of utilities for comparing genomic features. Bioinformatics 26: 841–842. 20110278 10.1093/bioinformatics/btq033 PMC2832824

[evl366-bib-0078] R Core Team . 2016 R: a language and environment for statistical computing. R found. Stat. Comput. Vienna Austria https://www.R-project.org.

[evl366-bib-0079] Revuz, D. , and M. Yor . 1999 Continuous martingales and Brownian motion. 3rd ed. Springer‐Verlag, New York.

[evl366-bib-0080] Richey, F. D. , and G. F. Sprague . 1931 Experiments on hybrid vigor and convergent improvement in corn. 267. U.S. Department of Agriculture, Washington, D.C., pp. 1–2 2.

[evl366-bib-0081] Rockman, M. V . 2012 The QTN program and the alleles that matter for evolution: All that's gold does not glitter. Evolution 66: 1–17. 22220860 10.1111/j.1558-5646.2011.01486.x PMC3386609

[evl366-bib-0082] Rosas, U. , N. H. Barton , L. Copsey , P. Barbier de Reuille , and E. Coen . 2010 Cryptic variation between species and the basis of hybrid performance. PLoS Biol. 8: e1000429. 20652019 10.1371/journal.pbio.1000429 PMC2907293

[evl366-bib-0083] Routtu, J. , M. D. Hall , B. Albere , C. Beisel , R. Bergeron , A. Chaturvedi , J.‐H. Choi , J. Colbourne , L. De Meester , M. T. Stephens , et al. 2014 An SNP‐based second‐generation genetic map of Daphnia magna and its application to QTL analysis of phenotypic traits. BMC Genomics 15: 1033. 25431334 10.1186/1471-2164-15-1033 PMC4301878

[evl366-bib-0084] Roux, C. , C. Fraïsse , J. Romiguier , Y. Anciaux , N. Galtier , and N. Bierne . 2016 Shedding light on the grey zone of speciation along a continuum of genomic divergence. PLoS Biol. 14: e2000234. 28027292 10.1371/journal.pbio.2000234 PMC5189939

[evl366-bib-0085] Schiffman, J. S. , and P. L. Ralph . 2017 System drift and speciation. *bioRxiv* 10.1101/231209. PMC9292711 34529267

[evl366-bib-0086] Schumer, M. , C. Xu , D. L. Powell , A. Durvasula , L. Skov , C. Holland , J. C. Blazier , S. Sankararaman , P. Andolfatto , G. G. Rosenthal , et al. 2018 Natural selection interacts with recombination to shape the evolution of hybrid genomes. Science 360: 656–660. 29674434 10.1126/science.aar3684 PMC6069607

[evl366-bib-0087] Semagn, K. , R. Babu , S. Hearne , and M. Olsen . 2014 Single nucleotide polymorphism genotyping using Kompetitive Allele Specific PCR (KASP): overview of the technology and its application in crop improvement. Mol. Breed. 33: 1–14.

[evl366-bib-0088] Sentz, J. C. , H. F. Robinson , and R. E. Comstock . 1954 Relation between heterozygosis and performance in maize. Agron. J. 46: 514–520.

[evl366-bib-0089] Shehata, A. H. , and N. L. Dhawan . 1975 Genetic analysis of grain yield of maize as manifested in diverse varietal populations and their crosses. Egypt. J. Genet. Cytol. 4: 90–116.

[evl366-bib-0105] Staubach, F. , A. Lorenc , P. W. Messer , K. Tang , D. A. Petrov , and D. Tautz . 2012 Genome patterns of selection and introgression of haplotypes in natural populations of the house mouse (Mus Musculus). PLoS Genetics 8: e1002891. 10.1371/journal.pgen.1002891. PMC3431316 22956910

[evl366-bib-0090] Stringfield, G . 1950 Heterozygosis and hybrid vigor in maize. Agron. J. 42: 45–112.

[evl366-bib-0091] Tenaillon, O. , O. K. Silander , J.‐P. Uzan , and L. Chao . 2007 Quantifying organismal complexity using a population genetic approach. PLoS ONE 2: e217. 17299597 10.1371/journal.pone.0000217 PMC1790863

[evl366-bib-0092] Turelli, M. , and L. C. Moyle . 2007 Asymmetric postmating isolation: Darwin's corollary to haldane's rule. Genetics 176: 1059–1088. 17435235 10.1534/genetics.106.065979 PMC1894575

[evl366-bib-0093] Turelli, M. , and H. A. Orr . 2000 Dominance, epistasis and the genetics of postzygotic isolation. Genetics 154: 1663. 10747061 10.1093/genetics/154.4.1663 PMC1461023

[evl366-bib-0094] Turner, L. M. , and B. Harr . 2014 Genome‐wide mapping in a house mouse hybrid zone reveals hybrid sterility loci and Dobzhansky‐Muller interactions. Elife 3: e02504. 10.7554/eLife.02504 PMC4359376 25487987

[evl366-bib-0095] Wang, R. J. , M. A. White , and B. A. Payseur . 2015 The pace of hybrid incompatibility evolution in house mice. Genetics 201: 229–242. 26199234 10.1534/genetics.115.179499 PMC4566265

[evl366-bib-0096] Waser, N. M. 1993 Population structure, optimal outbreeding and assortative mating in angiosperms Pp. 173–199 *in* Thornhill N. W., ed. The natural history of inbreeding and outbreeding: Theoretical and empirical perspectives. Chicago Univ. Press, Chicago.

[evl366-bib-0097] Waxman, D. , and J. J. Welch . 2005 Fisher's microscope and haldane's ellipse. Am. Nat. 166: 447–457. 16224701 10.1086/444404

[evl366-bib-0098] Welch, J. J . 2004 Accumulating dobzhansky‐muller incompatibilities: reconciling theory and data. Evolution 58: 1145–1156. 15266965 10.1111/j.0014-3820.2004.tb01695.x

[evl366-bib-0099] Welch, J. J. , and D. Waxman . 2003 Modularity and the cost of complexity. Evolution 57: 1723–1734. 14503615 10.1111/j.0014-3820.2003.tb00581.x

[evl366-bib-0100] White, M. A. , B. Steffy , T. Wiltshire , and B. A. Payseur . 2011 Genetic dissection of a key reproductive barrier between nascent species of house mice. Genetics 189: 289–304. 21750261 10.1534/genetics.111.129171 PMC3176122

[evl366-bib-0101] Wright, S . 1922 The effects of inbreeding and crossbreeding on guinea pigs : III. crosses between highly inbred families. U. S. Dep. Agric. Bull. 1121.

[evl366-bib-0102] Wright, S. 1977 Inbreeding depression and heterosis: plants *In* Evolution and the genetics of populations, volume 3: Experimental results and evolutionary deductions, vol. 3. Chicago Univ. Press, Chicago.

[evl366-bib-0120] Wu, C.‐I. and A. W. Davis . 1993 Evolution of Postmating Reproductive Isolation: The Composite Nature of Haldane's Rule and Its Genetic Bases. Am. Nat. 142: 187–212. 19425975 10.1086/285534

[evl366-bib-0103] Xu, M. , and X. He . 2011 Genetic incompatibility dampens hybrid fertility more than hybrid viability: yeast as a case study. PLoS ONE 6:e18341. 10.1371/journal.pone.0018341 PMC3071822 21494679

[evl366-bib-0106] Yang, H. , Y. Ding , L. N. Hutchins , J. Szatkiewicz , T. A. Bell , B. J. Paigen , J. H. Graber , F. P.‐M. de Villena , and G. A. Churchill . 2009 A customized and versatile high‐density genotyping array for the mouse Nature Methods 6: 663–666. 10.1038/nmeth.1359 PMC2735580 19668205

