# Supplementary material for: Coadapted genomes and selection on hybrids: Fisher's geometric model explains a variety of empirical patterns
Source: Evol Lett. 2018 Aug 14;2(5):472–98. doi: 10.1002/evl3.66 (PMC6145440; doi:10.1002/evl3.66)
Supplement: Supplementary file 1 — Figure S1: A schematic representation of a phenotype evolving over time in two populations, labeled P1 and P2, starting from their most recent common ancestor (MRCA). Figure S2: The breakdown associated with haploid hybrid genotypes, under simple models of phenotypic divergence. Figure S3: The breakdown associated with haploid hybrid genotypes, under explicit population genetic simulations of phenotypic divergence. Figure S4: The breakdown associated with haploid hybrid genotypes, under explicit population genetic simulations of phenotypic divergence, in scenarios involving discrete jumps in the optimal value for one of n = 2 traits. Figure S5: The effects of an incompatibility on hybrid breakdown score, sijk (eq. 41), when the incompatibility appears in a genotype comprising i loci that are homozygous for alleles from one parental species, j loci that are homozygous for alleles from the other parental species, and k loci that are heterozygous. Figure S6: Genotype plot for the raw Mytilus data. Figure S7: Plots of the Drosophila male backcross data reanalyzed here (see Table 1 and Moehring 2011). Figure S8: Predictions of Fisher's geometric model for heterogametic male hybrids. Figure S9: Estimation of the fitness surface for interspecific hybrids from plants (Table 1), namely wild hybrids of the genus Populus (row (a); Christe et al. 2016), and an F2 cross of the genus Senecio (row (b); Chapman et al. 2016). Figure S10: Estimation of the fitness surface for subspecific hybrids from Mus musculus (Table 1, White et al. 2011; Turner and Harr 2014). Figure S11: Estimation of the fitness surface for backcross male hybrids from Drosophila species pairs (Table 1, Macdonald and Goldstein 1999; Moehring et al. 2006a, b). Table S1: The contribution of parental maladaptation to hybrid breakdown. Table S2: Checks for appropriateness for genomic data sets. Table S3: The reference populations for Mytilus crosses. Table S4: Expected breakdown scores for homogametic female hybrids [file EVL3-2-472-s001.pdf]

Supplementary material of "Coadapted genomes and selection on hybrids: Fisher's geometric model explains a variety of empirical patterns"

Alexis Simon[1,2,*], Nicolas Bierne[1], and John J. Welch[2]

1. Institut des Sciences de l'Évolution UMR5554, Université de Montpellier, CNRS-IRD-EPHE-UM, France.
2. Department of Genetics, University of Cambridge, Downing St. Cambridge, CB23EH, UK.

* Author for correspondence: alexis.simon@umontpellier.fr

List of Figures

S1 The phenotypic walk between P1 and P2 2
S2 Breakdown of haploid hybrids under simple simulations 3
S3 Breakdown of haploid hybrids under full population genetic simulations 4
S4 Breakdown of haploid hybrids under full population genetic simulations for discrete optimum jumps 5
S5 The effects of an incompatibility on hybrid breakdown score 6
S6 Genotype plot for the raw *Mytilus* data 7
S7 Plots of the *Drosophila* male backcross data reanalyzed 8
S8 Predictions of Fisher's geometric model for heterogametic male hybrids 9
S9 Estimation of the fitness surface for interspecific hybrids from plants 10
S10 Estimation of the fitness surface for subspecific hybrids from *Mus musculus* 11
S11 Estimation of the fitness surface for backcross male hybrids from *Drosophila* species pairs 12

List of Tables

S1 The contribution of parental maladaptation to hybrid breakdown 13
S2 Checks for appropriateness for genomic data sets 14
S3 The reference populations for *Mytilus* crosses 15
S4 Expected breakdown scores for homogametic female hybrids with paternal X silencing . 15
S5 Inferring the hybrid fitness surface from genomic data sets 16
S6 The significance of individual regression coefficients 17
S7 Information on the 98 markers used for the *Mytilus* genotyping 18

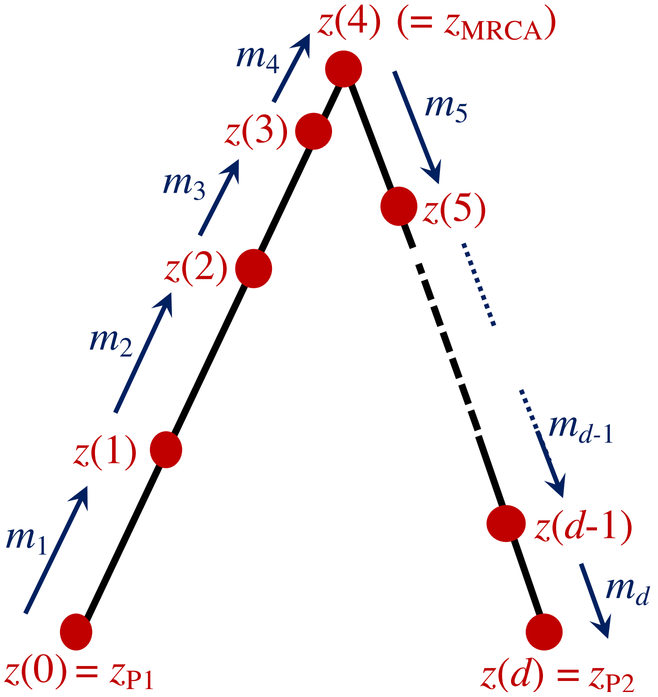

Figure S1: A schematic representation of a phenotype evolving over time in two populations, labeled P1 and P2, starting from their most recent common ancestor (MRCA). The populations differ by d substitutions. Each red dot represents the value of the phenotypic trait after each substitution, $z(i)$. The changes in phenotype, due to allelic substitutions, are denoted m_i (eq. 27), and are shown as blue arrows. These changes can be ordered in time, starting from the extant population P1, and going back in time to their common ancestor (here arbitrarily placed at $z(4)$), and then forward again in time to population P2. In haploids, the value of the hybrid index at each point in the chain, is the proportion of P2 alleles that the genotype contains, i.e., $h = i/d$ for phenotype $z(i)$. In real hybrids between P1 and P2, the substituted alleles may appear in random combinations due to segregation and recombination. The expected phenotype of such a hybrid can be found by randomizing the order of the m_i, and then locating the point in the chain with the required hybrid index, h. When their order is randomized, the chain of phenotypic changes can approximate a Brownian bridge between the parental phenotypes.

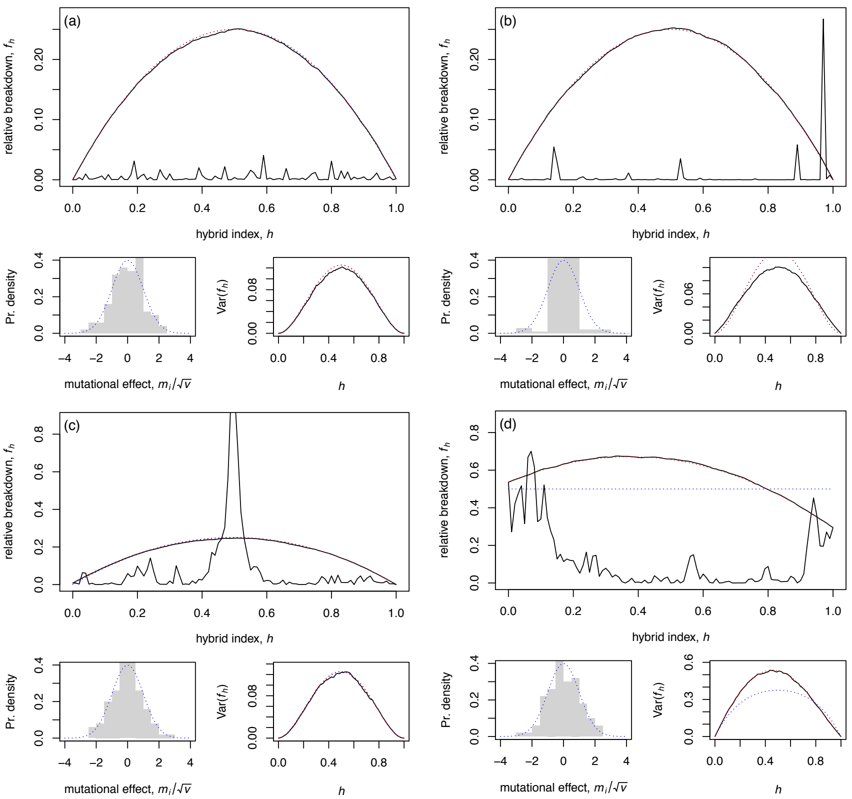

Figure S2: The breakdown associated with haploid hybrid genotypes, under simple models of phenotypic divergence. Each genotype is a hybrid between parental lines P1 and P2, which differ by d substitutions, and so a genotype with hybrid index h, carries $d(1-h)$ alleles from P1, and dh alleles from P2. Each group of plots represents a single simulation of $d = 100$ substitutions, for which $d+1$ trait values were generated at random. In the larger plots of each group, the black lines show the standardized breakdown score, which depends on the squared deviation of the trait from its optimal value: $f_h \equiv z^2(dh)/vd$. The jagged lines show the values when the hd P2 alleles were added in a fixed order, reflecting the chain of substitutions that connects P1 to P2 via their common ancestor (see Fig. S1). (This common ancestor carries the ancestral alleles from both lines, and will therefore be characterized by an intermediate value of h.) The smoother, black curves show results when the hd alleles were chosen at random, to simulate the generation of a hybrid by random segregation and recombination (curves show the average of 10,000 such randomizations). These results are very close to expectations for a Brownian bridge (see colored dotted lines). The analytical predictions are compared when we account correctly for the trait values of the parents (red dotted lines, which show eqs. 24-25), and when we use the approximation of eq. 26 with parental maladaptation set at its expected value (blue dotted lines). Smaller subplots show the effects of the $d = 100$ substitutions (eq. 27; left-hand smaller plots), and the variance in breakdown across the 10,000 randomizations (right-hand smaller plots; see eq. 23). Each group of plots shows a different set of assumptions about the original divergence of P1 and P2. In panel (**a**), we assumed that the ancestral populations always remained close to the optimum, by drawing the intermediate phenotypes from a normal distribution (see eq. 28). In panel (**b**) we violated the assumption of normality, by drawing the phenotypes from a Cauchy distribution with an undefined variance. This led to some mutations having very large effects, but made little difference to the accuracy of the Brownian bridge. In panel (**c**), we assumed that the ancestral population was distant from the optimum, and that the populations adapted in parallel. This was achieved by generating the phenotypes according to an Ornstein-Uhlenbeck process. Lastly, in panel (**d**) we assumed that populations diverged without any effective natural selection, by drawing the mutational effects from uncorrelated normal distributions. This situation would lead to $E(f_{\mathrm{P}}) = 1/2$ and might apply to divergence under mutation accumulation, or strong inbreeding.

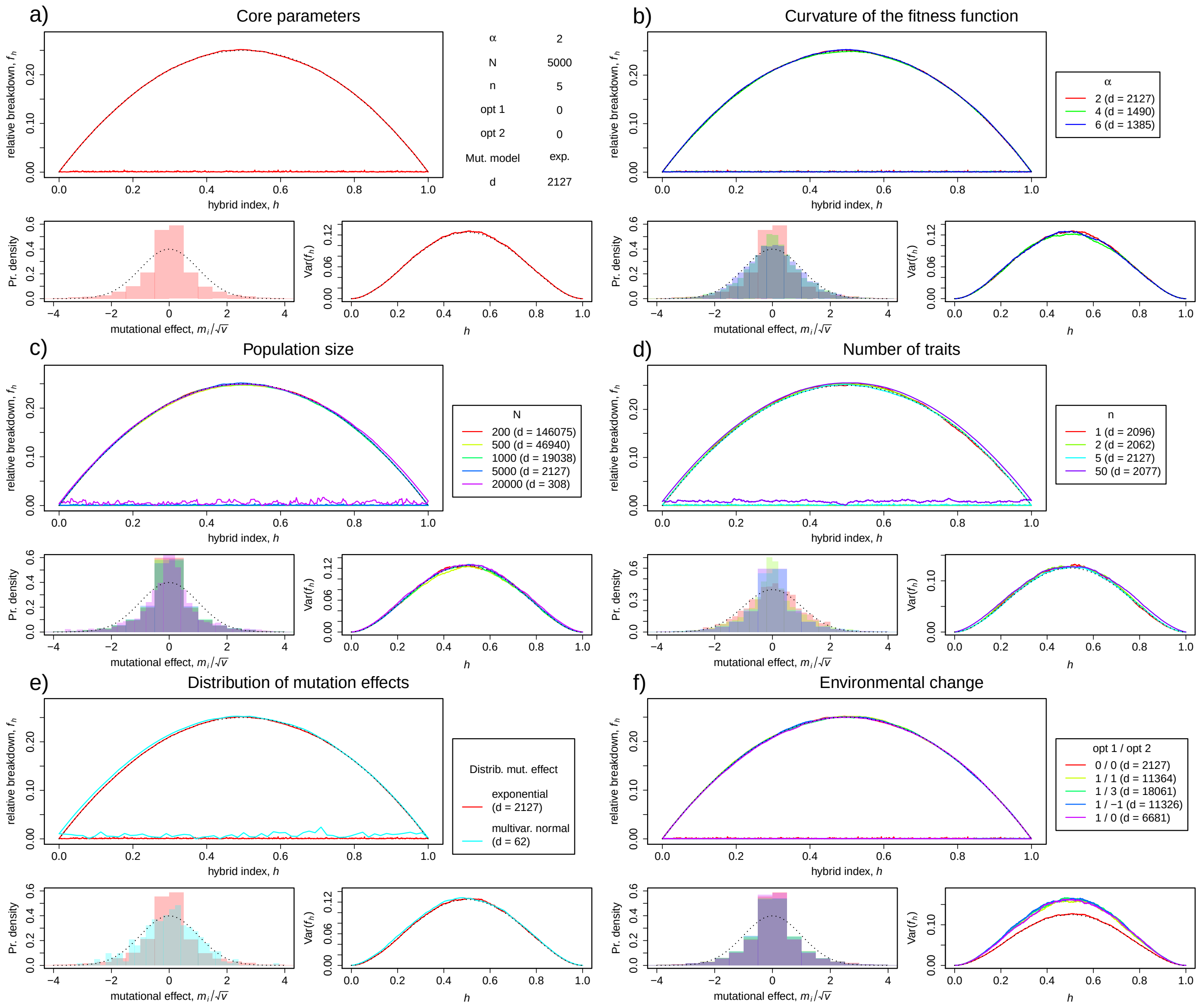

Figure S3: The breakdown associated with haploid hybrid genotypes, under explicit population genetic simulations of phenotypic divergence. The full simulation methods are described by Fraïsse et al. (2016b), but in each case, we assumed haploid genomics, and tracked the appearance, fixation or loss of mutations one at a time. Panel (**a**) shows results for a core parameter set. In subsequent panels, we varied the curvature of fitness function (α; panel **b**; eq. 1); the size of each of the parental populations (N; panel **c**), the number of phenotypic traits (n; panel **d**; eq. 4); the shape of the distribution of mutational effects (exponential versus multivariate normal; panel **e**), and the model of environmental change (panel **f**). In particular, we moved the phenotypic optima for one of the n traits, according to a smooth sin wave function, which returned to its starting point 100 times over the course of a simulation. The optima tracked by the two populations sometimes differed, with the optimum tracked by P1 moving between 0 and opt1, and the optimum tracked by P2 between 0 and opt2. Each simulation comprised an expected 10^7 new mutations. We assumed that selection on all traits was isotropic (all $\lambda_i = 1$; eq. 4); the width of the mutational distribution was set such that the mean selection coefficient of a mutation appearing in an optimal genotype was -0.01, and the constant of proportionality in eq. 1 was set at 0.5. The number of mutations that fixed in each simulation (d) is indicated in parentheses in the legends. All other details match Figure S2.

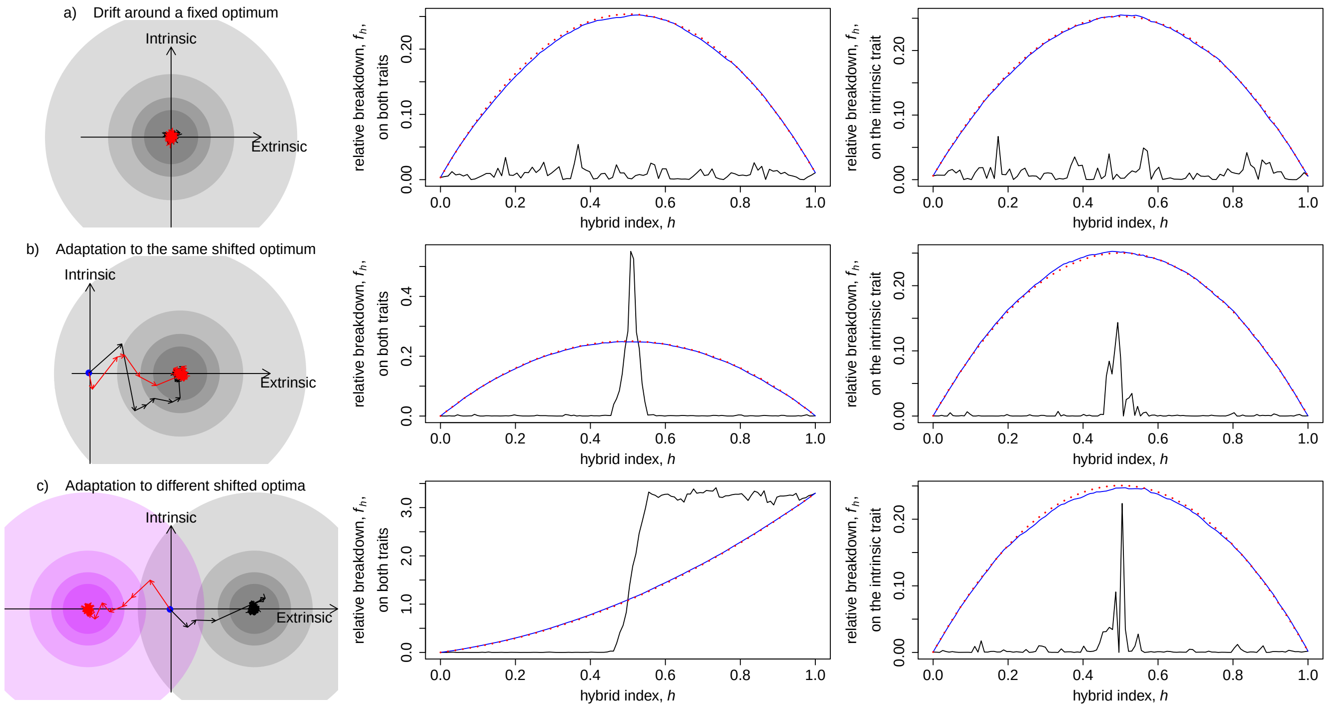

Figure S4: The breakdown associated with haploid hybrid genotypes, under explicit population genetic simulations of phenotypic divergence, in scenarios involving discrete jumps in the optimal value for one of $n = 2$ traits. This allows us to distinguish between intrinsic isolation (involving the maladaptation of traits whose optimum never moves), and extrinsic isolation (involving traits whose optimal value can vary between environments). Left-hand panels represent the fitness landscapes experienced by P1 and P2, with the initial optima found at the origin, and the concentric circles showing its final position, to which the parental populations adapt. Arrows show the phenotypic effects of each mutation that fixed during the divergence (red for P1 and black for P2). The central column shows the breakdown due to maladaptation in both traits, measured in the environment experienced by P1. The right-hand column shows breakdown due to maladaptation in one of the two traits. This is equivalent to measuring a single component of fitness, or to measuring total fitness in an environment where selection does not act on one of the traits. Row (**a**) corresponds to drift around a common fixed optimum for the two populations. Row (**b**) shows a scenario in which the optimum moves in the same way for both diverging populations. Row (**c**) shows a scenario in which the two populations adapt to different shifted optima, such that $f_{P2} \simeq 3$. Cases (**a**) and (**b**) correspond to mutation order speciation by drift or adaptation respectively. Case (**c**) represent an example of ecological speciation. We arbitrarily chose parameters so that the ancestral population suffered an approximately 12% drop in fitness after the environmental shift. All other simulation methods and details are as in Figure S3.

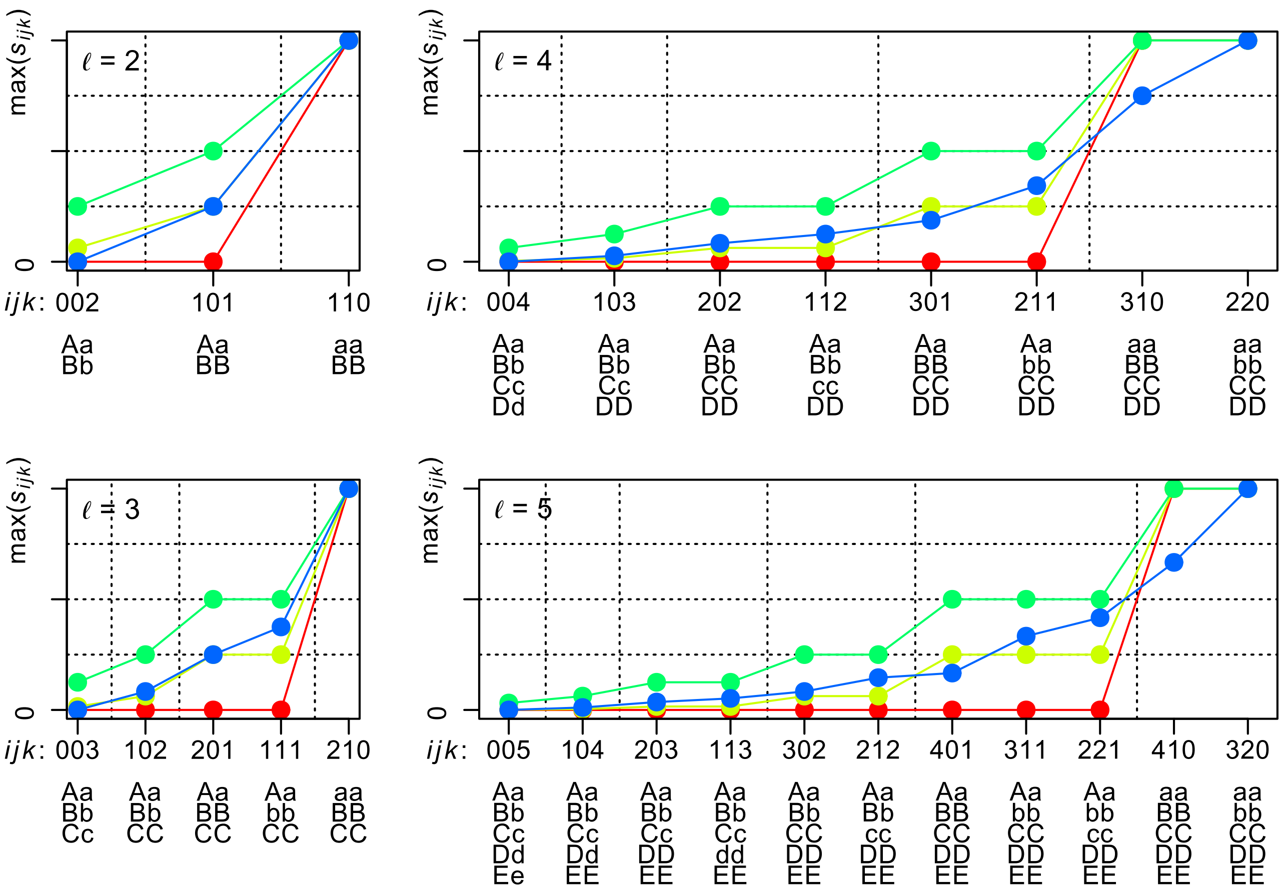

Figure S5: The effects of an incompatibility on hybrid breakdown score, s_{ijk} (eq. 41), when the incompatibility appears in a genotype comprising i loci that are homozygous for alleles from one parental species, j loci that are homozygous for alleles from the other parental species, and k loci that are heterozygous. The four panels show values for incompatibilities involving $\ell = 2, 3, 4$ and 5 loci. Values are compared when incompatible combinations have multiplicative effects (green points; eq. 42 with $\delta = 0$), when incompatibilities are wholly recessive (red points; eq. 42 with $\delta \to \infty$), and when they show partial recessivity at a level sufficient to generate Haldane's Rule (eq. 42 with $\delta = 2$; and treating hemizygous loci as equivalent to homozygous loci). Also shown are values assigned according to eq. 53 (blue points), which exactly reproduce the dependencies predicted by Fisher's geometric model with well adapted parents (eq. 9). Shown on the horizontal axis are the ijk values, and below, are example diploid genotypes, with upper and lower case letters used to distinguish alleles from the two parental lines, P1 and P2. Vertical dotted lines group genotypes with the same level of heterozygosity (i.e., the same value of k).

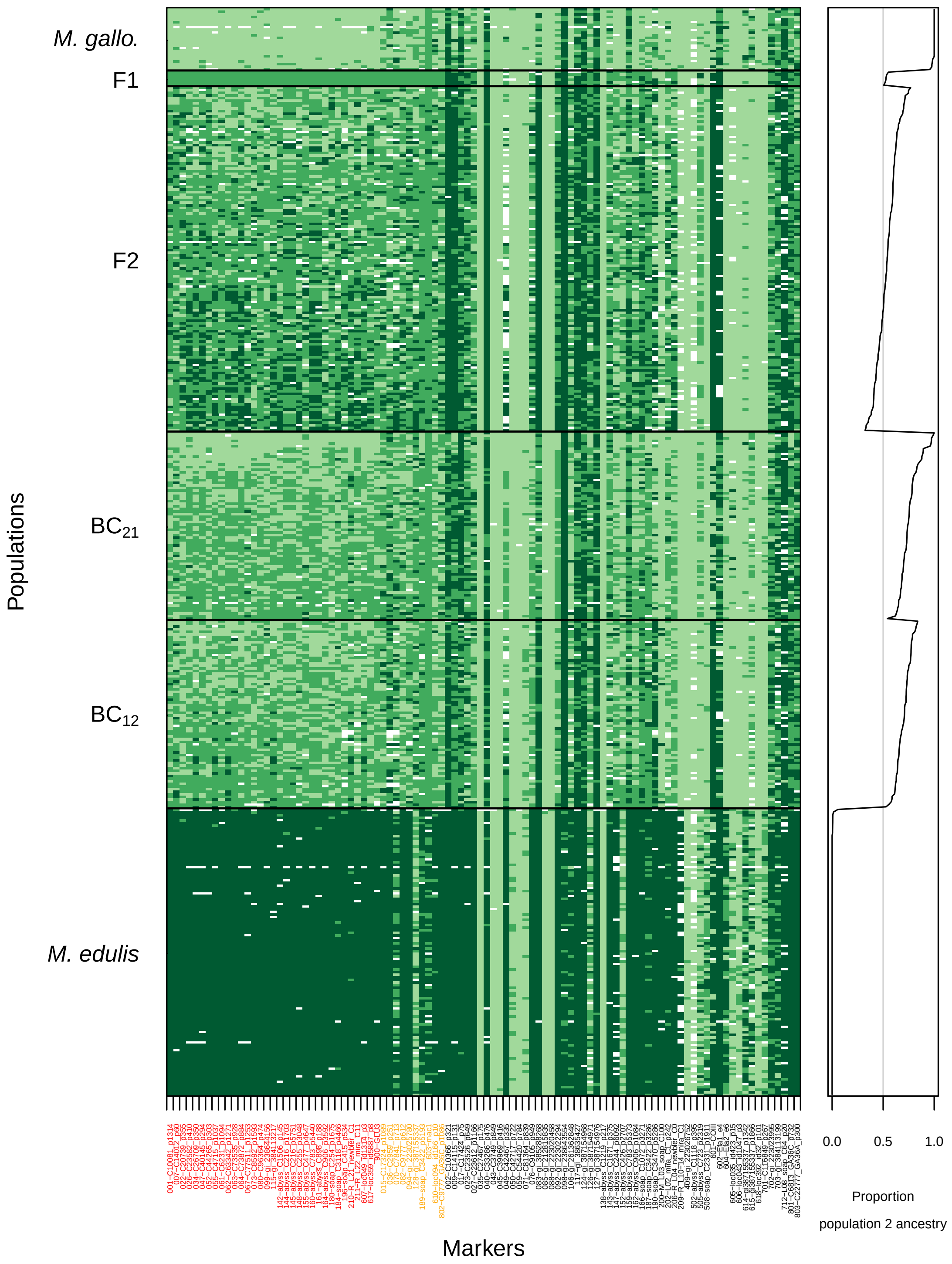

Figure S6: Genotype plot for the raw *Mytilus* data. This plot was generated with the R package *introgress* (v1.2.3; Gompert and Buerkle 2010). Columns represent markers, and rows represent individuals, labeled by cross type. Note that both backcrosses were made to *M. galloprovincialis*, and the subscripts differ according to whether the F1 was the female parent (BC_{12}), or the male parent (BC_{21}). Each colored rectangle represents the state of the genotype as homozygous for the *M. edulis* allele (dark green), homozygous for the *M. galloprovincialis* allele (light green), heterozygous (mid green), or missing data (white). The 33 markers that were strongly differentiated between the species are labeled in red, and the additional 10 that were scored as homozygous in all 6 F1 are labeled in orange. The right-hand-side plot represents the estimated proportion of *M. galloprovincialis* ancestry (population 2).

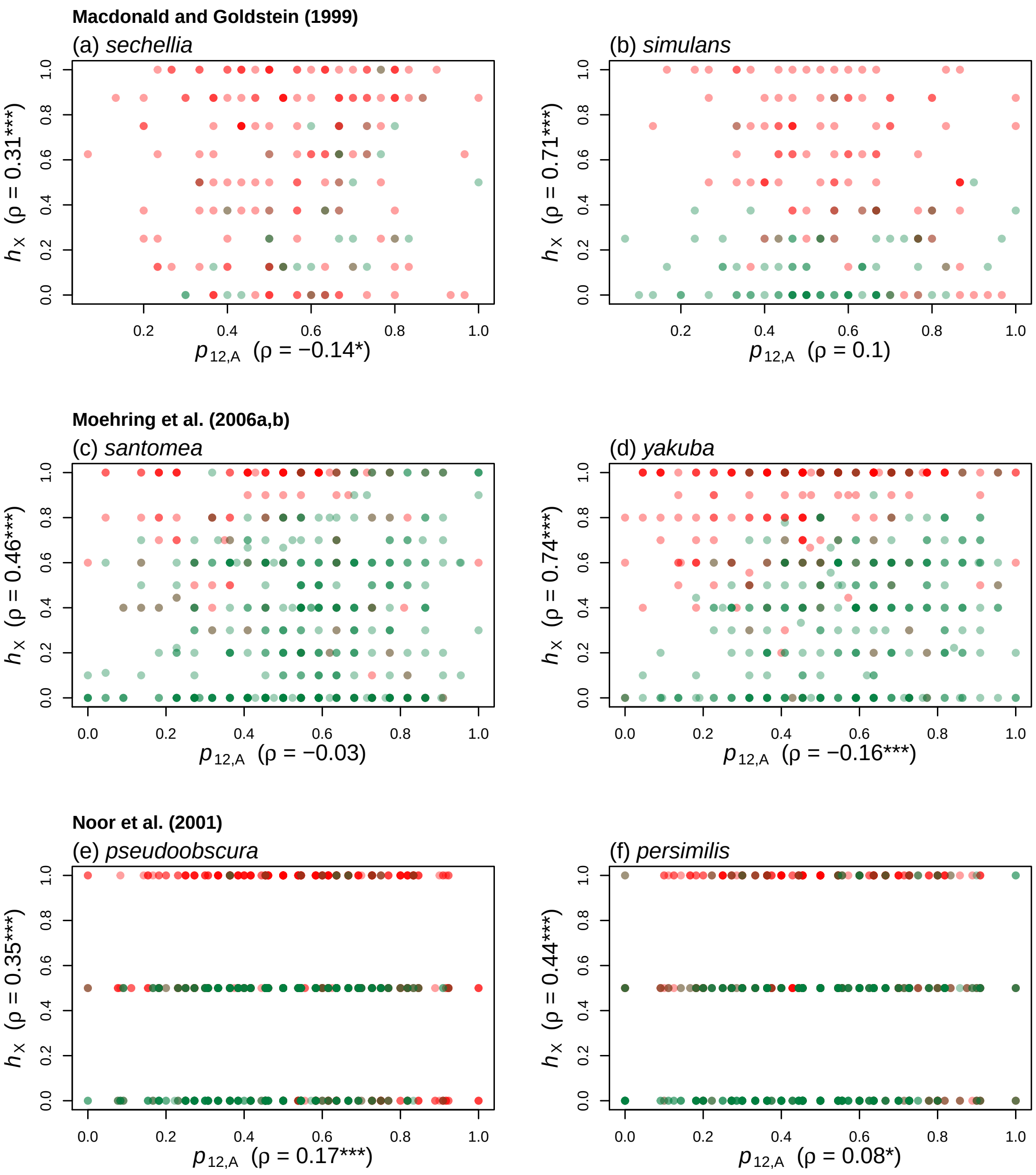

Figure S7: Plots of the *Drosophila* male backcross data reanalyzed here (see Table 1 and Moehring 2011). For all species pairs F1♂ had low fertility, consistent with Haldane's Rule, and so male hybrids were derived from the backcross F1♀ × P1♂. Each point represents an individual hybrid, and is plotted as a function of its interspecies heterozygosity on the autosomes ($p_{12,A}$), and its hybrid index on the X (h_X), as estimated from a variable number of markers. Each row contains data from a reciprocal backcross, with the left-hand panel showing the backcross to the maternal species from the F1. Each individual was also scored for "sterility" on an ordinal scale, and red points show individuals with the highest possible sterility score (Panels **a**, **b**, **e**, and **f**), or falling within one of the two highest classes (Panels **c**-**d**). In parentheses, each axis also contains the results of correlating $p_{12,A}$ or h_X with sterility scores, replicating results of Moehring (2011). Shown are the Pearson's correlation coefficients, ρ, and a measure of significance of a Spearman's rank correlation (* $p < 0.05$; ** $p < 0.01$; *** $p < 0.001$). In all cases, there was a strong tendency for h_X to correlate positively with sterility scores, while the correlations with $p_{12,A}$ show no consistent pattern. These findings are consistent with predictions from Fisher's geometric model (see Fig. S8). Panels (**e**) and (**f**) show that h_X was estimated from two markers in this study, providing a natural division of the data into high-h_X and low-h_X individuals see Table 3.

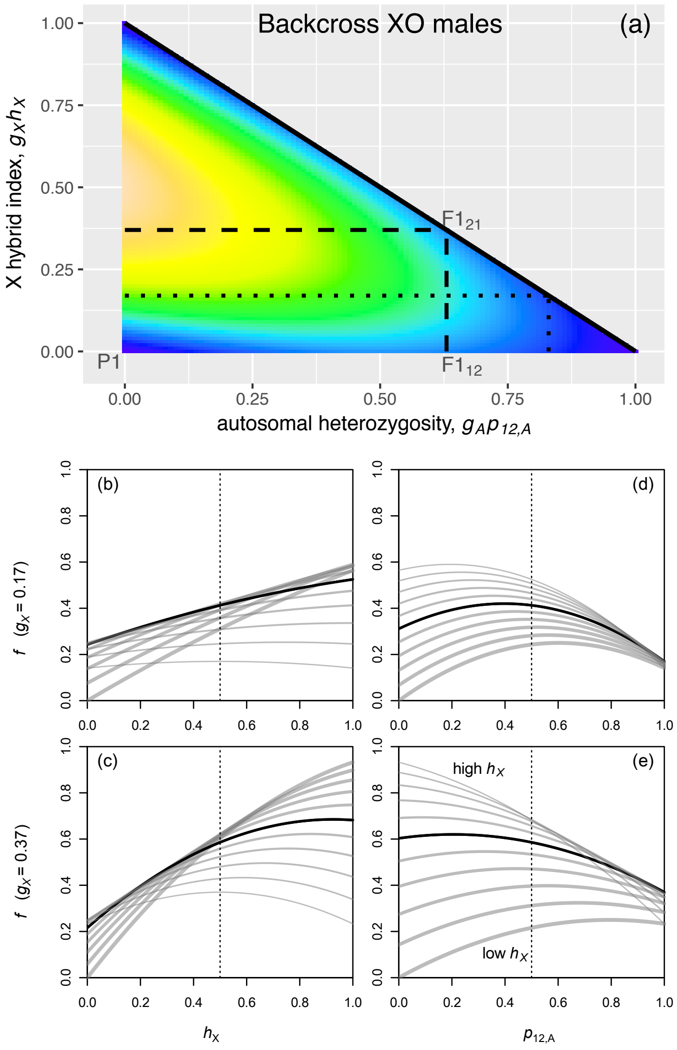

Figure S8: Predictions of Fisher's geometric model for heterogametic male hybrids. For simplicity, the predictions neglect any contributions from uniparentally-inherited loci other than the X, and assume that the parental types are well adapted. For concreteness, we assume XO sex determination, so that hybrids differ in their autosomal heterozygosity, $p_{12,A}$ and hybrid index of the X, h_X (i.e., for a backcross to P1, the proportion of divergent alleles on the X that come from P2). Panel (**a**) shows the fitness surface as a function of these two quantities. The dashed lines delimit the region that would apply to a species pair with $g_X = 0.17$ (as we have estimated for *Drosophila simulans/sechellia* and *D. santomea/yakuba*), and the dotted lines delimit the region that would apply to a species pair with $g_X = 0.37$ (as we have estimated for *Drosophila persimilis/pseudoobscura*). This region matches the parallelogram of Figure 1c. Panels (**b**)-(**e**) show slices through this fitness surface, with vertical dotted lines showing the Mendelian expectations for a first backcross. Panels (**b**) and (**c**) show the dependency on h_X. Results are shown when the autosomal heterozygosity is equal to its Mendelian expectation of $E(p_{12,A}) = \frac{1}{2}$ (black line), and over a range of values from $p_{12,A} = 0$ (thickest gray line) to $p_{12,A} = 1$ (thinnest gray line). Similarly, panels (**d**) and (**e**) show the dependency on autosomal heterozygosity, when h_X is at its expected value of $E(h_X) = \frac{1}{2}$ (black line), or over a range of values from $h_X = 0$ (thickest gray line) to $h_X = 1$ (thinnest gray line). Together, the plots show that Fisher's geometric model can account for the surprising results of Moehring (2011), and generate a new supported prediction (Table 3).

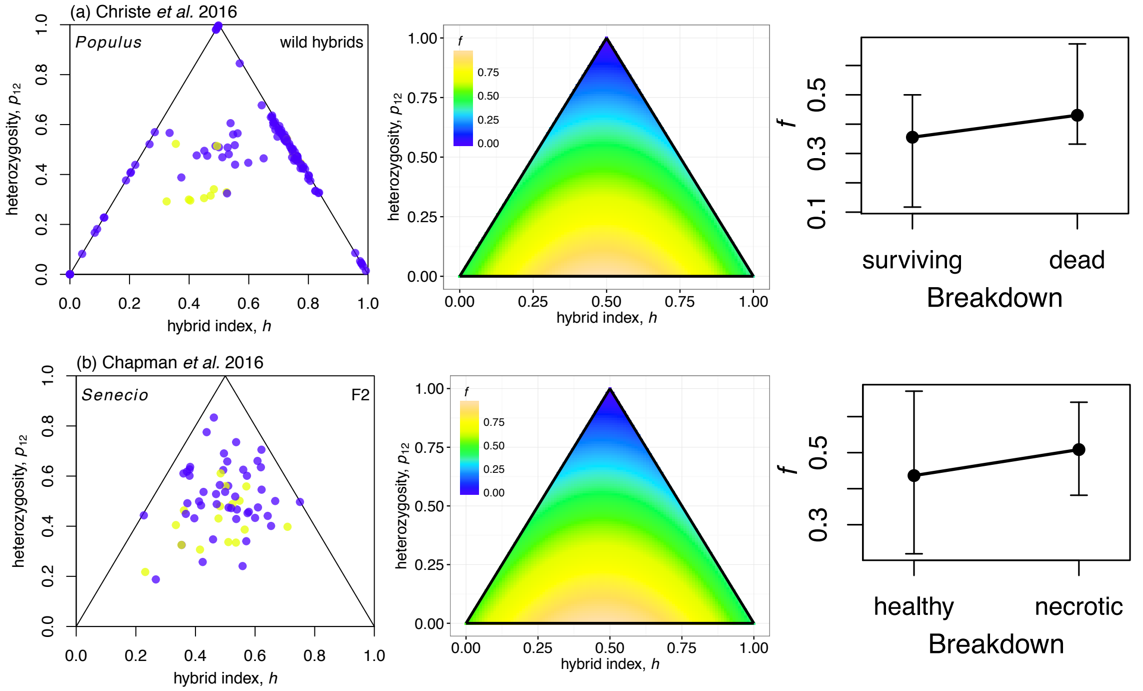

Figure S9: Estimation of the fitness surface for interspecific hybrids from plants (Table 1), namely wild hybrids of the genus *Populus* (row (**a**); Christe et al. 2016), and an F2 cross of the genus *Senecio* (row (**b**); Chapman et al. 2016). The left-hand panels show the data. Each point represents an individual hybrid, plotted as a function of their hybrid index, h and inter-species heterozygosity, p_{12}. Hybrid breakdown was scored as a binary scale for both data sets (Table 1), and the points are colored as low breakdown (blue) or high breakdown (yellow). The central panels show the fitness surfaces estimated from these data, using binary logistic regression, with predictors h, h^2 and p_{12} (see eq. 17, and Tables S5 and S6). These estimated surfaces resemble theoretical predictions with intermediate levels of parental maladaptation (compare Fig. 2a-b), which is consistent with evidence of F1 hybrid vigor in these taxa (*Populus*: Caseys et al. 2015; *Senecio*: Abbott and Brennan 2014). The right-hand panels show the fitted values of the predictor, for the high- and low-breakdown categories. Points and confidence intervals show the median and 90% quantiles.

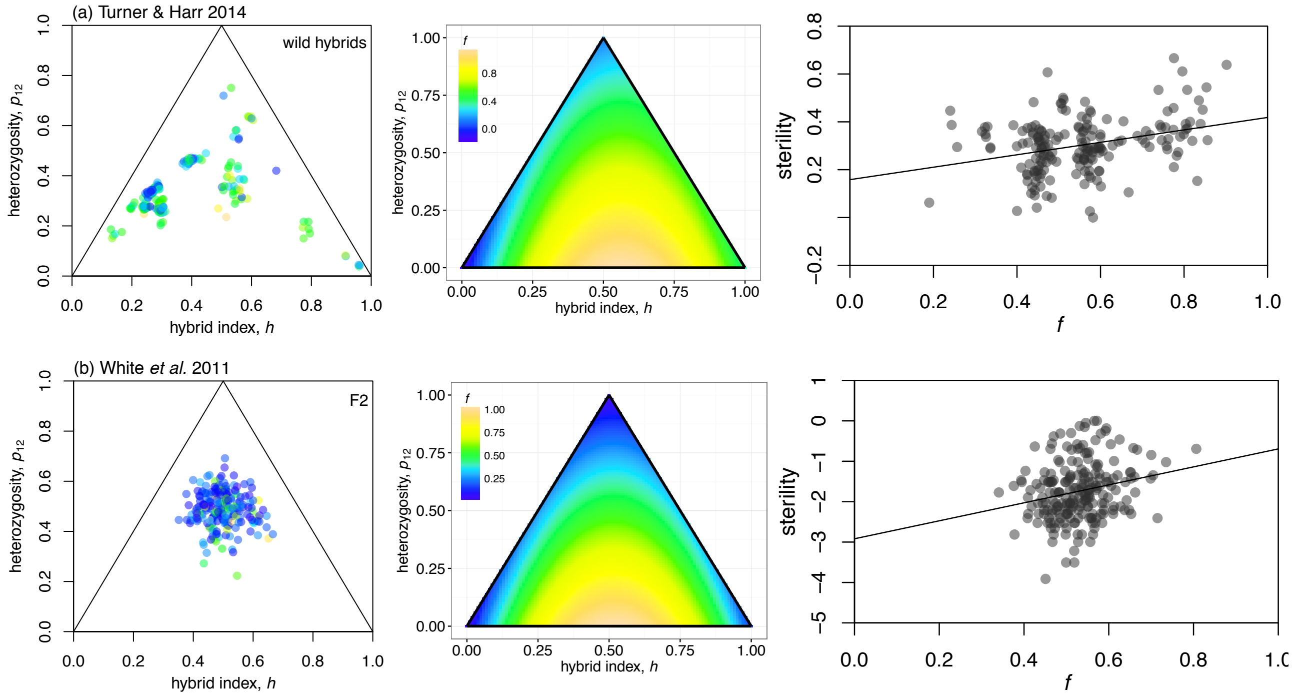

Figure S10: Estimation of the fitness surface for subspecific hybrids from *Mus musculus* (Table 1, White et al. 2011; Turner and Harr 2014). Because these males are heterogametic, to calculate the hybrid index, hemizygous alleles were treated as equivalent to homozygous alleles. Row (**a**) shows wild hybrids, with testes size as a proxy for fertility (Turner and Harr 2014), while row (**b**) shows an F2 cross, using the proportion of abnormal sperm (White et al. 2011). Since breakdown was scored on a continuous scale, the left-hand panels colors each point with the color scheme of the fitness surfaces. The fitness surfaces were fit by standard least-squares regression, as shown in the right-hand panels. All other details match Supplementary Figure S9.

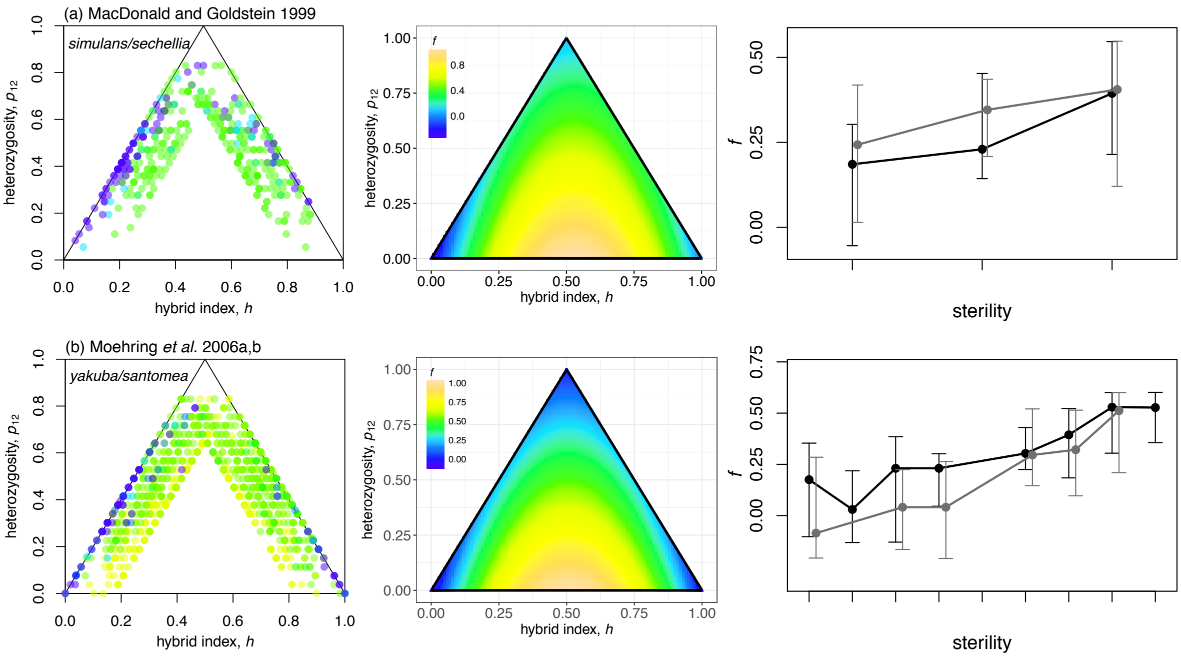

Figure S11: Estimation of the fitness surface for backcross male hybrids from *Drosophila* species pairs (Table 1, Macdonald and Goldstein 1999; Moehring et al. 2006a,b). For these data, the proxy for breakdown, male sterility, was scored on an ordinal scale, and so the models were fit with proportional odds logistic regression. The right-hand panels group the fitted values of the linear model by sterility category, and do so separately for the two backcross directions (black lines showing results for backcrosses to the maternal species from the F1, and gray lines show the backcross to the paternal species). This shows that the same patterns appear in both halves of the data. All other details match Supplementary Figures S9 and S10.

Table S1: The contribution of parental maladaptation to hybrid breakdown

	Description	Condition	f_{mal}
(a)	Exact result	-	$\frac{\sum_i \lambda_i ((1-h) z_{\mathrm{P1},i} + h z_{\mathrm{P2},i})^2}{d \sum_i \lambda_i v_i}$
(b)	Independent maladaptation	$\overline{z_{\mathrm{P1},i} z_{\mathrm{P2},i}} = 0$	$(1-h)^2 f_{\mathrm{P1}} + h^2 f_{\mathrm{P2}}$
(c)	Identical parental phenotypes	$z_{\mathrm{P1},i} = z_{\mathrm{P2},i}$	f_{P}
(d)	Optimal midparent	$z_{\mathrm{P1},i} + z_{\mathrm{P2},i} = 0$	$(1-2h)^2 f_{\mathrm{P}}$

Table S2: Checks for appropriateness for genomic data sets

Hybridization		N	#markers	Cross	Fitness measure, w	p_0: mean (sd)	ρ_{w,p_0} (p-val)	ρ_{p_{12},p_0} (p-val)	Reference
Mytilus	*edulis/galloprovincialis*	132	43	F2	-	0.034 (0.074)	-	0.244 (0.005**)	This study
		144	43	BC1	-	0.019 (0.045)	-	0.302 (0.0002***)	This study
Senecio	*aethnensis/chrysanthemifolius*	64	966	F2	Necrotic/Healthy	0.013 (0.014)	0.145 (0.253)	0.273 (0.029*)§	Chapman et al. 2016
Mus musculus	*musculus/domesticus*	185	14,220	WH	Testes weight	0.012 (0.006)	-0.099 (0.181)	0.039 (0.595)	Turner and Harr 2014
Mus musculus	*musculus/domesticus*	310	182	F2	Prop. abnormal sperm	0.030 (0.046)	0.056 (0.329)	-0.071 (0.215)	White et al. 2011
Daphnia magna	Germany/Finland	353	1324	F2	Fecundity ($N = 193$)	0.124 (0.092)	0.138 (0.056)	-0.206 ($<10^{-4}$**)	Routtu et al. 2014
Medicago sativa	*falcata/caerulea*	152	103	F2	Binary survival	0.158 (0.146)	-0.511 ($<10^{-9}$**)	-0.125 (0.124)	Li et al. 2011
Saccharomyces	*cerevisiae/paradoxus*	89	93	F2	Growth rate	0.080 (0.067)	-0.571 ($<10^{-8}$**)	-	Xu and He 2011

Notes: * $p < 0.05$; ** $p < 0.01$;*** $p < 0.001$. p_0 is the proportion of markers with missing data for each individual. For the data of White et al. (2011) this was calculated for autosomal markers only, while for the data of Xu and He (2011) it was defined as the proportion of loci scored as heterozygous, because for these haploid spores, heterozygotes must represent sequencing error, or aneuploidy. ρ and p-val are from Spearman's rank correlation tests. ρ_{w,p_0} is the correlation between fitness and the proportion of missing data; ρ_{p_{12},p_0} is the correlation between the proportion of sites with missing data, and the proportion scored as heterozygous. § this correlation is attributable to two outlying points. All other details match Table 1.

Table S3: The reference populations for *Mytilus* crosses

Species	Location	N	Latitude	Longitude
Mytilus galloprovincialis	Thau, France	22	43.412268	3.691788
Mytilus edulis	The Netherlands	22	53.31	5.424
	Saint-Jouin, Normandy, France	27	49.643953	0.152643
	Villerville, Normandy, France	29	49.399352	0.116054
	Réville, Normandy, France	29	49.5746667	-1.2293333333

Table S4: Expected breakdown scores for homogametic female hybrids with paternal X silencing

Hybrid	Cross (♀ × ♂)	$E(p_{12})$	$E(p_1)$	$E(p_2)$	$E(h)$	$f;\, p_{12} = E(p_{12}), h = E(h)$
Parental	P1 × P1	0	1	0	0	0
	P2 × P2	0	0	1	1	f_{P2}
F1	$\mathrm{F1}_{12} = \mathrm{P1} \times \mathrm{P2}$	$1 - \pi g_X$	πg_X	0	$\frac{1-\pi g_X}{2}$	$\pi g_X - \pi^2 g_X^2 + \frac{(1-\pi g_X)^2}{4} f_{\mathrm{P2}}$
	$\mathrm{F1}_{21} = \mathrm{P2} \times \mathrm{P1}$	$1 - \pi g_X$	0	πg_X	$\frac{1+\pi g_X}{2}$	$\pi g_X - \pi^2 g_X^2 + \frac{(1+\pi g_X)^2}{4} f_{\mathrm{P2}}$
BC to P1	$\mathrm{P1} \times \mathrm{F1}_{12}$	$\frac{1-g_X}{2}$	$\frac{1+g_X}{2}$	0	$\frac{1-g_X}{4}$	$\frac{1-g_X^2}{4} + \frac{(1-g_X)^2}{16} f_{\mathrm{P2}}$
	$\mathrm{F1}_{12} \times \mathrm{P1}$	$\frac{1-\pi g_X}{2}$	$\frac{1}{2}$	$\frac{\pi g_X}{2}$	$\frac{1+\pi g_X}{4}$	$\frac{1-\pi^2 g_X^2}{4} + \pi g_X + \frac{(1+\pi g_X)^2}{16} f_{\mathrm{P2}}$
	$\mathrm{F1}_{21} \times \mathrm{P1}$	$\frac{1-\pi g_X}{2}$	$\frac{1}{2}$	$\frac{\pi g_X}{2}$	$\frac{1+\pi g_X}{4}$	$\frac{1-\pi^2 g_X^2}{4} + \pi g_X + \frac{(1+\pi g_X)^2}{16} f_{\mathrm{P2}}$
	$\mathrm{P1} \times \mathrm{F1}_{21}$	$\frac{1-(2\pi-1)g_X}{2}$	$\frac{1+(2\pi-1)g_X}{2}$	0	$\frac{1-(2\pi-1)g_X}{4}$	$\frac{1-(2\pi-1)^2 g_X^2}{4} + \frac{(1-(2\pi-1)g_X)^2}{16} f_{\mathrm{P2}}$
BC to P2	$\mathrm{P2} \times \mathrm{F1}_{21}$	$\frac{1-g_X}{2}$	0	$\frac{1+g_X}{2}$	$\frac{3+g_X}{4}$	$\frac{1-g_X^2}{4} + \frac{(3+g_X)^2}{16} f_{\mathrm{P2}}$
	$\mathrm{F1}_{21} \times \mathrm{P2}$	$\frac{1-\pi g_X}{2}$	$\frac{\pi g_X}{2}$	$\frac{1}{2}$	$\frac{3-\pi g_X}{4}$	$\frac{1-\pi^2 g_X^2}{4} + \pi g_X + \frac{(3-\pi g_X)^2}{16} f_{\mathrm{P2}}$
	$\mathrm{F1}_{12} \times \mathrm{P2}$	$\frac{1-\pi g_X}{2}$	$\frac{\pi g_X}{2}$	$\frac{1}{2}$	$\frac{3-\pi g_X}{4}$	$\frac{1-\pi^2 g_X^2}{4} + \pi g_X + \frac{(3-\pi g_X)^2}{16} f_{\mathrm{P2}}$
	$\mathrm{P2} \times \mathrm{F1}_{12}$	$\frac{1-(2\pi-1)g_X}{2}$	0	$\frac{1+(2\pi-1)g_X}{2}$	$\frac{3+3(2\pi-1)g_X}{4}$	$\frac{1-(2\pi-1)^2 g_X^2}{4} + \frac{(3+(2\pi-1)g_X)^2}{16} f_{\mathrm{P2}}$

Notes: All expected breakdown scores were calculated from eq. 10, which assumes that parental species P1 is optimal, while P2 may be maladapted with breakdown $f_{\mathrm{P2}} \geq 0$. $0 \leq g_X \leq 1$ denotes the proportion of the divergence that is located on the X chromosome; and $0 \leq \pi \leq 1$ denotes the proportion of the parental X chromosome that is silenced. The quantities p_{12}, p_1, p_2 and h are calculated without considering silenced alleles on the paternal X, because these make no contribution to the phenotype.

Table S5: Inferring the hybrid fitness surface from genomic data sets

Hybridization	N	Response variable	β_0	β_1	β_2	r^2	AIC
Populus	137	Binary: Survival	[0]	-	-	[0]	104.75
alba/tremula			5.33	[4]	[1]	0.129	93.485
			12.44	2.229	[1]	0.225	**85.647**
			11.66	2.279	1.028	0.228	87.293
Senecio F2	64	Binary: Necrotic/Healthy	[0]	-	-	[0]	73.979
aethnensis/chrysanthemifolius			6.13	[4]	[1]	0.0857	**69.807**
			6.54	1.797	[1]	0.0940	71.210
			6.42	1.963	1.065	0.0951	73.136
Mus musculus ♂	185	-log(testes weight)	[0]	-	-	[0]	140.39
musculus/domesticus			0.82	[4]	[1]	0.0638	130.19
			0.75	4.425	[1]	0.0645	132.06
			0.83	4.622	0.888	0.1090	**125.04**
Mus musculus F2♂	213	log(prop. abnormal sperm)	[0]	-	-	[0]	465.09
musculus/domesticus			2.22	[4]	[1]	0.0480	**456.62**
			2.23	3.430	[1]	0.0481	458.60
			2.23	3.933	0.968	0.0486	460.48
Drosophila BC1♂	1099	Ordinal: Motile sperm	[0]	-	-	[0]	2702.84
yakuba/santomea			12.95	[4]	[1]	0.1928	2186.39
			12.24	4.347	[1]	0.1960	2179.90
			12.51	4.413	0.986	0.1986	**2174.87**
Drosophila BC1♂	400	Ordinal: Sperm quantity	[0]	-	-	[0]	679.57
simulans/sechellia			13.24	[4]	[1]	0.1816	558.89
			11.68	5.006	[1]	0.2022	546.97
			11.45	5.314	0.945	0.2541	**513.90**

Notes: r^2 is the proportion of the variance explained, or McFadden's pseudo-r^2 for ordinal or binary data. AIC: Akaike Information Criterion, with favored model in bold. Parameter values that were not estimated, but fixed at their null or theoretically predicted values are shown in square brackets.

Table S6: The significance of individual regression coefficients

Hybridization	N	Response variable	intercept	h	h^2	p_{12}	r^2	AIC
Populus	137	Binary: Survival	-2.63†	8.229**	-7.685**	-2.963**	-	-
			-1.831	26.564**	-27.301***	-11.600***	0.228	-
Senecio F2	64	Binary: Necrotic/Healthy	-0.863	12.606	-13.430	-6.422*	0.095	73.14
			1.714	-	-	-5.959*	0.088	**69.62**
Mus musculus	185	-log(testes weight)	-2.732	3.816***	-3.388***	-0.8256**	0.109	-
		1-(testes weight)/max(testes weight)	0.106	1.612***	-1.400***	-0.339*	0.106	-
Mus musculus F2	213	log(prop. abnormal sperm)	-2.881	8.749	-8.466	-2.225**	0.049	460.48
			-0.691	-	-	-2.150**	0.045	**457.20**
	212	-sqrt(density of normal sperm)	-3.992	10.159	-9.378	-3.404**	0.048	649.41
			-1.320	-	-	-3.348**	0.045	**645.99**
	305	log(prop. abnormal sperm)	-2.498	6.811	-6.533	-3.340***	0.037	655.00
			-0.773	-	-	-1.929**	0.035	**652.76**
Drosophila yak/sant	1099	Ordinal: Motile sperm	-	55.646***	-54.478***	-12.514***	0.199	-
Drosophila sim/sech	400	Ordinal: Sperm quantity	-	60.899***	-57.551***	-11.461***	0.254	-

Notes: * $p < 0.05$; ** $p < 0.01$;*** $p < 0.001$. r^2 is the proportion of the variance explained by the regression, or McFadden's pseudo-r^2 for binary or ordinal regressions. AIC: Akaike Information Criterion, used for comparing the fit of different sized models to the same data. † Results reproduced from Christe et al. (2016), including "family" as a factor.

Table S7: Information on the 98 markers used for the *Mytilus* genotyping. Flanking sequences are provided in an annex file `Mytilus_markers_flanking_sequences.csv`. Contigs listed here can be obtained on the MytilusDB website (http://www.scbi.uma.es/mytilus/index.php, Fraïsse et al. 2016a), with the exception of TranscriptomeGalloConcat.

Marker name	SNP	Contig	Position
001-C10081_p1314	[A/T]	Contig10081_GA36A	1314
002-C10366_p321	[A/G]	Contig10366_GA36G	321
007-C14012_p60	[A/T]	Contig14012_GA36H	60
009-C14115_p131	[A/T]	Contig14115_GA36B	131
015-C17324_p1089	[A/G]	Contig17324_GA36A	1089
017-C17424_p75	[A/T]	Contig17424_GA36H	75
022-C20739_p355	[A/T]	Contig20739_GA36B	355
023-C23526_p649	[A/T]	Contig23526_GA36A	649
026-C23582_p410	[A/G]	Contig23582_GA36B	410
027-C23612_p1166	[C/T]	Contig23612_GA36D	1166
034-C26709_p350	[A/T]	Contig26709_GA36B	350
035-C27467_p175	[C/T]	Contig27467_GA36B	175
036-C2959_p251	[A/G]	Contig2959_GA36D	251
040-C33286_p476	[A/G]	Contig33286_GA36C	476
043-C3749_p849	[A/T]	Contig3749_GA36A	849
045-C39969_p416	[A/G]	Contig39969_GA36B	416
047-C40145_p294	[A/C]	Contig40145_GA36A	294
049-C42467_p335	[C/T]	Contig42467_GA36A	335
050-C42717_p722	[C/T]	Contig42717_GA36B	722
052-C44265_p303	[C/T]	Contig44265_GA36C	303
055-C4715_p1037	[A/C]	Contig4715_GA36B	1037
059-C54420_p784	[A/G]	Contig54420_GA36A	784
061-C6231_p1094	[A/C]	Contig6231_GA36B	1094
062-C63342_p1271	[A/T]	Contig63342_GA36B	1271
063-C73535_p928	[A/C]	Contig73535_GA36A	928
064-C73872_p884	[C/T]	Contig73872_GA36D	884
067-C77511_p1253	[A/C]	Contig77511_GA36A	1253
070-C7841_p713	[A/G]	Contig7841_GA36D	713
071-C81364_p839	[A/G]	Contig81364_GA36B	839
073-C8592_p1593	[A/T]	Contig8592_GA36D	1593
076-C9105_p949	[A/G]	Contig9105_GA36D	949
080-C96364_p474	[A/G]	Contig96364_GA36A	474
082-C9777_p612	[C/G]	Contig9777_GA36C	612
083-gi_385288268	[A/C]	gi_385288268_emb_Contig56466	1072
085-gi_212815835	[A/C]	gi_212815835_gb_GE749068	643
088-gi_223020458	[A/C]	gi_223020458_gb_FL493225	306
092-gi_223022294	[C/T]	gi_223022294_gb_FL494235	300
094-gi_223025780	[C/T]	gi_223025780_gb_FL490738	366
098-gi_238643554	[G/T]	gi_238643554_gb_Contig12266	389
099-gi_238644156	[A/C]	gi_238644156_gb_Contig35188	123
106-gi_261362848	[A/G]	gi_261362848_gb_M12A_cDNA4	139

115-gi_384113217	[C/T]	gi_384113217_emb_HE609130	794
117-gi_38635427	[C/G]	gi_38635427_emb_AJ609591	80
124-gi_387154968	[A/C]	gi_387154968_emb_HE609112	131
126-gi_387154971	[A/G]	gi_387154971_emb_Contig4866	1908
127-gi_387154976	[C/T]	gi_387154976_emb_HE609129	229
128-gi_387155337	[A/G]	gi_387155337_emb_HE610079	1120
138-abyss_C1219_p217	[A/T]	H_L1_abyss_Contig1219	2176
142-abyss_C1506_p145	[A/G]	H_L1_abyss_Contig1506	1455
143-abyss_C1671_p375	[A/G]	H_L1_abyss_Contig1671	3759
144-abyss_C216_p1703	[A/C]	H_L1_abyss_Contig216	1703
145-abyss_C216_p5751	[A/T]	H_L1_abyss_Contig216	5751
147-abyss_C244_p6092	[A/G]	H_L1_abyss_Contig244	6092
148-abyss_C255_p3048	[A/G]	H_L1_abyss_Contig255	3048
152-abyss_C426_p2707	[C/T]	H_L1_abyss_Contig426	2707
155-abyss_C477_p4647	[A/T]	H_L1_abyss_Contig477	4647
159-abyss_C783_p5373	[A/G]	H_L1_abyss_Contig783	5373
160-abyss_C783_p5440	[A/G]	H_L1_abyss_Contig783	5440
161-abyss_C898_p188	[C/T]	H_L1_abyss_Contig898	188
162-abyss_C906_p1084	[G/T]	H_L1_abyss_Contig906	1084
164-abyss_C906_p3592	[G/T]	H_L1_abyss_Contig906	3592
166-soap_C1072_p3271	[A/T]	H_L1_soap_Contig1072	3271
180-soap_C254_p1675	[G/T]	H_L1_soap_Contig254	1675
184-soap_C3118_p4466	[A/G]	H_L1_soap_Contig3118	4466
187-soap_C3422_p3286	[A/T]	H_L1_soap_Contig3422	3286
189-soap_C3422_p5193	[A/G]	H_L1_soap_Contig3422	5193
190-soap_C3470_p5286	[A/G]	H_L1_soap_Contig3470	5286
196-soap_C415_p534	[A/G]	H_L1_soap_Contig415	534
200-M_L03_soap_C100	[A/G]	M_L03_soap_Contig100	6168
202-L02_mira_C1_p242	[C/T]	R_L02_mira_Contig1	2420
206-R_L04_newbler_C0	[C/T]	R_L04_newbler_contig00002	5658
209-R_L10-14_mira_C1	[A/G]	R_L10-14_mira_Contig1	16260
210-R_L21_newbler_C1	[G/T]	R_L21_newbler_Contig1	18611
211-R_L22_mira_C11	[G/T]	R_L22_mira_Contig11	5294
409-gi_223026752	[A/G]	gi_223026752_gb_Contig46241	515
502-abyss_C1118_p395	[T/C]	H_L1_abyss_Contig1118	3954
505-abyss_C723_p2319	[T/C]	H_L1_abyss_Contig723	2319
508-soap_C2331_p1911	[A/G]	H_L1_soap_Contig2331	1911
601-COIII	[C/T]	mitochondria	-
602-Efa1_e4	[A/T]	R_L02_mira_Contig1	6232
603-mac1	[C/T]	gi_5114427_gb_Port34a	2372
604-Efa2_e6	[A/C]	R_L02_mira_Contig1	6232
605-loc035_id423_p15	[T/A]	TranscriptomeGalloConcat	1593146
606-loc043_id1047_p3	[G/A]	TranscriptomeGalloConcat	3639171
607-loc049_id1314_p3	[T/C]	TranscriptomeGalloConcat	3833043
610-loc094_id166_p10	[A/G]	TranscriptomeGalloConcat	10850806
614-gi387155337_p1342	[GGAA/TGAT]	gi_387155337_emb_HE610079	1342
615-gi387155337_p1866	[A/G]	gi_387155337_emb_HE610079	1866

617-loc359_id5887_p8	[T/C]	TranscriptomeGalloConcat	89998577
618-loc392_id32_p101	[C/T]	TranscriptomeGalloConcat	101064357
701-C116849_p267	[A/T]	Contig116849_GA36C	267
702-gi_223023995	[A/T]	gi_223023995_gb_FL493336	177
703-gi_384113199	[A/C]	gi_384113199_emb_Contig12194	4203
712-L08_soap_C64_p20	[A/G]	M_L08_soap_Contig64	20
801-C6813_GA36C_p732	[C/T]	Contig6813_GA36C	732
802-C9777_GA36C_p1086	[C/T]	Contig9777_GA36C	1086
803-C22777_GA36A_p300	[C/T]	Contig22777_GA36A	300
900-GLU3	[CAATATTAAGTG/TCATATAAAGCT]	gi_961463_dbj_D63778	2261

References

Macdonald, S. J. and D. B. Goldstein (1999). "A Quantitative Genetic Analysis of Male Sexual Traits Distinguishing the Sibling Species *Drosophila Simulans* and *D. Sechellia*". *Genetics* 153.4, pp. 1683–1699.

Moehring, A. J., A. Llopart, S. Elwyn, J. A. Coyne, and T. F. C. Mackay (2006a). "The Genetic Basis of Postzygotic Reproductive Isolation Between *Drosophila Santomea* and *D. Yakuba* Due to Hybrid Male Sterility". *Genetics* 173.1, pp. 225–233.

— (2006b). "The Genetic Basis of Prezygotic Reproductive Isolation Between *Drosophila Santomea* and *D. Yakuba* Due to Mating Preference". *Genetics* 173.1, pp. 215–223.

Gompert, Z. and C. A. Buerkle (2010). "Introgress: A Software Package for Mapping Components of Isolation in Hybrids". *Mol. Ecol. Resour.* 10.2, pp. 378–384.

Li, X., X. Wang, Y. Wei, and E. C. Brummer (2011). "Prevalence of Segregation Distortion in Diploid Alfalfa and Its Implications for Genetics and Breeding Applications". *Theor. Appl. Genet.* 123.4, pp. 667–679.

Moehring, A. J. (2011). "Heterozygosity and Its Unexpected Correlations with Hybrid Sterility". *Evolution* 65.9, pp. 2621–2630.

White, M. A., B. Steffy, T. Wiltshire, and B. A. Payseur (2011). "Genetic Dissection of a Key Reproductive Barrier Between Nascent Species of House Mice". *Genetics* 189.1, pp. 289–304.

Xu, M. and X. He (2011). "Genetic Incompatibility Dampens Hybrid Fertility More Than Hybrid Viability: Yeast as a Case Study". *PLoS ONE* 6.4, e18341.

Abbott, R. J. and A. C. Brennan (2014). "Altitudinal Gradients, Plant Hybrid Zones and Evolutionary Novelty". *Philos. Trans. R. Soc. B Biol. Sci.* 369.1648, p. 20130346.

Routtu, J. et al. (2014). "An SNP-Based Second-Generation Genetic Map of Daphnia Magna and Its Application to QTL Analysis of Phenotypic Traits". *BMC Genomics* 15, p. 1033.

Turner, L. M. and B. Harr (2014). "Genome-Wide Mapping in a House Mouse Hybrid Zone Reveals Hybrid Sterility Loci and Dobzhansky-Muller Interactions". *Elife* 3, e02504.

Caseys, C., C. Stritt, G. Glauser, T. Blanchard, and C. Lexer (2015). "Effects of Hybridization and Evolutionary Constraints on Secondary Metabolites: The Genetic Architecture of Phenylpropanoids in European Populus Species". *PLoS ONE* 10.5, e0128200.

Chapman, M. A., S. J. Hiscock, and D. A. Filatov (2016). "The Genomic Bases of Morphological Divergence and Reproductive Isolation Driven by Ecological Speciation in *Senecio* (Asteraceae)". *J. Evol. Biol.* 29.1, pp. 98–113.

Christe, C. et al. (2016). "Selection against Recombinant Hybrids Maintains Reproductive Isolation in Hybridizing *Populus* Species despite F_1 Fertility and Recurrent Gene Flow". *Mol. Ecol.* 25.11, pp. 2482–2498.

Fraïsse, C., K. Belkhir, J. J. Welch, and N. Bierne (2016a). "Local Interspecies Introgression Is the Main Cause of Extreme Levels of Intraspecific Differentiation in Mussels". *Mol. Ecol.* 25.1, pp. 269–286.

Fraïsse, C., P. A. Gunnarsson, D. Roze, N. Bierne, and J. J. Welch (2016b). "The Genetics of Speciation: Insights from Fisher's Geometric Model". *Evolution* 70.7, pp. 1450–1464.
